# Airway Secretory microRNAome Changes during Rhinovirus Infection in Early Childhood

**DOI:** 10.1371/journal.pone.0162244

**Published:** 2016-09-19

**Authors:** Maria J. Gutierrez, Jose L. Gomez, Geovanny F. Perez, Krishna Pancham, Stephanie Val, Dinesh K. Pillai, Mamta Giri, Sarah Ferrante, Robert Freishtat, Mary C. Rose, Diego Preciado, Gustavo Nino

**Affiliations:** 1 Division of Pediatric Allergy Immunology, Johns Hopkins University School of Medicine, Baltimore, Maryland, United States of America; 2 Division of Pulmonary, Critical Care and Sleep Medicine, Yale University School of Medicine, New Haven, Connecticut, United States of America; 3 Division of Pulmonary and Sleep Medicine, Children’s National Medical Center, Washington, DC, United States of America; 4 Department of Pediatrics, George Washington University School of Medicine and Health Sciences, Washington, DC, United States of America; 5 Department of Integrative Systems Biology and Center for Genetic Medicine Research, George Washington University, Washington, DC, United States of America; 6 Center for Genetic Medicine Research, Children’s National Medical Center, Washington, DC, United States of America; 7 Division of Pediatric Pulmonology, University of Kentucky, Lexington, Kentucky, United States of America; 8 Division of Pediatric Otolaryngology-Head and Neck Surgery, Children’s National Medical Center, Washington, DC, United States of America; 9 Division of Emergency Medicine, Children’s National Medical Center, Washington, DC, United States of America; Telethon Institute for Child Health Research, AUSTRALIA

## Abstract

**Background:**

Innate immune responses are fine-tuned by small noncoding RNA molecules termed microRNAs (miRs) that modify gene expression in response to the environment. During acute infections, miRs can be secreted in extracellular vesicles (EV) to facilitate cell-to-cell genetic communication. The purpose of this study was to characterize the baseline population of miRs secreted in EVs in the airways of young children (airway secretory microRNAome) and examine the changes during rhinovirus (RV) infection, the most common cause of asthma exacerbations and the most important early risk factor for the development of asthma beyond childhood.

**Methods:**

Nasal airway secretions were obtained from children (≤3 yrs. old) during PCR-confirmed RV infections (n = 10) and age-matched controls (n = 10). Nasal EVs were isolated with polymer-based precipitation and global miR profiles generated using NanoString microarrays. We validated our *in vivo* airway secretory miR data in an *in vitro* airway epithelium model using apical secretions from primary human bronchial epithelial cells (HBEC) differentiated at air-liquid interface (ALI). Bioinformatics tools were used to determine the unified (nasal and bronchial) signature airway secretory miRNAome and changes during RV infection in children.

**Results:**

Multiscale analysis identified four signature miRs comprising the baseline airway secretory miRNAome: *hsa-miR-630*, *hsa-miR-302d-3p*, *hsa- miR-320e*, *hsa-miR-612*. We identified *hsa-miR-155* as the main change in the baseline miRNAome during RV infection in young children. We investigated the potential biological relevance of the airway secretion of *hsa-mir-155* using *in silico* models derived from gene datasets of experimental *in vivo* human RV infection. These analyses confirmed that *hsa-miR-155* targetome is an overrepresented pathway in the upper airways of individuals infected with RV.

**Conclusions:**

Comparative analysis of the airway secretory microRNAome in children indicates that RV infection is associated with airway secretion of EVs containing miR-155, which is predicted *in silico* to regulate antiviral immunity. Further characterization of the airway secretory microRNAome during health and disease may lead to completely new strategies to treat and monitor respiratory conditions in all ages.

## Introduction

Immune responses are fine-tuned by small RNA molecules termed microRNAs (miRs) that modify gene expression in response to the environment. miRs comprise a large family of highly conserved, short, non-coding RNAs that regulate post-transcriptional gene-silencing through inhibition of translation or promotion of mRNA degradation.[1] miRs regulate approximately 60% of protein encoding genes.[2] They exist in body fluids, including saliva, nasal secretions, sputum, urine, breast milk, and blood.[3] To maintain their stability in extracellular body fluids, they are released in membrane-bound extracellular vesicles (EVs). miR-containing EVs are deemed important for genetic exchange and communication between cells.[4] Specifically, extracellular miRs are known to regulate key steps in cell proliferation, differentiation and migration and to play an important role in immune responses to infections, autoimmunity and cancer.[4]

Respiratory immune responses are fine-tuned by miRs. Resident and migrating lung immune cells such as macrophages, dendritic cells (DC), lymphocytes and airway epithelium and smooth muscle cells undergo post-translational regulation of immune-related genes via miRs.[5] Numerous miRs have been reported to have physiological roles in maintaining tissue homeostasis and normal development in the airways and the lung.[6, 7] There is compelling evidence demonstrating that several miRs also play pivotal roles in fine-tuning important pathogenic pathways including the regulation of the effector function of T helper (Th) 2 cells in allergic asthma,[8] the regulation of host defense immune responses,[9] and the repair and remodeling of the airways.[5] Despite the importance of miRs in the genetic regulation of the respiratory system, there is paucity of data describing the baseline population of miRs secreted in EV in the human airways (airway secretory miRNAome). The importance of investigating the airway secretory miRNAome is that it may have a powerful role in regulating cell-to-cell genetic communication through the entire respiratory system (from the nose to the small airways), particularly given the stability and mobility of EVs (and its miR cargo) in extracellular body fluids.[3, 10] Moreover, the dynamic regulation of the airway secretory miRNAome maybe a key mechanism during environmental exposures and acute infections, instances in which cell-to-cell communication is crucial to synchronize host immune defense and inflammatory signaling pathways.

The purpose of this study was to characterize the baseline population of miRs secreted in EVs in the airways of young children (airway secretory microRNAome) and examine the changes during rhinovirus (RV) infection. RV is the most common cause of asthma exacerbation in children and adults[11] and RV-induced wheezing illnesses during the first 3 years of life are the strongest risk factor (10 times increased odds) for the development of asthma beyond childhood.[12] Our central hypothesis was that RV infection in young children elicits distinctive signatures in the airway secretory microRNAome that may modulate the balance between Th1 antiviral immunity and Th2 pro-asthmatic responses during early life. The impact of our research is that it highlights the untapped potential of investigating the human airway secretory miRNAome during health and disease and it provides new insights into the potential immune regulatory role of virally induced miR secretion, which may ultimately enhance our knowledge on the early origins of asthma and may identify new strategies to treat and monitor a myriad of respiratory disorders in all ages.

## Materials and Methods

### Nasal washing collection and study subjects

Nasal airway secretions were collected in patients ≤3 yr. of age with PCR-confirmed RV infection (n = 10). All subjects were enrolled during the hospital admission for RV infection. Controls were age-matched children (n = 10) with non-detectable virus by PCR testing. Clinical and demographic variables were obtained by reviewing electronic medical records and presented in S1 Table. Sample was obtained while they were undergoing diagnostic nasal lavage (respiratory virus detection by PCR) at Children's National Medical Center.[13] RV positive (RV-infected group) or negative virus status (control group) was confirmed by a viral multiplex PCR panel for 12 targets (rhinovirus, RSV A, RSV B, HMPV, parainfluenza 1–3, influenza A and B, H1N1, H1N3, Adenovirus) used for clinical purposes (Luminex, TX, USA). We used a standard nasal lavage technique consisting of gently washing the nasal cavity with 3–4 mL sterile normal saline as previously described.[13] The Institutional Review Board (IRB) of Children’s National Medical Center, Washington D.C. approved the study and granted a waiver of informed consent given that this research involved materials (data, documents, records, or specimens) collected solely for non-research purposes (clinical indications).

### Extracellular vesicles isolation and characterization

Nasal exosomes were isolated with a polymer-based precipitation method (ExoQuick—System Biosciences, Mountain View, CA) according to manufacturer’s protocol.[14] Isolated exosomes were characterized by Western Blot (WB) using the harbor transpanin CD63 as exosomal marker. Anti-CD63 WB primary antibodies (System Biosciences, Mountain View, CA) were used at a 1: 1,000 dilutions and the HRP secondary antibody at 1: 20,000 dilutions. Exosomal quantification was performed with a commercially available kit (ExoCET method—System Biosciences, Mountain View, CA) that directly measures.[15] Exosomal particle size analysis was performed with a Dynamic Light Scattering (DLS) instrument (Zetasizer, Malvern Instruments, UK) and Nanoparticle Tracking Analysis (NTA) software (Malvern Instruments, UK) using the Stokes Einstein equation to calculate exosomal hydrodynamic diameters.[16]

### Extracellular microRNA profiling

The RNA contained in extracellular vesicles/exosomes was isolated and purified using a phenol-free lysis buffer and rapid spin columns (SeraMir kit System Biosciences, Mountain View, CA). We performed RNA separation, detection and quantitation with the Agilent Small RNA Kit and a Bioanalyzer instrument (2100 Bioanalyzer, Agilent Technologies, Santa Clara, CA). The global microRNAs (miRs) profile was obtained using NanoString human microarrays (human V2 miRNA array >800 probes, Nanostring Technologies, Seattle, WA). To account for differences in hybridization and purification, data were normalized to the average counts for all control spikes in each sample using a proprietary bioinformatics software (nSolver™ Analysis Software 2.5, Nanostring Technologies, Seattle, WA). Briefly, we calculated a background level of expression for each sample using the mean level of the negative controls plus two standard deviations of the mean. MiRNA expressing less than two standard deviations from the mean were set to 0 expression. Those miRNAs that were considered non-zero expression, were normalized using a scaling factor based on the top 100 expressing miRNAs across all samples. For each sample, the average of the geometric means of the top 100 expressing miRNAs across all samples was divided by the geometric mean of each sample.[17]

### Isolation of secreted extracellular vesicles from air-liquid interface differentiated human bronchial epithelial cells

Nasal miRs data were contrasted with normal airway epithelial secretions obtained *in vitro* from the apical side of air-liquid interface (ALI)-differentiated human bronchial epithelium.[18] Human bronchial epithelial cells (HBEC) were purchased from Lonza, Walkersville, MD (Catalog number CC-2540, Lonza Inc., Switzerland). HBEC were amplified on collagen-coated T-75 flasks as previously described,[19] then plated apically on type IV collagen coated 12 well transwell plates (Fisher Scientific, Pittsburgh, PA), grown submerged for 7–10 days until 100% confluence. Apical media was removed and cells differentiated at air-liquid interface (ALI). After 20 days at ALI, cells were gently washed 4 times with PBS apically and baso-laterally and protein-free BEBM was added to the basal side. Apical secretions were removed and extracellular vesicles isolated and characterized as described above. Small RNA separation, detection and quantitation was performed with the Agilent Small RNA Kit chip (2100 Bioanalyzer, Agilent Technologies, Santa Clara, CA) and miRs profiled using NanoString human microarrays (800 probes) (Nanostring Technologies, Seattle, WA).

### Bioinformatics and Statistical analysis

Biological network analysis was conducted using the identified baseline airway extracellular microRNAs (baseline airway miRNAome: *hsa-miR-630*, *hsa-miR-302d-3p*, *hsa- miR-320e*, *hsa-miR-612*.) and the *hsa-mir-155* targets overlapping the GSE11348 dataset (described below) with the use of QIAGEN’s Ingenuity Pathway Analysis (IPA, QIAGEN Redwood City, CA, www.qiagen.com/ingenuity). Overrepresented pathways were defined as those containing more targets than expected by chance, as calculated by the right-tailed Fisher’s exact test. Differences between groups were analyzed using unpaired T or Mann-Whitney U tests. A p-value <0.05 was considered significant.

### *hsa-miR-630*, *hsa-miR-302d-3p*, *hsa- miR-320e*, *hsa-miR-612 and hsa-mir-155* Targets

The miRTarBase release 4.5 (http://mirtarbase.mbc.nctu.edu.tw/index.php) was used to identify predicted targets of *hsa-miR-630*, *hsa-miR-302d-3p*, *hsa- miR-320e*, *hsa-miR-612 and hsa-mir-155*. Briefly, miRTarBase is a database of miRNA-target interactions (MTIs). This database is manually curated and enriched for MTIs validated experimentally by reporter assay, western blot, microarray and next-generation sequencing experiments.[20] The selection included targets for *Homo sapiens* -5p sequences. This list was used to identify *hsa-miR-630*, *hsa-miR-302d-3p*, *hsa- miR-320e*, *hsa-miR-612* for biological network analysis as described above. In addition, *hsa-mir-155* targets were used in combination with gene expression validation datasets to model the effect of RV infection *in vivo* as described below.

### Microarray Analysis of Gene Expression Omnibus GSE11348

To evaluate the effect of rhinovirus infection in human airway epithelium, the GSE11348 dataset was retrieved from the GEO database (http://www.ncbi.nlm.nih.gov/geo/). The GSE11348 is a study of gene expression profiles during in vivo human rhinovirus infection.[21] Genespring version 12.6 (Agilent Technologies, Santa Clara, CA) was used to analyze the dataset. The.CEL files were normalized using the RMA summarization algorithm with baseline transformation to median for all samples. Following microarray data pre-processing a one-way ANOVA test with the Tukey's honestly significance difference test was applied to identify differentially expressed transcripts between pre-infection, 8 hours and 48 hours post-experimental rhinovirus infection. Results of the ANOVA were corrected for multiple hypothesis testing (Benjamini-Hochberg). Statistical significance was defined as a false discovery rate (FDR) <5%. Transcripts with ≥1.2-fold change between conditions were selected for further analyses described below.

### *hsa-mir-155* Targetome in experimental in vivo human rhinovirus infection

R software (R: A Language and Environment for Statistical Computing. R Foundation for Statistical Computing, Vienna, Austria) was used for data analysis. R stats package version 3.0.1 was implemented. A custom script was used to overlap the *hsa-mir-155* miRTarBase transcripts above with the output following analyses of the GSE11348 dataset using the gene symbol according to the genome reference consortium human reference 38. Predicted *hsa-mir-155* were identified to characterize their temporal behavior following experimental RV infection.

## Results

### Determination of the baseline secretory airway miRNAome

In order to characterize the effect of RV infection in the airway secretion of extracellular miRs, we first determined the global profile of miRs secreted in extracellular vesicles/exosomes under basal conditions, which we refer here as the *baseline secretory airway miRNAome*. Because miRs have extensive regulatory functions in the airway, particularly in the development and function of the airway epithelium, [6, 7] we anticipated that a set of extracellular miRs would be secreted at high levels under normal conditions in all subjects studied. The importance of identifying the baseline secretory airway miRNAome is that it may serve as homeostatic background to investigate the presence of new extracellular miRs during pathological conditions. In our case, we were interested in the newly secreted miRs during RV infection.

**Fig 1:**
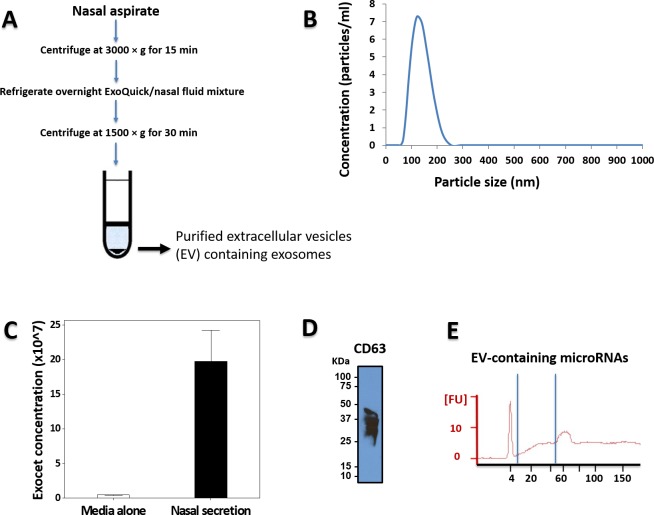
Isolation of extracellular vesicles (EV) from nasal secretions. A) Workflow of isolation method. B) Dynamic Light Scattering (DLS) Nanoparticle Tracking analysis identified secreted EV mostly in the 50–150 nm range. C) ExoCET (AChE activity assay) and D) CD63 western blotting of the isolated vesicles indicated that we had successfully isolated exosomes. E) Representative result from small RNA Bioanalyzer confirming the presence of miRs in the isolated EVs.

**Fig 2:**
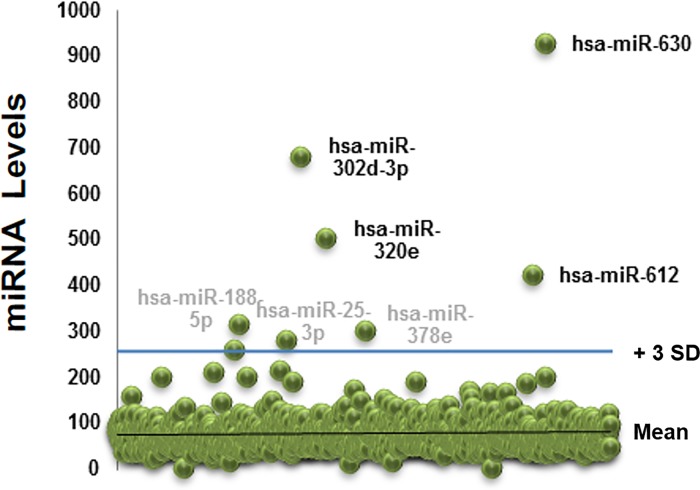
Nasal extracellular miRs profiles at baseline. Scattered plot of the nasal extracellular miRs profile of 10 children without detectable viral respiratory infection (baseline airway secretory miRNAome). SD = Standard deviation.

Our first step was to isolate extracellular miRs from the nasal secretions of 10 children without acute viral respiratory infection (controls). Fig 1 shows the workflow utilized including standard extracellular vesicles/exosome isolation with a polymer-based centrifugation method (Exoquick) and characterization based on particle size (DSL Nanotracking), AChE activity (ExoCET assay) and immune markers (CD 63; Fig 1B) as previously described.[16] We next isolated small RNA and confirmed the presence of miRs using Agilent Bioanalyzer (Fig 1C). Isolated small RNA was used to profile extracellular miRs with a NanoString panel containing > 800 human targets. The top 20 miRs more abundant (and present in all subjects) are presented in Table 1. S2 Table contains the total baseline extracellular miRs. To visualize better the baseline airway secretory miRNAome we built scattered plots that highlight the population of extracellular miRs significantly above background (> 3 SD from mean miR counts; Fig 2). This analysis identified 7 candidate miRs that were clearly above all the other miRs: *hsa-miR-630*, *hsa-miR-302d-3p*, *hsa- miR-320e*, *hsa-miR-612*, *hsa-miR-378e*, *hsa-miR-25-3p*, *hsa- miR-188-5p*.

**Table 1:** Top 20 baseline nasal airway extracellular miRs (n = 10 children).

Gene Name	Target Sequence	miRNA counts	
		Mean	SD
hsa-miR-630	AGUAUUCUGUACCAGGGAAGGU	930	98.8
hsa-miR-302d-3p	UAAGUGCUUCCAUGUUUGAGUGU	682	292
hsa-miR-320e	AAAGCUGGGUUGAGAAGG	502	69.5
hsa-miR-612	GCUGGGCAGGGCUUCUGAGCUCCUU	425	122
hsa-miR-188-5p	CAUCCCUUGCAUGGUGGAGGG	315.2	223.4
hsa-miR-378e	ACUGGACUUGGAGUCAGGA	303.5	96.5
hsa-miR-25-3p	CAUUGCACUUGUCUCGGUCUGA	282.3	91.4
hsa-miR-1827	UGAGGCAGUAGAUUGAAU	260.6	110
hsa-miR-222-3p	AGCUACAUCUGGCUACUGGGU	216	122.3
hsa-miR-144-3p	UACAGUAUAGAUGAUGUACU	213.2	18.5
hsa-miR-125b-5p	UCCCUGAGACCCUAACUUGUGA	203.1	139.8
hsa-miR-631	AGACCUGGCCCAGACCUCAGC	201.8	120
hsa-miR-192-5p	CUGACCUAUGAAUUGACAGCC	201.7	108.2
hsa-miR-297	AUGUAUGUGUGCAUGUGCAUG	191.7	117.8
hsa-miR-495	AAACAAACAUGGUGCACUUCUU	189.6	117.2
hsa-miR-601	UGGUCUAGGAUUGUUGGAGGAG	189.2	57.6
hsa-miR-371a-3p	AAGUGCCGCCAUCUUUUGAGUGU	175	96.4
hsa-miR-548ad	GAAAACGACAAUGACUUUUGCA	168.8	79.3
hsa-miR-570-3p	CGAAAACAGCAAUUACCUUUGC	167.9	81.3
hsa-miR-548x-3p	UAAAAACUGCAAUUACUUUC	165.7	76

**Fig 3:**
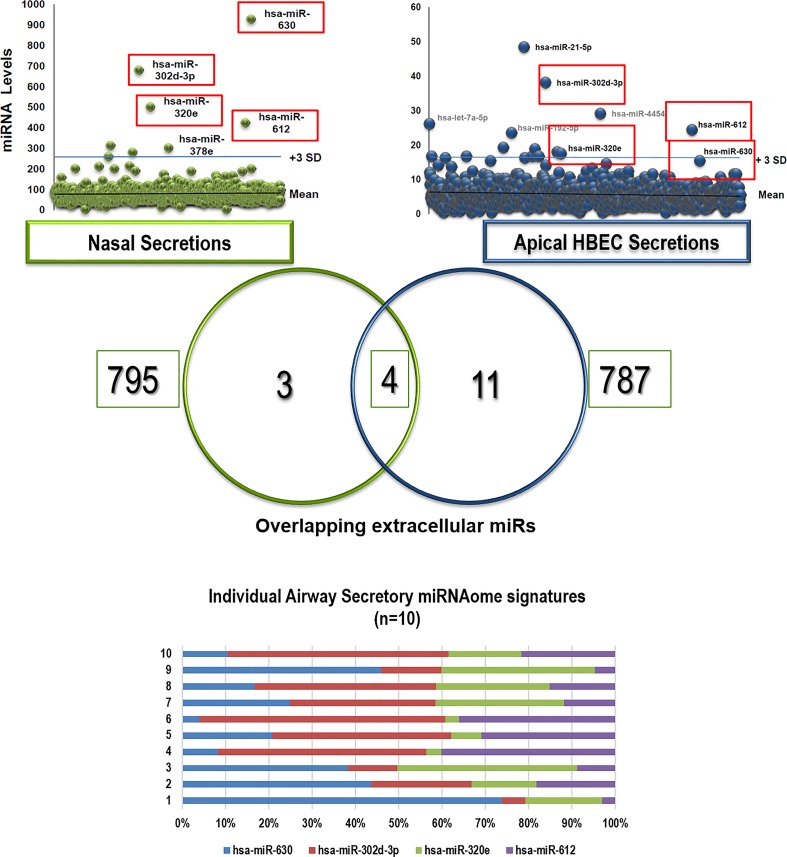
Multi-scale airway secretory miRs profiling comparing *in vivo* nasal miRs vs. *in vitro* miRs isolated from the apical secretions of ALI-differentiated human bronchial epithelial cells (HBEC). Venn diagram identified 4 overlapping extracellular *hsa-miR-630*, *hsa-miR-302d-3p*, *hsa- miR-320e*, *hsa-miR-612* (red squares; baseline airway epithelial miRNAome). Stacked normalized bars show individual baseline airway epithelial miRNAome profiles (n = 10 children)

**Fig 4:**
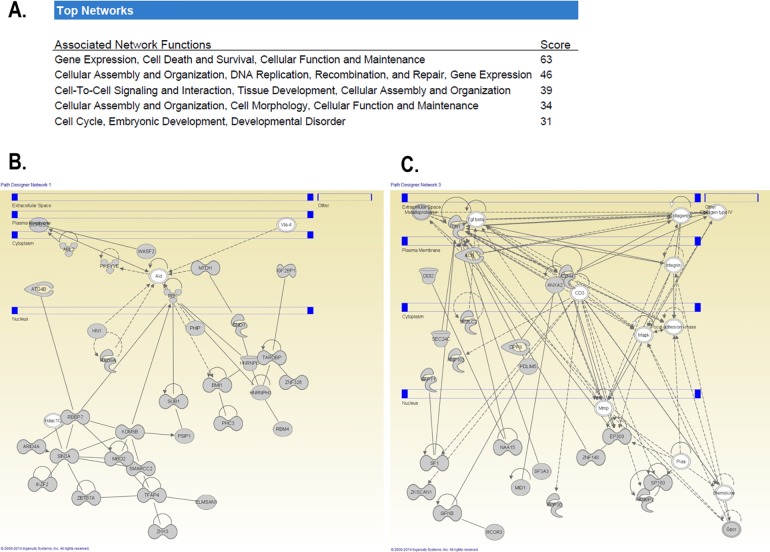
IPA pathway analysis of predictive targets of the baseline airway secretory miRNAome. IPA analysis of *hsa-miR-630*, *hsa-miR-302d-3p*, *hsa- miR-320e*, *hsa-miR-612* identified cellular assembly, organization, development and repair as top functions (A) and overrepresented gene networks for AKT (B) TGF beta, MMP and MAPK signaling (C).

Given that *in vivo* nasal washes reflect a mixed secretion (naso-oropharynx) that it is susceptible to contamination by environmental particles (either in the nose or introduced during the collection), we decided to validate our *in vivo* airway exosomal miRNAome findings using an *in vitro* model of the human airway epithelium. Analogous to what we have previously described with the airway secretome[19, 22] and directional immune profiling,[18] for these experiments we cultured human bronchial epithelial cells (HBEC) differentiated at ALI and collected apical secretions to obtain a representative “clean” sample of the unified (nasal and bronchial) airway secretory miRNAome. Next, we profiled extracellular vesicles/exosomes in the apical secretions of ALI-differentiated bronchial epithelium and overlapped these data with our *in vivo* findings. As shown in Fig 3, we found astonishing similarities between the *in vivo* and *in vitro* airway miRNAome. In fact, the top 4 extracellular miRs found initially in nasal secretions (*hsa-miR-630*, *hsa-miR-302d-3p*, *hsa- miR-320e*, *hsa-miR-612*.*)* were also found in abundant top levels in our *in vitro* airway epithelial model (scattered plots and Venn diagram in Fig 3). We concluded that these four miRs are the main signature of the baseline airway secretory miRNAome, which is found constitutively in the nasal secretions of young children. Notably, although this baseline airway secretory miRNAome was present at high concentrations in all subjects, the specific composition varied among individuals (Fig 3) indicating that this homeostatic population of miRs has a dynamic range among different subjects and perhaps overtime within individuals. Bioinformatics analysis of the predictive targets of the airway secretory miRNAome (enriched for epithelial expression) showed cellular assembly, organization, development and repair as top functions (Fig 4A). Moreover, Ingenuity pathway analysis (IPA) of these miRs identified several targets involved in epithelial remodeling and mesenchymal differentiation via regulation of protein kinase B (Akt), transforming growth factor beta (TGFβ), mitogen-activated protein kinase (MAPK) signaling. The identified overrepresented networks with IPA can be visualized in Fig 4B. Collectively, these results re-enforced the notion that the baseline extracellular miR secretion may play in role in the homeostasis of the airways modulating key pathways involved in the differentiation, repair and remodeling of the airways. [5–7]

### Effect of RV infection in the airway secretory microRNAome

**Fig 5:**
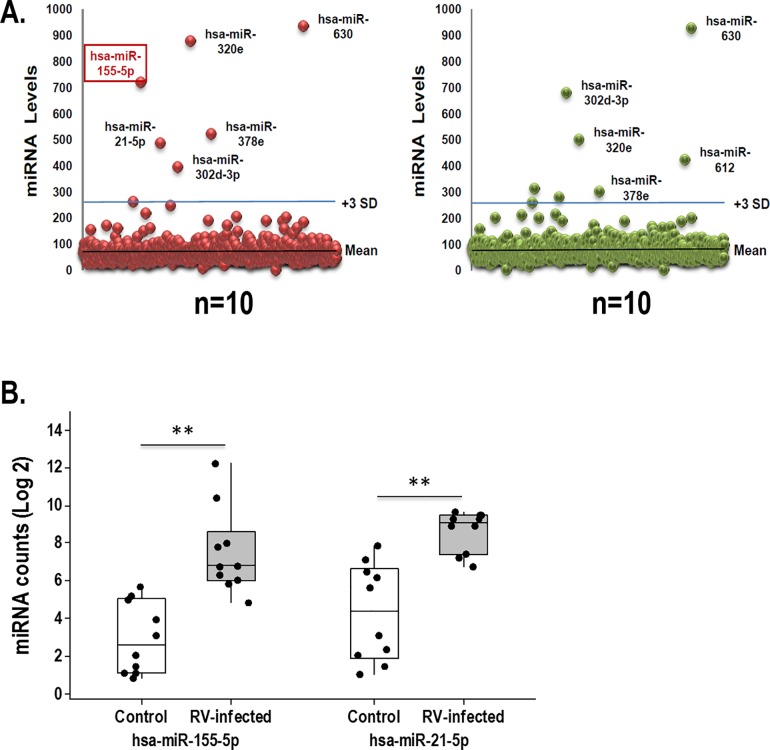
Nasal extracellular miRs profiles during rhinovirus infection. (A) Scattered plot of nasal airway extracellular miRs (control vs. rhinovirus) airway show similar baseline miRNAnome (*hsa-miR-630*, *hsa-miR-302d-3p*, *hsa- miR-320e*, *hsa-miR-612* miR) in rhinovirus (RV)-infected and control uninfected children except for the presence of *hsa-mir155* (red square) and *hsa-mir21* in individuals with RV. (B) Boxplots depicting individual levels of *hsa-mir155* and *hsa-mir21* (log 2) and 25–75 percentiles. ** p<0.01

We next examined the airway secretory miRNAome profiles in children with PCR-confirmed RV infection (n = 10). As expected, we identified abundant levels of *hsa-miR-630*, *hsa-miR-302d-3p*, *hsa- miR-320e*, *hsa-miR-612* (baseline miRNAome) in the nasal airway secretions of all children (Fig 5). S3 Table contains all the airway extracellular miRs identified in the nasal secretions of children with RV infection. As shown in the summarized scattered plots, we did not identify significant differences in the relative abundance of the baseline miRNAome during RV infection (Fig 5), although children infected with RV trend to have higher *hsa-miR-320e* levels and lower *hsa-miR-612* counts. In contrast, we identified the unequivocal new presence of *hsa-miR-155* during RV infection. *hsa-miR-155* levels were clearly above the background, being as high as those seen in the baseline miRNAome (Fig 5A and Fig 5B). It is noteworthy that *hsa-miR-155* was not present at these levels in any of the subjects without RV infection. Another difference in the miRNAome composition of children infected with RV was the presence of *hsa-miR-21*, (Fig 5), which was also present at significantly high levels. However, in contrast to *hsa-miR-155*, which was exclusively linked to RV infection, *hsa-miR-21* was also part of the baseline extracellular miRs secreted by differentiated airway epithelium *in vitro* (Fig 3).

### Dynamic regulation of mir-155 targetome during experimental in vivo human RV infection

To evaluate the potential regulatory effect of *hsa-miR-155* on the airway transcriptome during *in vivo* RV infection, the GSE11348 dataset was retrieved from the GEO database (http://www.ncbi.nlm.nih.gov/geo/). The GSE11348 is a study of gene expression profiles during *in vivo* human RV infection.[21] This study of nasal epithelial scrapings before and during experimental RV infection was relevant to cross-validate *in silico* our observations seen on nasal secretions from children infected with RV. We focused on the targetome analysis of *hsa-miR-155*, given our current observations and recent evidence demonstrating the antiviral effect of *hsa-miR-155* against RV *in vitro*.[23]

**Fig 6:**
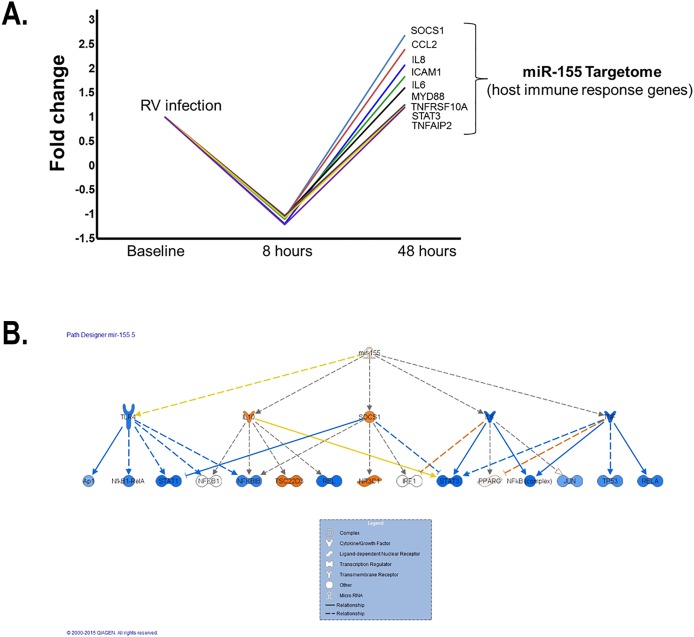
*In silico* model of the dynamic regulation of mir-155 targetome during experimental *in vivo* human RV infection. (A) Early downregulation (8hr) of validated *hsa-miR-155* targets implicated in host immune response followed by upregulation at 48h (B). IPA network analysis identified overrepresented targets for *hsa-mir-155* at 8hr after RV infection.

The initial *hsa-miR-155* miRTarBase list of predicted targets identified a total of 841 records for predicted targets of *hsa-miR-155*, which represented 723 unique genes. The two most common validation methodologies to validate these targets were proteomics (52% of the targets) and reporter assay (23% of the targets). We overlapped the *hsa-miR-155* miRTarBase list of predicted targets with the filtered output from the analysis of the GSE11348 dataset. A total of 81 genes were identified as part of the *hsa-miR-155* targetome during experimental *in vivo* human RV infection (presented in S4 Table). Our *in silico* analysis demonstrated a potential complex effect of *hsa-miR-155* during RV infection, with a dynamic regulation at 8h and 48 h. At 8 hours the largest changes recorded included the DPP7 gene, which was upregulated 1.1 fold, and the NAMPT, IL-8 and TNFAIP2 genes downregulated 1.2 fold each. At 48 hours NAMPT, IL-8 and TNFAIP2 were upregulated 1.4, 2.1, 1.2 fold, respectively, while the DPP7 gene was downregulated 1.4 fold. Interestingly, most of the genes targeted by *hsa-miR-155* that are implicated in the regulation of the host immune response to RV underwent early silencing at 8hr but subsequent upregulation at 48h (Fig 6A). The SOCS1 gene had the largest change between the two time periods with a 2.7-fold upregulation at 48 hours from a 1.1-fold downregulation at 8 hours.

To further evaluate the temporal changes in genes targeted by the *hsa-mir-155* following experimental RV infection in the human upper airway response we performed a pathway analysis and identified overrepresented networks with IPA. Importantly, at 8 hours the top upstream regulator for the list was *hsa-mir-155* (p = 5.46x10^-26^) with 19 genes included. The direction of the regulatory effect exerted by *hsa-mir-155* on these molecules at 8 hours was concordant with the literature for the downregulation of *ANKFY1*, *BACH1*, *MYD88*, *CEBPB*, *MATR3*, *IL6*, *TNFRSF10A*, *FMNL2*, *SOCS1*, *MECP2 and CXCL8*. Fig 6B illustrates the changes at 8 and 48 hours post-infection for the *hsa-mir-155* pathway in the genes captured on the analysis. At 48 hours the top upstream regulator for the list was still *hsa-mir-155* (p = 5.23x10^-28^) with 21 genes included. Two additional pathways activated at 48 hours included TGFB1 (p = 1.1x10^-15^) and response to dsRNA (p = 4.99x10^-15^). The direction of the regulatory effect exerted by *hsa-mir-155* on these molecules at 48 hours was concordant with the literature for the upregulation of *CCL2*, *CXCL8*, *HK2 and STAT3*.

## Discussion

There is compelling evidence demonstrating that microRNAs (miR) modify gene expression in the airways and lungs[5, 24, 25] and is well-established that they represent a powerful mechanism that regulate normal and pathogenic responses to numerous respiratory environmental challenges.[26–28] More recently, it has been increasingly clear that some miRs are selectively sorted, packaged and exported in protective membrane-bound extracellular vesicles (EV) that provide stability and mobility to the miR cargo,[3, 4, 10] allowing genetic communication between distant cells.[4] EVs containing miRs have been successfully isolated from nasal[29] and pulmonary secretions[30] as well as from resident and migrating individual lung immune cells such as macrophages,[31, 32] dendritic cells (DCs), lymphocytes and airway epithelium.[33, 34] Moreover, the functional transferring of miRs via EVs, and consequent cell-to-cell genetic reprogramming, has been confirmed in animal models and several cell systems[35, 36] including human airway epithelial cells exposed to cigarette smoke.[31] Collectively, this solid scientific evidence highlights the biological importance and untapped potential of investigating the mechanisms and function of the airway secretion of extracellular miRs during health and disease. Nonetheless, there is paucity of data characterizing the global population of extracellular miRs in human respiratory secretions, which we refer here as the *human airway secretory microRNAome*. The purpose of this study was to begin addressing this critical gap in the field characterizing *in vivo*: 1) the baseline miRs secreted in EVs in the airways of young children and; 2) the microRNAome changes during rhinovirus (RV) infection.

Our initial experiments examined the baseline secretory airway miRNAome. After isolation of EVs and purification of small RNA, we determined the global miR profile in the nasal secretion of young children (< 3yrs old). We used this age group because long-term airway remodeling[37] and subsequent asthma risk[12] is largely determined during the first 3 years of life,[12] suggesting that this early developmental window is a critical period for airway genetic re-programming. Our studies identified a baseline population of miRs in the nasal airway secretions of all the children included (Fig 2). The top 4 extracellular nasal miRs were also found in abundant top levels in our *in vitro* airway epithelial model (*hsa-miR-630*, *hsa-miR-302d-3p*, *hsa- miR-320e*, *hsa-miR-612*), suggesting that these four secreted miRs comprise a signature airway secretory miRNAome. The most abundant extracellular miR was *hsa-miR-630*, which has been reported to control airway epithelial cell death and survival, maintaining a complex regulation of its cell cycle and apoptotic balance.[38] Specifically, in A549 cells, miR-630 inhibits cell proliferation by targeting cell-cycle kinase 7 (CDC7) kinase, but at the same time inhibits multiple activators of apoptosis under genotoxic stress.[38] The importance of maintaining synchronous cell cycle/apoptosis under stress conditions is highlighted by our recent studies demonstrating that mitotic asynchrony in repairing tissue promotes chronic inflammation and fibrosis via up-regulation of transforming growth factor beta (TGFβ) signaling.[39] Interestingly, when we examined the collective predictive function of the airway secretory miRNAome, including *hsa-miR-630*, *hsa-miR-302d-3p*, *hsa- miR-320e*, *hsa-miR-612* targets, we identified again cell death and survival as top functions, in addition to overrepresented gene pathways for TGFβ signaling and other regulators of airway epithelial remodeling (Fig 4B). These results are in overall agreement with our previous work defining the human airway secretome, in which we also found cell death and survival as the top collective functions of the apical secretions of the human airway epithelium.[18] Taken together, our results suggest that the baseline airway secretory miRNAome may contribute to maintaining a cell death/survival balance in the human epithelial barrier, providing a secretory miR homeostatic program that might coordinate repair and remodeling of the airways under stress conditions.

RV is the most common cause of asthma exacerbations[11] and the most important early risk factor for the development of asthma beyond childhood.[11, 12] Our data indicate that acute RV infection in young children is associated with airway secretion of EV containing *hsa-miR155*. As shown in Fig 5, *hsa-miR155* clearly emerged from the baseline background miR population at top high levels in young children infected with RV. There are no prior studies *in vivo* showing similar findings. However, *in vitro* studies have demonstrated that *has-miR155* transfection suppresses RV replication in the human cell line BEAS-2B (derived from normal human bronchial epithelial cells).[23] In this elegant work Bondanese and colleagues also observed viral RNA co-immunoprecipitated with argonaute 2 protein (crucial component of the miR silencing complex) confirming the functional direct action of miRs against RV. In agreement with our current findings, bioinformatics predictions and subsequent experiments demonstrated that *hsa-miR155* is the key miR orchestrating host immune responses against RV.[23] Similarly, miR-155 has been previously identified by other groups to be a key player in antiviral responses in the respiratory system[5, 40] as well in other systems,[41–43] being critical for host defense against numerous viruses such as influenza, hepatitis C, herpes and HTLV-1 infections. [9, 44, 45]

Notwithstanding the importance of miR-155 in the regulation of antiviral immunity, there are two additional features that make this molecule very intriguing and important in human airway immunology. The first is that miR-155 is selectively sorted, packaged and exported in exosomes during immune responses.[1, 4] In fact, miR-155 is the prototype cargo molecule for exosome-mediated immune regulation in several cell systems[1] and is currently being studied as top candidate for potential miR-driven immune therapies via exosomes.[46] In this context, it is important to mention that one of the strongest pieces of evidence comes from a recent seminal work from Alexander and colleagues, in which miR-155 released from primary bone marrow-derived DCs (BMDCs) in exosomes were taken up by recipient BMDCs and subsequently induced complete target gene repression *in vitro* and *in vivo*. [47] A second intriguing feature of miR-155 is that despite being a robust enhancer of Th1 antiviral responses, it is also needed for the development of allergic Th2 responses.[48] Several studies have identified that miR-155 is essential for Th2-mediated eosinophilic inflammation in the lung,[28] which maybe due to the fact that miR-155(-/-) DCs have limited Th2 priming capacity[49] and that CD4 (+) Th2 cells require intrinsic miR-155 expression for type-2 immune polarization.[48] Complementing these animal studies, human based research has shown that miR-155 modulates the response of human macrophages to IL-13, a crucial cytokine in the programming of Th2 responses,[50] and that miR-155 levels are dysregulated in Th2-driven conditions such as asthma and allergic rhinitis.[8] Collectively, these data indicate that miR-155 has a powerful and unique dual role in airway immunology, fine-tuning Th1 (antiviral) and Th2 (allergic) inflammatory responses. Our current study provides *in vivo* evidence of the airway secretion of EV containing *hsa-miR155* during natural RV infection in young children. This new knowledge proves the relevance of miR-155 for human airway immunobiology and highlights the need for further studies dissecting the potential role of miR-155 in modulating the balance between Th1 antiviral immunity and Th2 pro-asthmatic responses during RV infections. Elucidating this notion may provide novel insights into the mechanisms by which RV induces asthma exacerbations and increases the risk of asthma beyond childhood.[11, 12]

The target gene(s) that mediate(s) the effect of miR-155 in the airways are not completely clear. Prior studies have demonstrated that miR-155 acts as a positive feedback regulator in antiviral immune responses by targeting SOCS-1. [9] It can also act as a negative regulator of SHIP[1], hence enhancing type I interferon (IFN) signaling. Additional targets have been implicated in the regulatory effect of miR-155 in Th2 responses including ENTPD,[49] S1PR1[48] and the transcription factor PU.1.[51] However, it is unlikely that the effects of miR-155 are mediated by single gene downregulation. Seminal experiments establishing the miR-155 induced global proteome changes by LC-MS/MS-based proteomics [52] identified that hundreds of proteins with miR-155 seed sequences tend to be downregulated simultaneously during miR-155 overexpression. Interestingly, this repression was relatively mild, indicating that the widespread changes in protein synthesis induced by miR-155 are the result of numerous small/moderate effects rather than a single gene effect.[53] In line with this notion, we designed an *in silico* study to examine the dynamic changes of the *miR-155 targetome* (all transcripts with *hsa-miR155* seed sequences previously validated as miR-155 targets; S3 Table) during *in vivo* human RV infection. For this analysis we used publicly available datasets containing nasal epithelial transcriptomes before and during experimental RV infection in humans (GSE11348), which were relevant to cross-validate *in silico* our observations seen on the nasal airway microRNAome. As shown in Fig 6, following experimental RV infection we observed a wave of small/moderate downregulation of the host immune response genes part of the miR-155 targetome with a peak effect at 8hrs and subsequent normalization or/and upregulation by 48 hrs. These results re-enforced the relevance of miR-155 during *in vivo* human RV infection. Additional work is needed to examine the potential functional role (inhibiting viral replication, amplifying IFN signaling and/or modulating Th2 immune responses) of the airway secretion of extracellular miR-155 during infections caused by RV and other respiratory viruses.

In addition to miR-155 we also identified the presence of miR-21 in the airway secretory miRNAome during RV infection. It is interesting that the parallel production of miR-155 and miR-21 has been reported before in several cell systems.[54, 55] Indeed, miR-155 and miR-21 are considered to have a synergistic effect on increasing STAT3 activity by targeting SHIP1 and PTEN, respectively. [56] Other groups have reported that the parallel secretion of miR-155 and miR-21 is important for the regulation of Toll-like receptor 4 (TLR4) signaling via a cross-talk SHIP1 and PDCD4 downregulation.[57–59] In our study the levels of miR-155 and miR-21 did not show significant correlation, however, this does not exclude the possibility that miR-21 influences miR-155 targetome. Interestingly, we found EV containing miR-21 in the apical secretions of our *in vitro* model of bronchial airway epithelium in the absence of RV infection (Fig 3), suggesting that miR-21 secretion may not be an specific response to RV but rather the result of secondary airway stress conditions such as hypoxia[60] or widespread exposure to pro-inflammatory cytokines.[61] However, our *in vitro* studies need to be interpreted with caution given that we did not assess miR changes after RV infection and we used HBEC lines that may not entirely reflect human airway epithelial responses *in vivo*.[62] In addition, and as a general limitation of the present study, we need to consider the cross-sectional nature our findings during RV infection. Indeed, miR-155 and miR-21 airway secretion could be transient and/or related to a specific the stage of the infection (e.g. recent onset vs. resolution phase). Longitudinal data with a larger number of patients may be needed to validate our findings. This type of studies would also be useful to address additional questions, including what cell(s) ultimately produce airway extracellular miR during RV infection. Although the airway epithelium plays a key role in mediating innate airway immune responses against RV, [63, 64] it is unclear whether they are the primary source of EVs containing *hsa-miR-155*. Indeed, immune cells such as DCs and Innate lymphoid cells (ILCs) are increasingly recognized key players in the regulation of airway immune responses during RV infection[65, 66]and they belong to a blood cell lineage with remarkable capability of releasing exosomes containing miR-155.[67, 68] Nevertheless, regardless of the origin, our findings reporting the airway secretion of EVs containing *hsa-miR155* during RV infection have a significant impact in the field, providing *in vivo* validation for the compelling data showing the pivotal role of miR-155 during RV infection *in vitro[23]* and the increasing evidence demonstrating that miR-155 may regulate Th1 and Th2 immunity directly[44, 48, 49] and via exosomal-mediated cell-to-cell genetic reprograming in several cell systems.[47]

## Conclusion

Our study identified four extracellular EV-containing miRs (*hsa-miR-630*, *hsa-miR-302d-3p*, *hsa- miR-320e*, *hsa-miR-612*) that constitute a signature miRNAome present at high concentrations in the airway secretions of all individuals included in this study (n = 20). We also identified *hsa-miR-155* as the main change in the baseline airway secretory miRNAome during RV infection in young children. Interestingly, miR-155 has major roles in exosome-mediated immune regulation[1, 47, 69] and in fine-tuning of both, Th1 (antiviral) and Th2 (allergic) pro-asthmatic inflammatory responses. [9, 28, 48, 49] The approaches and findings of this study indicate that further characterization of the airway secretory microRNAome during health and disease states may ultimately lead to completely new strategies to treat and monitor respiratory conditions in all ages.

## Supporting Information

S1 Table Baseline characteristics for subjects. (DOCX)

S2 Table Nasal airway extracellular miRs in control (CT) children (n = 10). (DOCX)

S3 Table Nasal airway extracellular miRs in RV infected subjects (n = 10 children). (DOCX)

S4 Table hsa-mir-155 Targetome in experimental in vivo human rhinovirus infection. (DOCX)
