# Supplementary material for: Airway Secretory microRNAome Changes during Rhinovirus Infection in Early Childhood
Source: PLoS One. 2016 Sep 19;11(9):e0162244. doi: 10.1371/journal.pone.0162244 (PMC5028059; doi:10.1371/journal.pone.0162244)
Supplement: S1 Table — (DOCX) [file pone.0162244.s001.docx]

**TABLE S1 .Baseline characteristics for subjects**

|  | Control  n=10 | Rhinovirus  n=10 | p-value |
| --- | --- | --- | --- |
| Male, n (%) | 6 (60) | 5 (50) | 0.65 |
| Age (years), median (IQR) | 1.2 (0.2-3) | 1.4 (0.4-3) | 0.82 |
| Black, n (%) | 4 (40) | 5 (50) | 0.65 |
| Family history of asthma, n (%) | 1 (10) | 2 (20) | 0.53 |
| Atopy, n (%) | 0 (0) | 1 (10) | 0.29 |

Demographics for all study subjects (n=20) with acute rhinovirus infection vs. control (non-detectable virus). *IQR*, interquartile range. P-values based on Wilcoxon rank-sum test for continuous variables; χ^2^ test for categorical.
