# Supplementary material for: Airway Secretory microRNAome Changes during Rhinovirus Infection in Early Childhood
Source: PLoS One. 2016 Sep 19;11(9):e0162244. doi: 10.1371/journal.pone.0162244 (PMC5028059; doi:10.1371/journal.pone.0162244)
Supplement: S2 Table — (DOCX) [file pone.0162244.s002.docx]

**Table 2 S Nasal airway extracellular miRs in control (CT) children (n=10)**

|  | **CT1** | **CT2** | **CT3** | **CT4** | **CT5** | **CT6** | **CT7** | **CT8** | **CT9** | **CT10** |
| --- | --- | --- | --- | --- | --- | --- | --- | --- | --- | --- |
| hsa-miR-630 | 4017.77 | 1424.97 | 1278.04 | 164.26 | 396.1 | 121.23 | 391.37 | 211.33 | 1195.03 | 98.76 |
| hsa-miR-302d-3p | 292 | 751.82 | 382.18 | 972.95 | 787.12 | 1734.81 | 527.66 | 528.31 | 361.94 | 482.83 |
| hsa-miR-320e | 967.94 | 489.56 | 1386.94 | 69.5 | 132.03 | 96.15 | 468.25 | 329.93 | 925.19 | 159.12 |
| hsa-miR-612 | 162.22 | 594.46 | 291.77 | 808.68 | 589.07 | 1103.59 | 185.2 | 191.92 | 122.07 | 205.75 |
| hsa-miR-188-5p | 239.73 | 358.43 | 462.31 | 227.44 | 223.44 | 430.57 | 262.08 | 286.8 | 291.26 | 370.36 |
| hsa-miR-378e | 751.64 | 131.13 | 410.94 | 151.63 | 96.49 | 146.31 | 178.21 | 467.93 | 368.36 | 331.95 |
| hsa-miR-25-3p | 486.67 | 183.58 | 719.15 | 132.67 | 91.41 | 246.64 | 108.33 | 299.74 | 233.44 | 320.98 |
| hsa-miR-1827 | 109.95 | 454.59 | 203.42 | 429.61 | 467.19 | 259.18 | 164.24 | 178.98 | 152.06 | 186.55 |
| hsa-miR-222-3p | 225.31 | 271.01 | 404.78 | 208.49 | 167.58 | 242.46 | 122.3 | 222.11 | 137.06 | 159.12 |
| hsa-miR-144-3p | 113.56 | 192.33 | 18.49 | 44.22 | 101.56 | 146.31 | 241.11 | 357.96 | 310.54 | 606.29 |
| hsa-miR-125b-5p | 239.73 | 192.33 | 168.49 | 227.44 | 198.05 | 213.19 | 139.78 | 310.52 | 182.04 | 159.12 |
| hsa-miR-631 | 236.13 | 157.36 | 386.29 | 120.04 | 126.95 | 137.95 | 160.74 | 232.89 | 229.16 | 230.44 |
| hsa-miR-192-5p | 108.15 | 174.84 | 117.12 | 309.57 | 253.91 | 267.54 | 244.61 | 209.17 | 145.63 | 186.55 |
| hsa-miR-297 | 178.45 | 244.78 | 328.76 | 164.26 | 126.95 | 242.46 | 136.28 | 185.45 | 117.79 | 192.04 |
| hsa-miR-495 | 117.16 | 157.36 | 149.99 | 183.22 | 126.95 | 200.65 | 272.56 | 235.05 | 214.16 | 238.67 |
| hsa-miR-601 | 349.68 | 305.97 | 421.22 | 94.77 | 182.81 | 75.24 | 153.75 | 86.26 | 164.91 | 57.61 |
| hsa-miR-371a-3p | 165.83 | 253.52 | 380.12 | 107.4 | 101.56 | 229.92 | 132.79 | 122.91 | 96.37 | 159.12 |
| hsa-miR-548ad | 79.31 | 192.33 | 90.41 | 296.94 | 192.97 | 146.31 | 209.66 | 170.35 | 128.5 | 181.06 |
| hsa-miR-570-3p | 248.74 | 87.42 | 258.89 | 138.99 | 81.25 | 137.95 | 150.26 | 215.64 | 184.18 | 175.58 |
| hsa-miR-548x-3p | 81.11 | 209.81 | 76.02 | 284.3 | 284.38 | 175.57 | 129.29 | 129.38 | 139.21 | 148.14 |
| hsa-miR-1183 | 70.3 | 183.58 | 78.08 | 214.81 | 198.05 | 158.85 | 157.25 | 174.67 | 143.49 | 211.24 |
| hsa-miR-548ah-5p | 86.52 | 174.84 | 100.68 | 265.35 | 269.14 | 188.11 | 76.88 | 97.04 | 199.17 | 104.25 |
| hsa-miR-4421 | 66.69 | 174.84 | 113.01 | 277.98 | 314.85 | 158.85 | 108.33 | 90.57 | 111.37 | 106.99 |
| hsa-miR-375 | 30.64 | 43.71 | 55.48 | 113.72 | 142.19 | 129.59 | 307.51 | 204.86 | 233.44 | 235.93 |
| hsa-miR-2117 | 73.9 | 183.58 | 78.08 | 246.4 | 238.67 | 158.85 | 181.71 | 114.29 | 92.09 | 126.2 |
| hsa-miR-149-5p | 149.61 | 131.13 | 176.71 | 107.4 | 137.11 | 142.13 | 118.81 | 157.42 | 132.78 | 194.78 |
| hsa-miR-448 | 64.89 | 157.36 | 59.59 | 202.17 | 187.89 | 137.95 | 185.2 | 129.38 | 139.21 | 164.6 |
| hsa-miR-579 | 160.42 | 139.87 | 110.95 | 56.86 | 96.49 | 87.79 | 174.72 | 202.7 | 184.18 | 175.58 |
| hsa-miR-361-3p | 187.46 | 122.39 | 283.55 | 120.04 | 76.17 | 200.65 | 111.82 | 99.19 | 96.37 | 79.56 |
| hsa-miR-520b | 61.28 | 192.33 | 108.9 | 227.44 | 198.05 | 221.55 | 129.29 | 66.85 | 72.82 | 96.02 |
| hsa-miR-4516 | 243.34 | 174.84 | 90.41 | 63.18 | 116.8 | 75.24 | 199.18 | 135.85 | 207.74 | 60.35 |
| hsa-miR-548al | 34.25 | 61.19 | 34.93 | 88.45 | 101.56 | 71.06 | 220.15 | 213.48 | 239.86 | 301.77 |
| hsa-miR-1286 | 72.1 | 113.65 | 94.52 | 176.9 | 228.52 | 179.75 | 118.81 | 138.01 | 92.09 | 134.43 |
| hsa-miR-574-5p | 100.94 | 122.39 | 123.28 | 151.63 | 111.72 | 79.43 | 111.82 | 247.98 | 149.91 | 139.91 |
| hsa-miR-211-5p | 90.12 | 131.13 | 94.52 | 284.3 | 304.69 | 175.57 | 69.89 | 47.44 | 81.38 | 57.61 |
| hsa-miR-2053 | 86.52 | 157.36 | 78.08 | 176.9 | 228.52 | 129.59 | 139.78 | 135.85 | 74.96 | 115.22 |
| hsa-miR-489 | 151.41 | 166.1 | 238.35 | 69.5 | 106.64 | 146.31 | 80.37 | 125.07 | 113.51 | 115.22 |
| hsa-miR-514b-5p | 124.37 | 131.13 | 242.46 | 88.45 | 106.64 | 117.05 | 108.33 | 127.23 | 143.49 | 117.97 |
| hsa-miR-548p | 70.3 | 104.91 | 160.27 | 101.09 | 111.72 | 129.59 | 146.77 | 155.26 | 167.05 | 148.14 |
| hsa-miR-223-3p | 187.46 | 87.42 | 501.35 | 75.81 | 66.02 | 45.98 | 87.36 | 129.38 | 42.83 | 63.1 |
| hsa-miR-3934 | 68.49 | 157.36 | 63.7 | 221.12 | 213.28 | 121.23 | 136.28 | 97.04 | 98.52 | 104.25 |
| hsa-miR-553 | 70.3 | 148.62 | 65.75 | 170.58 | 253.91 | 167.21 | 104.83 | 75.47 | 107.08 | 115.22 |
| hsa-miR-342-3p | 48.67 | 104.91 | 61.64 | 132.67 | 76.17 | 83.61 | 220.15 | 168.2 | 171.33 | 211.24 |
| hsa-miR-548ai | 104.54 | 174.84 | 123.28 | 157.95 | 157.42 | 154.67 | 94.35 | 101.35 | 119.93 | 87.79 |
| hsa-miR-644a | 52.27 | 131.13 | 71.92 | 214.81 | 228.52 | 125.41 | 139.78 | 99.19 | 107.08 | 104.25 |
| hsa-miR-663a | 37.85 | 131.13 | 76.02 | 164.26 | 147.27 | 171.39 | 139.78 | 114.29 | 134.92 | 156.37 |
| hsa-miR-656 | 66.69 | 52.45 | 76.02 | 164.26 | 198.05 | 108.69 | 153.75 | 122.91 | 154.2 | 142.66 |
| hsa-miR-548k | 45.06 | 139.87 | 51.37 | 132.67 | 111.72 | 133.77 | 185.2 | 140.16 | 139.21 | 156.37 |
| hsa-miR-627 | 54.07 | 61.19 | 61.64 | 208.49 | 157.42 | 137.95 | 139.78 | 138.01 | 147.77 | 120.71 |
| hsa-miR-2682-5p | 115.36 | 61.19 | 137.67 | 75.81 | 81.25 | 150.49 | 115.32 | 146.63 | 156.34 | 186.55 |
| hsa-miR-504 | 120.77 | 166.1 | 139.72 | 170.58 | 203.13 | 125.41 | 62.9 | 86.26 | 68.53 | 76.81 |
| hsa-miR-95 | 66.69 | 113.65 | 53.42 | 138.99 | 243.75 | 129.59 | 139.78 | 112.13 | 104.94 | 115.22 |
| hsa-miR-1202 | 104.54 | 113.65 | 108.9 | 164.26 | 187.89 | 158.85 | 97.84 | 101.35 | 89.95 | 85.04 |
| hsa-miR-1909-3p | 54.07 | 131.13 | 59.59 | 132.67 | 192.97 | 133.77 | 174.72 | 86.26 | 128.5 | 117.97 |
| hsa-miR-3200-3p | 79.31 | 139.87 | 94.52 | 259.03 | 350.39 | 154.67 | 24.46 | 36.66 | 27.84 | 43.89 |
| hsa-miR-1277-3p | 46.86 | 104.91 | 57.53 | 157.95 | 142.19 | 112.87 | 174.72 | 150.95 | 109.22 | 145.4 |
| hsa-miR-450b-5p | 55.88 | 78.68 | 78.08 | 151.63 | 111.72 | 79.43 | 174.72 | 200.54 | 119.93 | 148.14 |
| hsa-miR-3190-5p | 70.3 | 201.07 | 73.97 | 233.76 | 167.58 | 125.41 | 101.34 | 58.22 | 51.4 | 115.22 |
| hsa-miR-10a-5p | 106.35 | 87.42 | 129.45 | 75.81 | 101.56 | 133.77 | 104.83 | 150.95 | 137.06 | 164.6 |
| hsa-miR-384 | 43.26 | 61.19 | 51.37 | 138.99 | 187.89 | 96.15 | 164.24 | 120.76 | 124.22 | 203.01 |
| hsa-miR-191-5p | 77.51 | 157.36 | 82.19 | 157.95 | 172.66 | 91.97 | 111.82 | 150.95 | 81.38 | 106.99 |
| hsa-miR-499a-3p | 61.28 | 61.19 | 47.26 | 113.72 | 121.88 | 96.15 | 181.71 | 200.54 | 145.63 | 159.12 |
| hsa-miR-301b | 55.88 | 139.87 | 63.7 | 170.58 | 182.81 | 179.75 | 129.29 | 94.88 | 74.96 | 96.02 |
| hsa-miR-519b-5p+hsa-miR-519c-5p | 55.88 | 148.62 | 57.53 | 145.31 | 167.58 | 117.05 | 167.73 | 109.98 | 117.79 | 98.76 |
| hsa-miR-548z | 68.49 | 139.87 | 96.57 | 170.58 | 162.5 | 146.31 | 94.35 | 107.82 | 72.82 | 126.2 |
| hsa-miR-515-5p | 45.06 | 43.71 | 82.19 | 164.26 | 126.95 | 54.34 | 167.73 | 185.45 | 149.91 | 159.12 |
| hsa-miR-654-3p | 48.67 | 96.16 | 47.26 | 69.5 | 111.72 | 96.15 | 202.68 | 159.57 | 147.77 | 197.52 |
| hsa-miR-4455 | 70.3 | 69.94 | 96.57 | 157.95 | 147.27 | 121.23 | 136.28 | 135.85 | 102.8 | 137.17 |
| hsa-miR-598 | 46.86 | 104.91 | 73.97 | 113.72 | 91.41 | 112.87 | 164.24 | 150.95 | 111.37 | 203.01 |
| hsa-miR-3605-5p | 61.28 | 122.39 | 65.75 | 240.08 | 192.97 | 146.31 | 101.34 | 84.1 | 83.52 | 74.07 |
| hsa-miR-518c-3p | 63.09 | 69.94 | 71.92 | 145.31 | 172.66 | 104.51 | 157.25 | 97.04 | 147.77 | 139.91 |
| hsa-miR-133b | 46.86 | 104.91 | 76.02 | 145.31 | 167.58 | 112.87 | 139.78 | 99.19 | 145.63 | 128.94 |
| hsa-miR-548d-3p | 75.7 | 69.94 | 100.68 | 132.67 | 91.41 | 100.33 | 153.75 | 161.73 | 128.5 | 150.89 |
| hsa-miR-566 | 54.07 | 113.65 | 65.75 | 138.99 | 147.27 | 108.69 | 139.78 | 116.44 | 117.79 | 150.89 |
| hsa-miR-761 | 64.89 | 78.68 | 65.75 | 88.45 | 147.27 | 150.49 | 132.79 | 138.01 | 109.22 | 175.58 |
| hsa-miR-330-3p | 55.88 | 174.84 | 45.2 | 157.95 | 238.67 | 154.67 | 76.88 | 88.41 | 83.52 | 65.84 |
| hsa-miR-4458 | 70.3 | 69.94 | 69.86 | 132.67 | 192.97 | 142.13 | 139.78 | 120.76 | 96.37 | 101.5 |
| hsa-miR-520h | 142.4 | 96.16 | 141.78 | 107.4 | 106.64 | 125.41 | 83.87 | 107.82 | 94.23 | 128.94 |
| hsa-miR-516a-5p | 27.04 | 131.13 | 47.26 | 88.45 | 106.64 | 87.79 | 167.73 | 140.16 | 162.76 | 175.58 |
| hsa-miR-888-5p | 131.58 | 131.13 | 182.87 | 56.86 | 71.09 | 66.88 | 108.33 | 146.63 | 122.07 | 115.22 |
| hsa-miR-370 | 45.06 | 104.91 | 53.42 | 75.81 | 116.8 | 87.79 | 209.66 | 144.48 | 132.78 | 161.86 |
| hsa-miR-1226-3p | 54.07 | 166.1 | 65.75 | 126.36 | 137.11 | 91.97 | 157.25 | 114.29 | 111.37 | 106.99 |
| hsa-miR-193b-3p | 59.48 | 78.68 | 69.86 | 195.85 | 162.5 | 112.87 | 111.82 | 92.72 | 128.5 | 117.97 |
| hsa-miR-1252 | 50.47 | 61.19 | 73.97 | 195.85 | 182.81 | 117.05 | 139.78 | 103.51 | 85.67 | 117.97 |
| hsa-miR-412 | 54.07 | 61.19 | 34.93 | 221.12 | 147.27 | 154.67 | 139.78 | 109.98 | 94.23 | 106.99 |
| hsa-miR-759 | 54.07 | 87.42 | 57.53 | 221.12 | 233.6 | 96.15 | 94.35 | 99.19 | 89.95 | 87.79 |
| hsa-miR-30e-5p | 100.94 | 78.68 | 69.86 | 120.04 | 162.5 | 146.31 | 94.35 | 112.13 | 126.36 | 109.74 |
| hsa-miR-1908 | 50.47 | 96.16 | 63.7 | 107.4 | 137.11 | 175.57 | 174.72 | 112.13 | 87.81 | 112.48 |
| hsa-miR-1273d | 61.28 | 96.16 | 82.19 | 151.63 | 198.05 | 150.49 | 125.8 | 94.88 | 77.1 | 79.56 |
| hsa-miR-449a | 46.86 | 78.68 | 90.41 | 145.31 | 274.22 | 108.69 | 101.34 | 79.79 | 87.81 | 98.76 |
| hsa-miR-338-3p | 95.53 | 61.19 | 90.41 | 145.31 | 147.27 | 100.33 | 129.29 | 127.23 | 128.5 | 85.04 |
| hsa-let-7g-5p | 68.49 | 104.91 | 47.26 | 120.04 | 172.66 | 79.43 | 150.26 | 150.95 | 81.38 | 134.43 |
| hsa-miR-590-5p | 61.28 | 69.94 | 65.75 | 157.95 | 121.88 | 150.49 | 122.3 | 144.48 | 115.65 | 98.76 |
| hsa-miR-484 | 46.86 | 166.1 | 65.75 | 157.95 | 177.74 | 125.41 | 115.32 | 71.16 | 79.24 | 98.76 |
| hsa-miR-18a-5p | 39.65 | 122.39 | 49.31 | 145.31 | 167.58 | 133.77 | 118.81 | 97.04 | 102.8 | 126.2 |
| hsa-miR-2277-3p | 57.68 | 113.65 | 73.97 | 126.36 | 142.19 | 104.51 | 139.78 | 131.54 | 96.37 | 112.48 |
| hsa-miR-33a-5p | 48.67 | 87.42 | 45.2 | 157.95 | 147.27 | 91.97 | 115.32 | 133.7 | 122.07 | 148.14 |
| hsa-miR-1 | 68.49 | 52.45 | 57.53 | 151.63 | 167.58 | 167.21 | 108.33 | 122.91 | 64.25 | 134.43 |
| hsa-miR-770-5p | 55.88 | 78.68 | 63.7 | 107.4 | 162.5 | 154.67 | 132.79 | 125.07 | 87.81 | 123.45 |
| hsa-miR-2276 | 46.86 | 122.39 | 86.3 | 113.72 | 137.11 | 133.77 | 122.3 | 122.91 | 87.81 | 117.97 |
| hsa-miR-1323 | 61.28 | 78.68 | 71.92 | 120.04 | 126.95 | 91.97 | 167.73 | 127.23 | 124.22 | 120.71 |
| hsa-miR-556-5p | 102.74 | 183.58 | 113.01 | 195.85 | 198.05 | 108.69 | 45.43 | 43.13 | 64.25 | 32.92 |
| hsa-miR-216b | 120.77 | 104.91 | 119.17 | 94.77 | 86.33 | 87.79 | 87.36 | 129.38 | 107.08 | 148.14 |
| hsa-miR-212-3p | 68.49 | 69.94 | 63.7 | 145.31 | 162.5 | 137.95 | 108.33 | 114.29 | 100.66 | 112.48 |
| hsa-miR-1273a | 55.88 | 139.87 | 45.2 | 214.81 | 208.2 | 121.23 | 90.85 | 43.13 | 55.68 | 106.99 |
| hsa-miR-16-5p | 102.74 | 69.94 | 141.78 | 120.04 | 91.41 | 96.15 | 101.34 | 159.57 | 94.23 | 104.25 |
| hsa-miR-2116-5p | 99.14 | 139.87 | 102.74 | 94.77 | 96.49 | 58.52 | 115.32 | 118.6 | 119.93 | 134.43 |
| hsa-miR-382-5p | 37.85 | 96.16 | 80.13 | 195.85 | 233.6 | 117.05 | 76.88 | 69 | 68.53 | 104.25 |
| hsa-miR-548c-5p | 108.15 | 174.84 | 199.31 | 75.81 | 50.78 | 158.85 | 73.38 | 75.47 | 77.1 | 82.3 |
| hsa-miR-302e | 54.07 | 104.91 | 61.64 | 157.95 | 91.41 | 125.41 | 115.32 | 144.48 | 85.67 | 134.43 |
| hsa-miR-4425 | 52.27 | 78.68 | 57.53 | 132.67 | 116.8 | 108.69 | 160.74 | 122.91 | 122.07 | 120.71 |
| hsa-miR-891a | 82.91 | 122.39 | 82.19 | 164.26 | 137.11 | 104.51 | 94.35 | 92.72 | 87.81 | 104.25 |
| hsa-miR-4741 | 32.44 | 131.13 | 55.48 | 151.63 | 198.05 | 87.79 | 104.83 | 92.72 | 107.08 | 109.74 |
| hsa-miR-655 | 95.53 | 183.58 | 215.75 | 75.81 | 121.88 | 117.05 | 52.42 | 77.63 | 53.54 | 76.81 |
| hsa-miR-892b | 63.09 | 87.42 | 67.81 | 176.9 | 187.89 | 91.97 | 122.3 | 81.94 | 83.52 | 106.99 |
| hsa-miR-499a-5p | 95.53 | 131.13 | 96.57 | 164.26 | 116.8 | 121.23 | 80.37 | 49.6 | 72.82 | 139.91 |
| hsa-miR-548aa | 106.35 | 166.1 | 154.1 | 107.4 | 76.17 | 129.59 | 66.39 | 62.54 | 102.8 | 93.27 |
| hsa-miR-647 | 50.47 | 87.42 | 84.24 | 170.58 | 152.34 | 87.79 | 143.27 | 92.72 | 74.96 | 120.71 |
| hsa-miR-1238 | 46.86 | 96.16 | 39.04 | 164.26 | 192.97 | 100.33 | 108.33 | 116.44 | 79.24 | 117.97 |
| hsa-miR-511 | 52.27 | 113.65 | 61.64 | 164.26 | 203.13 | 112.87 | 97.84 | 92.72 | 98.52 | 63.1 |
| hsa-miR-544a | 68.49 | 104.91 | 71.92 | 170.58 | 111.72 | 79.43 | 122.3 | 127.23 | 96.37 | 106.99 |
| hsa-miR-432-5p | 61.28 | 104.91 | 71.92 | 126.36 | 126.95 | 75.24 | 174.72 | 92.72 | 109.22 | 115.22 |
| hsa-miR-28-5p | 36.05 | 131.13 | 36.98 | 145.31 | 223.44 | 129.59 | 111.82 | 64.69 | 77.1 | 101.5 |
| hsa-miR-200a-3p | 72.1 | 104.91 | 90.41 | 126.36 | 116.8 | 108.69 | 87.36 | 163.88 | 79.24 | 104.25 |
| hsa-miR-124-3p | 79.31 | 96.16 | 90.41 | 195.85 | 192.97 | 142.13 | 83.87 | 51.75 | 47.12 | 71.33 |
| hsa-miR-449c-5p | 50.47 | 69.94 | 53.42 | 101.09 | 121.88 | 121.23 | 171.23 | 131.54 | 122.07 | 101.5 |
| hsa-miR-3690 | 36.05 | 131.13 | 80.13 | 126.36 | 167.58 | 129.59 | 115.32 | 62.54 | 77.1 | 117.97 |
| hsa-miR-516a-3p | 55.88 | 157.36 | 78.08 | 138.99 | 126.95 | 91.97 | 115.32 | 107.82 | 74.96 | 96.02 |
| hsa-miR-580 | 45.06 | 61.19 | 43.15 | 75.81 | 106.64 | 83.61 | 160.74 | 163.88 | 107.08 | 186.55 |
| hsa-miR-337-3p | 88.32 | 148.62 | 78.08 | 120.04 | 106.64 | 91.97 | 108.33 | 103.51 | 92.09 | 96.02 |
| hsa-miR-938 | 27.04 | 96.16 | 51.37 | 176.9 | 106.64 | 91.97 | 171.23 | 112.13 | 74.96 | 123.45 |
| hsa-miR-1538 | 73.9 | 139.87 | 104.79 | 151.63 | 132.03 | 142.13 | 97.84 | 51.75 | 51.4 | 79.56 |
| hsa-miR-525-5p | 43.26 | 183.58 | 55.48 | 157.95 | 167.58 | 104.51 | 80.37 | 58.22 | 74.96 | 96.02 |
| hsa-miR-1298 | 55.88 | 131.13 | 73.97 | 157.95 | 152.34 | 163.03 | 66.39 | 73.32 | 51.4 | 96.02 |
| hsa-miR-152 | 52.27 | 122.39 | 69.86 | 138.99 | 162.5 | 129.59 | 94.35 | 81.94 | 72.82 | 96.02 |
| hsa-miR-4461 | 46.86 | 104.91 | 63.7 | 113.72 | 116.8 | 163.03 | 118.81 | 92.72 | 85.67 | 106.99 |
| hsa-miR-658 | 43.26 | 69.94 | 59.59 | 126.36 | 147.27 | 108.69 | 153.75 | 84.1 | 89.95 | 128.94 |
| hsa-miR-23c | 43.26 | 96.16 | 65.75 | 113.72 | 132.03 | 91.97 | 139.78 | 99.19 | 122.07 | 106.99 |
| hsa-miR-1185-5p | 37.85 | 78.68 | 51.37 | 69.5 | 66.02 | 79.43 | 209.66 | 157.42 | 124.22 | 134.43 |
| hsa-miR-494 | 93.73 | 87.42 | 63.7 | 69.5 | 126.95 | 87.79 | 115.32 | 122.91 | 117.79 | 123.45 |
| hsa-miR-140-5p | 63.09 | 104.91 | 45.2 | 132.67 | 187.89 | 71.06 | 132.79 | 90.57 | 79.24 | 96.02 |
| hsa-miR-3175 | 45.06 | 87.42 | 36.98 | 189.54 | 121.88 | 104.51 | 122.3 | 97.04 | 96.37 | 101.5 |
| hsa-miR-22-3p | 28.84 | 122.39 | 55.48 | 145.31 | 162.5 | 87.79 | 136.28 | 71.16 | 79.24 | 112.48 |
| hsa-miR-23a-3p | 48.67 | 69.94 | 115.06 | 151.63 | 147.27 | 91.97 | 111.82 | 138.01 | 64.25 | 60.35 |
| hsa-miR-301a-3p | 59.48 | 122.39 | 55.48 | 170.58 | 137.11 | 133.77 | 80.37 | 97.04 | 51.4 | 85.04 |
| hsa-let-7d-5p | 68.49 | 104.91 | 76.02 | 88.45 | 101.56 | 87.79 | 122.3 | 125.07 | 104.94 | 112.48 |
| hsa-miR-876-3p | 59.48 | 69.94 | 51.37 | 126.36 | 121.88 | 108.69 | 125.8 | 122.91 | 111.37 | 93.27 |
| hsa-miR-604 | 55.88 | 96.16 | 47.26 | 120.04 | 172.66 | 112.87 | 125.8 | 77.63 | 92.09 | 87.79 |
| hsa-miR-532-5p | 59.48 | 104.91 | 59.59 | 145.31 | 162.5 | 87.79 | 108.33 | 101.35 | 81.38 | 76.81 |
| hsa-miR-330-5p | 52.27 | 131.13 | 43.15 | 170.58 | 96.49 | 87.79 | 125.8 | 62.54 | 107.08 | 109.74 |
| hsa-miR-605 | 46.86 | 139.87 | 61.64 | 88.45 | 132.03 | 137.95 | 111.82 | 86.26 | 79.24 | 101.5 |
| hsa-miR-383 | 30.64 | 131.13 | 65.75 | 189.54 | 132.03 | 117.05 | 90.85 | 64.69 | 81.38 | 82.3 |
| hsa-miR-1324 | 86.52 | 87.42 | 61.64 | 183.22 | 142.19 | 104.51 | 87.36 | 71.16 | 70.67 | 90.53 |
| hsa-miR-99b-5p | 36.05 | 113.65 | 51.37 | 113.72 | 147.27 | 117.05 | 125.8 | 94.88 | 77.1 | 106.99 |
| hsa-miR-548n | 37.85 | 96.16 | 36.98 | 170.58 | 203.13 | 75.24 | 87.36 | 73.32 | 109.22 | 93.27 |
| hsa-miR-1257 | 48.67 | 122.39 | 78.08 | 120.04 | 106.64 | 75.24 | 136.28 | 75.47 | 102.8 | 115.22 |
| hsa-miR-550b-3p | 90.12 | 139.87 | 123.28 | 56.86 | 55.86 | 121.23 | 69.89 | 140.16 | 92.09 | 87.79 |
| hsa-miR-574-3p | 32.44 | 78.68 | 65.75 | 107.4 | 187.89 | 117.05 | 115.32 | 97.04 | 81.38 | 93.27 |
| hsa-miR-4454 | 64.89 | 78.68 | 51.37 | 63.18 | 111.72 | 91.97 | 94.35 | 191.92 | 98.52 | 128.94 |
| hsa-miR-1973 | 34.25 | 113.65 | 69.86 | 221.12 | 116.8 | 121.23 | 73.38 | 79.79 | 72.82 | 71.33 |
| hsa-miR-411-5p | 91.93 | 113.65 | 98.63 | 63.18 | 132.03 | 142.13 | 87.36 | 92.72 | 70.67 | 79.56 |
| hsa-miR-7-5p | 55.88 | 104.91 | 65.75 | 170.58 | 111.72 | 71.06 | 101.34 | 101.35 | 70.67 | 115.22 |
| hsa-miR-450a-5p | 52.27 | 52.45 | 67.81 | 94.77 | 91.41 | 108.69 | 139.78 | 138.01 | 109.22 | 112.48 |
| hsa-miR-659-3p | 39.65 | 61.19 | 43.15 | 82.13 | 96.49 | 87.79 | 139.78 | 120.76 | 132.78 | 161.86 |
| hsa-miR-1268a | 52.27 | 122.39 | 51.37 | 233.76 | 162.5 | 112.87 | 66.39 | 62.54 | 32.12 | 68.58 |
| hsa-miR-3196 | 54.07 | 78.68 | 69.86 | 157.95 | 121.88 | 96.15 | 83.87 | 79.79 | 102.8 | 117.97 |
| hsa-miR-1245b-5p | 43.26 | 69.94 | 49.31 | 214.81 | 126.95 | 87.79 | 104.83 | 73.32 | 87.81 | 104.25 |
| hsa-miR-1275 | 84.72 | 43.71 | 63.7 | 145.31 | 182.81 | 104.51 | 73.38 | 66.85 | 87.81 | 106.99 |
| hsa-miR-890 | 55.88 | 69.94 | 57.53 | 113.72 | 137.11 | 75.24 | 125.8 | 112.13 | 104.94 | 106.99 |
| hsa-miR-335-5p | 50.47 | 61.19 | 47.26 | 145.31 | 147.27 | 79.43 | 122.3 | 101.35 | 94.23 | 109.74 |
| hsa-miR-4647 | 43.26 | 96.16 | 30.82 | 164.26 | 152.34 | 121.23 | 115.32 | 79.79 | 70.67 | 82.3 |
| hsa-miR-29c-3p | 55.88 | 87.42 | 57.53 | 164.26 | 137.11 | 87.79 | 118.81 | 103.51 | 68.53 | 74.07 |
| hsa-let-7c | 50.47 | 78.68 | 67.81 | 164.26 | 116.8 | 83.61 | 111.82 | 99.19 | 74.96 | 104.25 |
| hsa-miR-922 | 45.06 | 69.94 | 65.75 | 157.95 | 137.11 | 87.79 | 111.82 | 94.88 | 77.1 | 98.76 |
| hsa-miR-93-5p | 59.48 | 96.16 | 65.75 | 120.04 | 126.95 | 146.31 | 80.37 | 105.66 | 74.96 | 65.84 |
| hsa-let-7b-5p | 54.07 | 87.42 | 88.35 | 145.31 | 162.5 | 100.33 | 83.87 | 64.69 | 79.24 | 74.07 |
| hsa-miR-302b-3p | 43.26 | 34.97 | 51.37 | 101.09 | 142.19 | 125.41 | 111.82 | 107.82 | 81.38 | 137.17 |
| hsa-miR-518b | 43.26 | 78.68 | 41.09 | 101.09 | 106.64 | 58.52 | 160.74 | 105.66 | 74.96 | 164.6 |
| hsa-miR-935 | 54.07 | 131.13 | 86.3 | 189.54 | 126.95 | 158.85 | 52.42 | 51.75 | 42.83 | 41.15 |
| hsa-miR-1225-3p | 50.47 | 166.1 | 53.42 | 75.81 | 228.52 | 150.49 | 62.9 | 47.44 | 42.83 | 54.87 |
| hsa-miR-590-3p | 46.86 | 104.91 | 71.92 | 214.81 | 126.95 | 117.05 | 94.35 | 73.32 | 40.69 | 41.15 |
| hsa-miR-4508 | 43.26 | 43.71 | 49.31 | 44.22 | 55.86 | 62.7 | 178.21 | 103.51 | 156.34 | 194.78 |
| hsa-miR-325 | 50.47 | 104.91 | 88.35 | 31.59 | 96.49 | 125.41 | 139.78 | 92.72 | 83.52 | 117.97 |
| hsa-miR-891b | 48.67 | 52.45 | 80.13 | 157.95 | 101.56 | 87.79 | 90.85 | 99.19 | 94.23 | 117.97 |
| hsa-miR-1234 | 79.31 | 122.39 | 127.39 | 63.18 | 86.33 | 129.59 | 101.34 | 77.63 | 70.67 | 71.33 |
| hsa-miR-485-5p | 39.65 | 69.94 | 47.26 | 151.63 | 132.03 | 91.97 | 118.81 | 94.88 | 94.23 | 87.79 |
| hsa-miR-2114-5p | 68.49 | 78.68 | 65.75 | 189.54 | 162.5 | 104.51 | 90.85 | 56.07 | 53.54 | 57.61 |
| hsa-miR-153 | 43.26 | 131.13 | 53.42 | 101.09 | 106.64 | 96.15 | 94.35 | 86.26 | 89.95 | 123.45 |
| hsa-miR-526a+hsa-miR-520c-5p+hsa-miR-518d-5p | 43.26 | 52.45 | 59.59 | 107.4 | 132.03 | 91.97 | 136.28 | 116.44 | 79.24 | 106.99 |
| hsa-miR-1825 | 45.06 | 113.65 | 59.59 | 145.31 | 111.72 | 75.24 | 118.81 | 88.41 | 68.53 | 98.76 |
| hsa-miR-141-3p | 48.67 | 78.68 | 96.57 | 132.67 | 126.95 | 66.88 | 87.36 | 140.16 | 49.26 | 96.02 |
| hsa-miR-329 | 48.67 | 78.68 | 69.86 | 120.04 | 157.42 | 108.69 | 80.37 | 71.16 | 85.67 | 101.5 |
| hsa-miR-548g-3p | 55.88 | 113.65 | 55.48 | 126.36 | 121.88 | 91.97 | 94.35 | 79.79 | 74.96 | 106.99 |
| hsa-miR-1200 | 55.88 | 87.42 | 32.88 | 132.67 | 121.88 | 71.06 | 129.29 | 109.98 | 92.09 | 87.79 |
| hsa-miR-568 | 16.22 | 61.19 | 36.98 | 44.22 | 76.17 | 41.8 | 216.65 | 125.07 | 145.63 | 156.37 |
| hsa-miR-139-3p | 54.07 | 96.16 | 67.81 | 145.31 | 147.27 | 79.43 | 108.33 | 64.69 | 55.68 | 98.76 |
| hsa-miR-26a-5p | 81.11 | 61.19 | 57.53 | 176.9 | 187.89 | 62.7 | 80.37 | 71.16 | 57.82 | 79.56 |
| hsa-miR-148b-3p | 52.27 | 34.97 | 98.63 | 157.95 | 116.8 | 71.06 | 101.34 | 107.82 | 70.67 | 101.5 |
| hsa-miR-513a-3p | 59.48 | 131.13 | 57.53 | 138.99 | 142.19 | 96.15 | 94.35 | 75.47 | 51.4 | 63.1 |
| hsa-miR-629-5p | 66.69 | 43.71 | 67.81 | 126.36 | 162.5 | 87.79 | 76.88 | 81.94 | 102.8 | 93.27 |
| hsa-miR-34c-5p | 39.65 | 69.94 | 47.26 | 75.81 | 76.17 | 108.69 | 108.33 | 204.86 | 79.24 | 98.76 |
| hsa-miR-613 | 48.67 | 113.65 | 51.37 | 132.67 | 157.42 | 104.51 | 104.83 | 56.07 | 81.38 | 57.61 |
| hsa-miR-769-3p | 48.67 | 69.94 | 67.81 | 113.72 | 198.05 | 87.79 | 115.32 | 51.75 | 53.54 | 101.5 |
| hsa-miR-769-5p | 59.48 | 61.19 | 63.7 | 107.4 | 101.56 | 87.79 | 115.32 | 116.44 | 89.95 | 104.25 |
| hsa-miR-941 | 41.46 | 113.65 | 24.66 | 126.36 | 116.8 | 75.24 | 108.33 | 105.66 | 117.79 | 76.81 |
| hsa-miR-606 | 54.07 | 61.19 | 39.04 | 63.18 | 40.63 | 50.16 | 195.69 | 127.23 | 130.64 | 142.66 |
| hsa-miR-151a-5p | 41.46 | 131.13 | 45.2 | 120.04 | 96.49 | 112.87 | 104.83 | 69 | 77.1 | 104.25 |
| hsa-let-7f-5p | 48.67 | 78.68 | 49.31 | 132.67 | 126.95 | 100.33 | 108.33 | 79.79 | 81.38 | 96.02 |
| hsa-miR-3182 | 37.85 | 69.94 | 53.42 | 126.36 | 111.72 | 125.41 | 94.35 | 94.88 | 94.23 | 90.53 |
| hsa-miR-762 | 34.25 | 104.91 | 71.92 | 132.67 | 126.95 | 66.88 | 104.83 | 73.32 | 72.82 | 109.74 |
| hsa-miR-503 | 43.26 | 69.94 | 34.93 | 145.31 | 81.25 | 117.05 | 136.28 | 86.26 | 79.24 | 101.5 |
| hsa-miR-635 | 34.25 | 87.42 | 26.71 | 120.04 | 96.49 | 108.69 | 108.33 | 107.82 | 89.95 | 115.22 |
| hsa-miR-367-3p | 59.48 | 96.16 | 63.7 | 107.4 | 147.27 | 79.43 | 76.88 | 88.41 | 79.24 | 96.02 |
| hsa-miR-1225-5p | 57.68 | 43.71 | 59.59 | 63.18 | 106.64 | 83.61 | 139.78 | 94.88 | 134.92 | 109.74 |
| hsa-miR-1270 | 81.11 | 131.13 | 121.23 | 132.67 | 132.03 | 91.97 | 41.93 | 45.28 | 53.54 | 60.35 |
| hsa-miR-1228-3p | 37.85 | 157.36 | 67.81 | 132.67 | 152.34 | 91.97 | 52.42 | 51.75 | 64.25 | 79.56 |
| hsa-miR-214-3p | 57.68 | 78.68 | 24.66 | 107.4 | 116.8 | 91.97 | 132.79 | 86.26 | 66.39 | 123.45 |
| hsa-miR-1912 | 45.06 | 104.91 | 63.7 | 195.85 | 147.27 | 146.31 | 80.37 | 34.5 | 23.56 | 43.89 |
| hsa-miR-519c-3p | 52.27 | 87.42 | 53.42 | 157.95 | 96.49 | 91.97 | 97.84 | 73.32 | 64.25 | 106.99 |
| hsa-miR-3147 | 34.25 | 96.16 | 55.48 | 138.99 | 198.05 | 91.97 | 111.82 | 45.28 | 59.97 | 49.38 |
| hsa-miR-410 | 39.65 | 78.68 | 32.88 | 164.26 | 137.11 | 112.87 | 115.32 | 73.32 | 57.82 | 68.58 |
| hsa-miR-106a-5p+hsa-miR-17-5p | 37.85 | 131.13 | 61.64 | 120.04 | 126.95 | 79.43 | 97.84 | 86.26 | 66.39 | 71.33 |
| hsa-miR-585 | 39.65 | 96.16 | 67.81 | 120.04 | 116.8 | 75.24 | 108.33 | 92.72 | 59.97 | 101.5 |
| hsa-miR-1197 | 32.44 | 104.91 | 59.59 | 88.45 | 111.72 | 91.97 | 90.85 | 79.79 | 104.94 | 112.48 |
| hsa-miR-513a-5p | 59.48 | 69.94 | 98.63 | 101.09 | 142.19 | 117.05 | 73.38 | 53.91 | 77.1 | 79.56 |
| hsa-miR-200c-3p | 39.65 | 61.19 | 69.86 | 69.5 | 76.17 | 83.61 | 139.78 | 129.38 | 98.52 | 104.25 |
| hsa-miR-1244 | 66.69 | 78.68 | 67.81 | 145.31 | 132.03 | 79.43 | 83.87 | 64.69 | 62.11 | 90.53 |
| hsa-miR-578 | 39.65 | 69.94 | 53.42 | 107.4 | 111.72 | 58.52 | 125.8 | 109.98 | 81.38 | 112.48 |
| hsa-miR-569 | 48.67 | 69.94 | 53.42 | 164.26 | 142.19 | 121.23 | 80.37 | 38.81 | 57.82 | 93.27 |
| hsa-miR-1299 | 34.25 | 69.94 | 53.42 | 138.99 | 137.11 | 75.24 | 73.38 | 73.32 | 96.37 | 115.22 |
| hsa-miR-548am-3p | 32.44 | 87.42 | 57.53 | 88.45 | 126.95 | 75.24 | 73.38 | 116.44 | 89.95 | 117.97 |
| hsa-miR-27a-3p | 30.64 | 52.45 | 34.93 | 164.26 | 172.66 | 100.33 | 101.34 | 62.54 | 66.39 | 79.56 |
| hsa-miR-28-3p | 57.68 | 61.19 | 71.92 | 94.77 | 111.72 | 79.43 | 108.33 | 120.76 | 70.67 | 87.79 |
| hsa-miR-638 | 57.68 | 34.97 | 69.86 | 120.04 | 91.41 | 62.7 | 157.25 | 73.32 | 89.95 | 106.99 |
| hsa-miR-548a-3p | 28.84 | 69.94 | 61.64 | 107.4 | 101.56 | 117.05 | 87.36 | 71.16 | 100.66 | 117.97 |
| hsa-miR-542-3p | 54.07 | 122.39 | 61.64 | 107.4 | 172.66 | 79.43 | 76.88 | 90.57 | 42.83 | 54.87 |
| hsa-miR-376b | 50.47 | 69.94 | 49.31 | 126.36 | 172.66 | 96.15 | 76.88 | 62.54 | 72.82 | 85.04 |
| hsa-miR-195-5p | 36.05 | 69.94 | 47.26 | 183.22 | 96.49 | 33.44 | 125.8 | 92.72 | 92.09 | 85.04 |
| hsa-miR-320a | 48.67 | 104.91 | 41.09 | 126.36 | 101.56 | 129.59 | 101.34 | 71.16 | 53.54 | 82.3 |
| hsa-miR-563 | 32.44 | 34.97 | 45.2 | 101.09 | 126.95 | 50.16 | 150.26 | 127.23 | 89.95 | 101.5 |
| hsa-miR-519e-3p | 39.65 | 52.45 | 67.81 | 101.09 | 101.56 | 83.61 | 108.33 | 103.51 | 100.66 | 98.76 |
| hsa-miR-423-3p | 64.89 | 96.16 | 67.81 | 101.09 | 91.41 | 91.97 | 97.84 | 66.85 | 89.95 | 87.79 |
| hsa-miR-320d | 36.05 | 78.68 | 49.31 | 101.09 | 50.78 | 50.16 | 174.72 | 103.51 | 126.36 | 85.04 |
| hsa-miR-142-3p | 196.47 | 78.68 | 90.41 | 88.45 | 45.7 | 58.52 | 90.85 | 73.32 | 68.53 | 63.1 |
| hsa-miR-424-5p | 46.86 | 69.94 | 30.82 | 56.86 | 96.49 | 96.15 | 164.24 | 101.35 | 92.09 | 98.76 |
| hsa-miR-641 | 57.68 | 52.45 | 61.64 | 101.09 | 142.19 | 66.88 | 94.35 | 77.63 | 94.23 | 104.25 |
| hsa-miR-205-5p | 52.27 | 34.97 | 59.59 | 113.72 | 81.25 | 108.69 | 87.36 | 161.73 | 87.81 | 63.1 |
| hsa-miR-151a-3p | 52.27 | 104.91 | 45.2 | 120.04 | 137.11 | 125.41 | 69.89 | 51.75 | 59.97 | 82.3 |
| hsa-miR-1178 | 41.46 | 104.91 | 41.09 | 94.77 | 152.34 | 87.79 | 94.35 | 62.54 | 81.38 | 87.79 |
| hsa-let-7a-5p | 91.93 | 96.16 | 69.86 | 113.72 | 86.33 | 75.24 | 80.37 | 86.26 | 72.82 | 74.07 |
| hsa-miR-512-5p | 55.88 | 87.42 | 90.41 | 113.72 | 76.17 | 91.97 | 83.87 | 101.35 | 66.39 | 79.56 |
| hsa-miR-1255a | 48.67 | 96.16 | 71.92 | 145.31 | 111.72 | 121.23 | 55.91 | 49.6 | 66.39 | 79.56 |
| hsa-miR-519b-3p | 34.25 | 131.13 | 65.75 | 151.63 | 157.42 | 129.59 | 59.4 | 40.97 | 44.97 | 30.18 |
| hsa-miR-622 | 43.26 | 78.68 | 51.37 | 113.72 | 91.41 | 91.97 | 125.8 | 86.26 | 79.24 | 82.3 |
| hsa-miR-515-3p | 59.48 | 104.91 | 47.26 | 132.67 | 96.49 | 79.43 | 94.35 | 86.26 | 64.25 | 76.81 |
| hsa-miR-409-5p | 36.05 | 87.42 | 59.59 | 107.4 | 126.95 | 96.15 | 80.37 | 103.51 | 49.26 | 93.27 |
| hsa-miR-337-5p | 37.85 | 104.91 | 80.13 | 120.04 | 101.56 | 96.15 | 101.34 | 81.94 | 55.68 | 60.35 |
| hsa-miR-1322 | 39.65 | 78.68 | 53.42 | 69.5 | 106.64 | 137.95 | 104.83 | 84.1 | 66.39 | 98.76 |
| hsa-miR-939 | 43.26 | 78.68 | 51.37 | 157.95 | 126.95 | 45.98 | 104.83 | 66.85 | 66.39 | 96.02 |
| hsa-miR-1193 | 48.67 | 78.68 | 63.7 | 113.72 | 132.03 | 79.43 | 111.82 | 69 | 70.67 | 68.58 |
| hsa-miR-208a | 46.86 | 61.19 | 73.97 | 82.13 | 86.33 | 62.7 | 94.35 | 125.07 | 104.94 | 98.76 |
| hsa-miR-105-5p | 72.1 | 104.91 | 26.71 | 113.72 | 132.03 | 91.97 | 80.37 | 81.94 | 70.67 | 60.35 |
| hsa-miR-302f | 64.89 | 52.45 | 45.2 | 113.72 | 101.56 | 125.41 | 87.36 | 81.94 | 57.82 | 104.25 |
| hsa-miR-96-5p | 45.06 | 69.94 | 53.42 | 101.09 | 111.72 | 100.33 | 118.81 | 62.54 | 77.1 | 93.27 |
| hsa-miR-29a-3p | 48.67 | 61.19 | 39.04 | 101.09 | 91.41 | 108.69 | 104.83 | 90.57 | 83.52 | 104.25 |
| hsa-miR-9-5p | 68.49 | 69.94 | 98.63 | 63.18 | 121.88 | 91.97 | 90.85 | 73.32 | 77.1 | 76.81 |
| hsa-miR-621 | 30.64 | 131.13 | 51.37 | 138.99 | 96.49 | 83.61 | 87.36 | 56.07 | 59.97 | 96.02 |
| hsa-miR-1539 | 48.67 | 87.42 | 51.37 | 170.58 | 116.8 | 66.88 | 80.37 | 60.38 | 68.53 | 79.56 |
| hsa-miR-1281 | 43.26 | 78.68 | 43.15 | 138.99 | 71.09 | 45.98 | 122.3 | 64.69 | 107.08 | 115.22 |
| hsa-miR-1231 | 23.43 | 96.16 | 28.77 | 82.13 | 111.72 | 71.06 | 150.26 | 101.35 | 74.96 | 90.53 |
| hsa-miR-1471 | 34.25 | 87.42 | 51.37 | 164.26 | 152.34 | 96.15 | 66.39 | 60.38 | 51.4 | 63.1 |
| hsa-miR-380-3p | 34.25 | 43.71 | 53.42 | 157.95 | 157.42 | 137.95 | 83.87 | 49.6 | 62.11 | 46.64 |
| hsa-miR-4532 | 32.44 | 52.45 | 45.2 | 145.31 | 126.95 | 87.79 | 111.82 | 84.1 | 68.53 | 71.33 |
| hsa-miR-129-2-3p | 48.67 | 96.16 | 36.98 | 107.4 | 121.88 | 79.43 | 125.8 | 60.38 | 70.67 | 76.81 |
| hsa-miR-1208 | 27.04 | 87.42 | 39.04 | 107.4 | 86.33 | 71.06 | 125.8 | 81.94 | 85.67 | 109.74 |
| hsa-miR-548t-5p | 32.44 | 69.94 | 49.31 | 101.09 | 147.27 | 62.7 | 129.29 | 38.81 | 89.95 | 98.76 |
| hsa-miR-3180 | 43.26 | 69.94 | 32.88 | 145.31 | 152.34 | 83.61 | 83.87 | 62.54 | 62.11 | 82.3 |
| hsa-miR-210 | 27.04 | 96.16 | 59.59 | 94.77 | 137.11 | 104.51 | 80.37 | 62.54 | 59.97 | 96.02 |
| hsa-miR-2278 | 39.65 | 78.68 | 43.15 | 94.77 | 147.27 | 112.87 | 104.83 | 47.44 | 64.25 | 85.04 |
| hsa-miR-626 | 32.44 | 52.45 | 59.59 | 75.81 | 116.8 | 75.24 | 90.85 | 94.88 | 100.66 | 117.97 |
| hsa-miR-3168 | 57.68 | 69.94 | 55.48 | 75.81 | 106.64 | 71.06 | 94.35 | 101.35 | 59.97 | 123.45 |
| hsa-miR-302a-3p | 30.64 | 87.42 | 53.42 | 101.09 | 76.17 | 121.23 | 111.82 | 66.85 | 70.67 | 96.02 |
| hsa-miR-519d | 61.28 | 69.94 | 137.67 | 50.54 | 30.47 | 112.87 | 66.39 | 92.72 | 102.8 | 90.53 |
| hsa-miR-575 | 70.3 | 78.68 | 39.04 | 31.59 | 76.17 | 54.34 | 48.92 | 301.89 | 72.82 | 41.15 |
| hsa-miR-3131 | 36.05 | 113.65 | 39.04 | 94.77 | 152.34 | 75.24 | 101.34 | 71.16 | 72.82 | 57.61 |
| hsa-miR-1279 | 36.05 | 34.97 | 67.81 | 101.09 | 157.42 | 96.15 | 90.85 | 77.63 | 66.39 | 85.04 |
| hsa-miR-767-3p | 46.86 | 61.19 | 45.2 | 132.67 | 152.34 | 87.79 | 83.87 | 77.63 | 59.97 | 65.84 |
| hsa-miR-596 | 45.06 | 87.42 | 59.59 | 113.72 | 121.88 | 87.79 | 94.35 | 73.32 | 55.68 | 74.07 |
| hsa-miR-1470 | 16.22 | 122.39 | 34.93 | 138.99 | 106.64 | 104.51 | 90.85 | 47.44 | 59.97 | 87.79 |
| hsa-miR-1272 | 54.07 | 78.68 | 86.3 | 75.81 | 81.25 | 71.06 | 90.85 | 69 | 89.95 | 112.48 |
| hsa-miR-517a-3p | 48.67 | 87.42 | 49.31 | 82.13 | 86.33 | 108.69 | 87.36 | 92.72 | 70.67 | 96.02 |
| hsa-miR-15a-5p | 48.67 | 78.68 | 47.26 | 145.31 | 116.8 | 79.43 | 97.84 | 90.57 | 47.12 | 57.61 |
| hsa-miR-520a-5p | 37.85 | 78.68 | 47.26 | 132.67 | 121.88 | 83.61 | 83.87 | 62.54 | 74.96 | 85.04 |
| hsa-miR-520d-5p+hsa-miR-518a-5p+hsa-miR-527 | 79.31 | 52.45 | 123.28 | 88.45 | 106.64 | 96.15 | 76.88 | 58.22 | 49.26 | 76.81 |
| hsa-miR-487a | 43.26 | 87.42 | 51.37 | 126.36 | 116.8 | 75.24 | 83.87 | 99.19 | 47.12 | 76.81 |
| hsa-miR-548c-3p | 34.25 | 104.91 | 59.59 | 145.31 | 121.88 | 91.97 | 59.4 | 60.38 | 55.68 | 74.07 |
| hsa-let-7i-5p | 39.65 | 69.94 | 63.7 | 82.13 | 106.64 | 41.8 | 122.3 | 86.26 | 100.66 | 93.27 |
| hsa-miR-764 | 41.46 | 78.68 | 43.15 | 170.58 | 116.8 | 66.88 | 104.83 | 40.97 | 53.54 | 87.79 |
| hsa-miR-3136-5p | 61.28 | 78.68 | 55.48 | 94.77 | 111.72 | 75.24 | 90.85 | 88.41 | 47.12 | 96.02 |
| hsa-miR-193a-3p | 43.26 | 61.19 | 36.98 | 157.95 | 126.95 | 125.41 | 73.38 | 60.38 | 53.54 | 60.35 |
| hsa-miR-614 | 30.64 | 78.68 | 39.04 | 107.4 | 106.64 | 75.24 | 115.32 | 79.79 | 62.11 | 104.25 |
| hsa-miR-339-3p | 34.25 | 87.42 | 43.15 | 107.4 | 91.41 | 112.87 | 111.82 | 81.94 | 59.97 | 68.58 |
| hsa-miR-671-5p | 32.44 | 78.68 | 39.04 | 107.4 | 126.95 | 112.87 | 80.37 | 75.47 | 62.11 | 82.3 |
| hsa-miR-196b-5p | 52.27 | 52.45 | 43.15 | 132.67 | 126.95 | 62.7 | 101.34 | 77.63 | 68.53 | 79.56 |
| hsa-miR-548aj-3p | 25.23 | 17.48 | 55.48 | 63.18 | 55.86 | 87.79 | 139.78 | 112.13 | 124.22 | 112.48 |
| hsa-miR-422a | 41.46 | 69.94 | 41.09 | 101.09 | 116.8 | 112.87 | 97.84 | 66.85 | 47.12 | 96.02 |
| hsa-miR-26b-5p | 54.07 | 96.16 | 96.57 | 94.77 | 106.64 | 75.24 | 69.89 | 69 | 59.97 | 68.58 |
| hsa-miR-100-5p | 43.26 | 78.68 | 45.2 | 107.4 | 101.56 | 96.15 | 83.87 | 94.88 | 62.11 | 76.81 |
| hsa-miR-552 | 68.49 | 96.16 | 110.95 | 69.5 | 86.33 | 62.7 | 87.36 | 56.07 | 64.25 | 87.79 |
| hsa-miR-520c-3p | 41.46 | 52.45 | 59.59 | 145.31 | 86.33 | 75.24 | 115.32 | 84.1 | 47.12 | 82.3 |
| hsa-miR-218-5p | 28.84 | 69.94 | 30.82 | 120.04 | 137.11 | 50.16 | 111.82 | 77.63 | 68.53 | 93.27 |
| hsa-miR-516b-5p | 46.86 | 113.65 | 43.15 | 94.77 | 81.25 | 66.88 | 122.3 | 71.16 | 68.53 | 79.56 |
| hsa-miR-340-5p | 61.28 | 69.94 | 80.13 | 120.04 | 142.19 | 96.15 | 45.43 | 49.6 | 59.97 | 60.35 |
| hsa-miR-450b-3p | 37.85 | 43.71 | 34.93 | 82.13 | 116.8 | 62.7 | 115.32 | 118.6 | 68.53 | 104.25 |
| hsa-miR-127-3p | 36.05 | 69.94 | 86.3 | 56.86 | 96.49 | 71.06 | 108.33 | 81.94 | 70.67 | 106.99 |
| hsa-miR-1233 | 50.47 | 61.19 | 43.15 | 107.4 | 76.17 | 79.43 | 87.36 | 112.13 | 74.96 | 90.53 |
| hsa-miR-132-3p | 32.44 | 96.16 | 36.98 | 107.4 | 132.03 | 75.24 | 80.37 | 73.32 | 77.1 | 71.33 |
| hsa-miR-510 | 46.86 | 96.16 | 45.2 | 82.13 | 132.03 | 96.15 | 101.34 | 69 | 47.12 | 65.84 |
| hsa-miR-1204 | 43.26 | 78.68 | 53.42 | 101.09 | 116.8 | 71.06 | 101.34 | 62.54 | 79.24 | 74.07 |
| hsa-miR-493-3p | 45.06 | 69.94 | 22.6 | 132.67 | 126.95 | 62.7 | 122.3 | 71.16 | 64.25 | 63.1 |
| hsa-miR-1250 | 36.05 | 43.71 | 43.15 | 113.72 | 71.09 | 83.61 | 153.75 | 64.69 | 77.1 | 93.27 |
| hsa-miR-208b | 34.25 | 78.68 | 47.26 | 113.72 | 172.66 | 79.43 | 76.88 | 58.22 | 55.68 | 63.1 |
| hsa-miR-634 | 55.88 | 96.16 | 63.7 | 113.72 | 76.17 | 91.97 | 69.89 | 73.32 | 42.83 | 96.02 |
| hsa-miR-615-5p | 36.05 | 78.68 | 43.15 | 107.4 | 81.25 | 96.15 | 87.36 | 92.72 | 68.53 | 87.79 |
| hsa-miR-219-5p | 32.44 | 78.68 | 57.53 | 69.5 | 106.64 | 71.06 | 118.81 | 77.63 | 77.1 | 87.79 |
| hsa-miR-219-1-3p | 34.25 | 34.97 | 22.6 | 101.09 | 45.7 | 58.52 | 122.3 | 99.19 | 141.35 | 115.22 |
| hsa-miR-2052 | 25.23 | 69.94 | 39.04 | 75.81 | 142.19 | 54.34 | 97.84 | 105.66 | 68.53 | 96.02 |
| hsa-miR-885-3p | 34.25 | 43.71 | 41.09 | 132.67 | 137.11 | 96.15 | 90.85 | 64.69 | 62.11 | 71.33 |
| hsa-miR-135b-5p | 28.84 | 96.16 | 47.26 | 75.81 | 96.49 | 71.06 | 104.83 | 99.19 | 57.82 | 96.02 |
| hsa-miR-632 | 37.85 | 131.13 | 26.71 | 75.81 | 81.25 | 121.23 | 59.4 | 79.79 | 64.25 | 96.02 |
| hsa-miR-4488 | 18.02 | 69.94 | 43.15 | 101.09 | 111.72 | 96.15 | 115.32 | 77.63 | 68.53 | 71.33 |
| hsa-miR-3614-5p | 63.09 | 61.19 | 55.48 | 94.77 | 121.88 | 96.15 | 55.91 | 73.32 | 68.53 | 82.3 |
| hsa-miR-381 | 32.44 | 104.91 | 51.37 | 138.99 | 101.56 | 96.15 | 62.9 | 62.54 | 66.39 | 54.87 |
| hsa-miR-4531 | 63.09 | 78.68 | 57.53 | 75.81 | 106.64 | 91.97 | 73.38 | 79.79 | 79.24 | 65.84 |
| hsa-miR-216a | 37.85 | 43.71 | 55.48 | 145.31 | 76.17 | 54.34 | 104.83 | 99.19 | 51.4 | 101.5 |
| hsa-miR-1307-3p | 45.06 | 69.94 | 34.93 | 120.04 | 132.03 | 79.43 | 80.37 | 71.16 | 85.67 | 49.38 |
| hsa-miR-502-5p | 30.64 | 104.91 | 32.88 | 107.4 | 86.33 | 62.7 | 115.32 | 90.57 | 42.83 | 93.27 |
| hsa-miR-1911-5p | 55.88 | 78.68 | 65.75 | 101.09 | 96.49 | 117.05 | 59.4 | 45.28 | 66.39 | 79.56 |
| hsa-miR-502-3p | 30.64 | 26.23 | 39.04 | 101.09 | 121.88 | 83.61 | 122.3 | 79.79 | 64.25 | 96.02 |
| hsa-miR-541-3p | 34.25 | 87.42 | 45.2 | 101.09 | 76.17 | 104.51 | 69.89 | 81.94 | 104.94 | 57.61 |
| hsa-miR-586 | 43.26 | 61.19 | 53.42 | 107.4 | 96.49 | 75.24 | 104.83 | 81.94 | 62.11 | 76.81 |
| hsa-miR-518a-3p | 54.07 | 61.19 | 47.26 | 120.04 | 86.33 | 45.98 | 136.28 | 75.47 | 66.39 | 68.58 |
| hsa-miR-1258 | 48.67 | 52.45 | 47.26 | 101.09 | 86.33 | 83.61 | 108.33 | 81.94 | 55.68 | 96.02 |
| hsa-miR-942 | 36.05 | 69.94 | 43.15 | 69.5 | 96.49 | 66.88 | 104.83 | 92.72 | 81.38 | 98.76 |
| hsa-miR-937 | 41.46 | 69.94 | 59.59 | 56.86 | 111.72 | 108.69 | 66.39 | 90.57 | 66.39 | 87.79 |
| hsa-miR-409-3p | 43.26 | 34.97 | 30.82 | 120.04 | 126.95 | 96.15 | 62.9 | 90.57 | 59.97 | 93.27 |
| hsa-miR-548ae | 57.68 | 69.94 | 51.37 | 107.4 | 81.25 | 45.98 | 97.84 | 77.63 | 79.24 | 90.53 |
| hsa-miR-548ak | 16.22 | 34.97 | 49.31 | 113.72 | 116.8 | 75.24 | 104.83 | 94.88 | 64.25 | 87.79 |
| hsa-miR-203 | 36.05 | 78.68 | 30.82 | 82.13 | 60.94 | 108.69 | 108.33 | 64.69 | 68.53 | 117.97 |
| hsa-miR-550a-5p | 36.05 | 61.19 | 26.71 | 151.63 | 101.56 | 71.06 | 59.4 | 92.72 | 68.53 | 87.79 |
| hsa-miR-1256 | 37.85 | 43.71 | 57.53 | 101.09 | 96.49 | 54.34 | 108.33 | 81.94 | 81.38 | 93.27 |
| hsa-miR-548i | 50.47 | 61.19 | 63.7 | 126.36 | 91.41 | 91.97 | 59.4 | 73.32 | 74.96 | 63.1 |
| hsa-miR-3184-5p | 43.26 | 34.97 | 47.26 | 75.81 | 116.8 | 83.61 | 90.85 | 92.72 | 89.95 | 76.81 |
| hsa-miR-583 | 52.27 | 131.13 | 86.3 | 88.45 | 81.25 | 71.06 | 52.42 | 49.6 | 62.11 | 76.81 |
| hsa-miR-744-5p | 19.83 | 61.19 | 34.93 | 132.67 | 111.72 | 108.69 | 66.39 | 60.38 | 83.52 | 71.33 |
| hsa-miR-323a-3p | 25.23 | 52.45 | 43.15 | 69.5 | 20.31 | 50.16 | 143.27 | 127.23 | 115.65 | 101.5 |
| hsa-miR-562 | 59.48 | 113.65 | 47.26 | 138.99 | 60.94 | 58.52 | 73.38 | 79.79 | 55.68 | 60.35 |
| hsa-miR-520f | 34.25 | 52.45 | 65.75 | 82.13 | 116.8 | 112.87 | 73.38 | 69 | 53.54 | 87.79 |
| hsa-miR-331-5p | 43.26 | 69.94 | 49.31 | 63.18 | 147.27 | 112.87 | 69.89 | 53.91 | 64.25 | 74.07 |
| hsa-miR-1205 | 19.83 | 52.45 | 49.31 | 94.77 | 111.72 | 75.24 | 118.81 | 97.04 | 59.97 | 68.58 |
| hsa-miR-506-3p | 34.25 | 69.94 | 51.37 | 138.99 | 66.02 | 79.43 | 73.38 | 51.75 | 94.23 | 87.79 |
| hsa-miR-548a-5p | 88.32 | 61.19 | 100.68 | 75.81 | 55.86 | 66.88 | 59.4 | 97.04 | 62.11 | 79.56 |
| hsa-miR-34a-5p | 43.26 | 43.71 | 41.09 | 63.18 | 101.56 | 41.8 | 111.82 | 92.72 | 100.66 | 106.99 |
| hsa-miR-924 | 21.63 | 96.16 | 53.42 | 69.5 | 106.64 | 75.24 | 94.35 | 60.38 | 70.67 | 98.76 |
| hsa-miR-581 | 37.85 | 43.71 | 43.15 | 82.13 | 66.02 | 54.34 | 129.29 | 116.44 | 74.96 | 98.76 |
| hsa-miR-587 | 39.65 | 96.16 | 92.46 | 69.5 | 60.94 | 58.52 | 94.35 | 81.94 | 62.11 | 90.53 |
| hsa-miR-512-3p | 54.07 | 61.19 | 67.81 | 82.13 | 101.56 | 87.79 | 69.89 | 75.47 | 47.12 | 98.76 |
| hsa-miR-134 | 37.85 | 52.45 | 47.26 | 107.4 | 121.88 | 54.34 | 80.37 | 79.79 | 79.24 | 85.04 |
| hsa-miR-378a-3p+hsa-miR-378i | 36.05 | 69.94 | 24.66 | 120.04 | 101.56 | 75.24 | 101.34 | 97.04 | 55.68 | 63.1 |
| hsa-miR-34c-3p | 39.65 | 61.19 | 30.82 | 75.81 | 116.8 | 71.06 | 101.34 | 112.13 | 55.68 | 79.56 |
| hsa-miR-147b | 46.86 | 43.71 | 26.71 | 88.45 | 91.41 | 79.43 | 90.85 | 79.79 | 83.52 | 112.48 |
| hsa-miR-3180-3p | 52.27 | 113.65 | 43.15 | 101.09 | 137.11 | 91.97 | 59.4 | 47.44 | 55.68 | 41.15 |
| hsa-miR-524-3p | 50.47 | 26.23 | 28.77 | 50.54 | 111.72 | 58.52 | 136.28 | 90.57 | 74.96 | 112.48 |
| hsa-miR-885-5p | 34.25 | 52.45 | 69.86 | 82.13 | 60.94 | 45.98 | 104.83 | 114.29 | 85.67 | 87.79 |
| hsa-miR-182-5p | 28.84 | 69.94 | 39.04 | 82.13 | 91.41 | 62.7 | 115.32 | 79.79 | 85.67 | 82.3 |
| hsa-miR-137 | 55.88 | 131.13 | 69.86 | 101.09 | 106.64 | 83.61 | 38.44 | 71.16 | 32.12 | 46.64 |
| hsa-miR-1305 | 61.28 | 69.94 | 76.02 | 107.4 | 96.49 | 96.15 | 66.39 | 47.44 | 49.26 | 65.84 |
| hsa-miR-514a-3p | 30.64 | 96.16 | 32.88 | 176.9 | 116.8 | 62.7 | 55.91 | 38.81 | 53.54 | 71.33 |
| hsa-miR-142-5p | 25.23 | 69.94 | 34.93 | 132.67 | 96.49 | 41.8 | 87.36 | 92.72 | 55.68 | 98.76 |
| hsa-miR-517c-3p+hsa-miR-519a-3p | 34.25 | 78.68 | 39.04 | 132.67 | 111.72 | 71.06 | 87.36 | 45.28 | 57.82 | 76.81 |
| hsa-miR-3676-3p | 54.07 | 87.42 | 65.75 | 107.4 | 101.56 | 108.69 | 73.38 | 43.13 | 42.83 | 49.38 |
| hsa-miR-429 | 43.26 | 43.71 | 55.48 | 56.86 | 111.72 | 79.43 | 122.3 | 75.47 | 70.67 | 74.07 |
| hsa-miR-15b-5p | 45.06 | 34.97 | 65.75 | 88.45 | 106.64 | 91.97 | 69.89 | 71.16 | 70.67 | 87.79 |
| hsa-miR-718 | 41.46 | 26.23 | 47.26 | 101.09 | 71.09 | 75.24 | 101.34 | 99.19 | 92.09 | 76.81 |
| hsa-miR-557 | 19.83 | 96.16 | 18.49 | 107.4 | 81.25 | 71.06 | 101.34 | 84.1 | 64.25 | 87.79 |
| hsa-miR-376a-3p | 45.06 | 43.71 | 57.53 | 82.13 | 71.09 | 79.43 | 62.9 | 101.35 | 81.38 | 106.99 |
| hsa-miR-185-5p | 61.28 | 34.97 | 92.46 | 44.22 | 60.94 | 45.98 | 73.38 | 116.44 | 102.8 | 98.76 |
| hsa-miR-4431 | 41.46 | 69.94 | 45.2 | 69.5 | 45.7 | 58.52 | 136.28 | 92.72 | 81.38 | 90.53 |
| hsa-miR-34b-3p | 28.84 | 104.91 | 41.09 | 101.09 | 121.88 | 83.61 | 87.36 | 58.22 | 49.26 | 54.87 |
| hsa-miR-1282 | 41.46 | 96.16 | 43.15 | 120.04 | 96.49 | 50.16 | 97.84 | 40.97 | 51.4 | 93.27 |
| hsa-miR-198 | 23.43 | 34.97 | 43.15 | 88.45 | 96.49 | 75.24 | 115.32 | 94.88 | 62.11 | 96.02 |
| hsa-miR-483-3p | 41.46 | 78.68 | 30.82 | 69.5 | 142.19 | 104.51 | 48.92 | 53.91 | 57.82 | 101.5 |
| hsa-miR-323b-3p | 41.46 | 61.19 | 41.09 | 113.72 | 66.02 | 62.7 | 108.33 | 79.79 | 74.96 | 79.56 |
| hsa-miR-32-5p | 36.05 | 43.71 | 59.59 | 107.4 | 111.72 | 79.43 | 122.3 | 53.91 | 51.4 | 63.1 |
| hsa-miR-643 | 48.67 | 52.45 | 43.15 | 82.13 | 101.56 | 66.88 | 97.84 | 51.75 | 89.95 | 93.27 |
| hsa-miR-1262 | 55.88 | 87.42 | 65.75 | 107.4 | 96.49 | 100.33 | 83.87 | 28.03 | 55.68 | 46.64 |
| hsa-miR-670 | 39.65 | 61.19 | 34.93 | 101.09 | 132.03 | 75.24 | 83.87 | 40.97 | 81.38 | 76.81 |
| hsa-miR-431-5p | 48.67 | 52.45 | 36.98 | 94.77 | 96.49 | 66.88 | 90.85 | 77.63 | 96.37 | 65.84 |
| hsa-miR-320c | 23.43 | 43.71 | 36.98 | 69.5 | 66.02 | 41.8 | 118.81 | 122.91 | 100.66 | 101.5 |
| hsa-miR-378c | 61.28 | 52.45 | 86.3 | 56.86 | 106.64 | 75.24 | 80.37 | 62.54 | 57.82 | 85.04 |
| hsa-miR-125a-3p | 54.07 | 69.94 | 67.81 | 69.5 | 60.94 | 79.43 | 83.87 | 90.57 | 59.97 | 87.79 |
| hsa-miR-3141 | 32.44 | 69.94 | 49.31 | 101.09 | 81.25 | 50.16 | 80.37 | 88.41 | 66.39 | 104.25 |
| hsa-miR-1251 | 27.04 | 52.45 | 28.77 | 82.13 | 66.02 | 50.16 | 146.77 | 103.51 | 89.95 | 76.81 |
| hsa-miR-3928 | 45.06 | 69.94 | 53.42 | 94.77 | 91.41 | 58.52 | 104.83 | 58.22 | 53.54 | 93.27 |
| hsa-miR-758 | 39.65 | 69.94 | 57.53 | 113.72 | 91.41 | 75.24 | 80.37 | 69 | 49.26 | 76.81 |
| hsa-miR-548w | 48.67 | 43.71 | 45.2 | 69.5 | 76.17 | 45.98 | 94.35 | 92.72 | 102.8 | 101.5 |
| hsa-miR-2110 | 18.02 | 69.94 | 34.93 | 157.95 | 60.94 | 37.62 | 108.33 | 73.32 | 55.68 | 101.5 |
| hsa-miR-190a | 27.04 | 96.16 | 32.88 | 101.09 | 121.88 | 79.43 | 94.35 | 45.28 | 53.54 | 65.84 |
| hsa-miR-320b | 36.05 | 87.42 | 22.6 | 107.4 | 66.02 | 54.34 | 83.87 | 60.38 | 81.38 | 117.97 |
| hsa-miR-1224-5p | 54.07 | 87.42 | 98.63 | 82.13 | 71.09 | 117.05 | 34.94 | 58.22 | 49.26 | 63.1 |
| hsa-miR-1294 | 30.64 | 96.16 | 39.04 | 132.67 | 116.8 | 66.88 | 55.91 | 47.44 | 53.54 | 76.81 |
| hsa-miR-651 | 34.25 | 43.71 | 26.71 | 63.18 | 86.33 | 62.7 | 139.78 | 75.47 | 98.52 | 85.04 |
| hsa-miR-126-3p | 46.86 | 113.65 | 63.7 | 63.18 | 71.09 | 100.33 | 73.38 | 73.32 | 42.83 | 65.84 |
| hsa-miR-10b-5p | 39.65 | 78.68 | 71.92 | 94.77 | 86.33 | 41.8 | 76.88 | 58.22 | 77.1 | 87.79 |
| hsa-miR-146a-5p | 34.25 | 8.74 | 55.48 | 82.13 | 111.72 | 83.61 | 73.38 | 88.41 | 79.24 | 96.02 |
| hsa-miR-650 | 45.06 | 52.45 | 53.42 | 94.77 | 71.09 | 91.97 | 108.33 | 45.28 | 53.54 | 96.02 |
| hsa-miR-875-3p | 32.44 | 26.23 | 45.2 | 63.18 | 50.78 | 91.97 | 146.77 | 60.38 | 111.37 | 82.3 |
| hsa-miR-1276 | 28.84 | 61.19 | 59.59 | 88.45 | 132.03 | 83.61 | 62.9 | 66.85 | 70.67 | 54.87 |
| hsa-miR-665 | 41.46 | 69.94 | 34.93 | 44.22 | 81.25 | 25.08 | 115.32 | 99.19 | 87.81 | 109.74 |
| hsa-miR-561-3p | 52.27 | 96.16 | 32.88 | 63.18 | 101.56 | 83.61 | 76.88 | 58.22 | 70.67 | 71.33 |
| hsa-miR-548m | 72.1 | 69.94 | 67.81 | 63.18 | 71.09 | 91.97 | 73.38 | 43.13 | 68.53 | 85.04 |
| hsa-miR-628-3p | 43.26 | 43.71 | 20.55 | 107.4 | 66.02 | 91.97 | 87.36 | 75.47 | 68.53 | 101.5 |
| hsa-miR-582-3p | 36.05 | 69.94 | 41.09 | 126.36 | 106.64 | 87.79 | 73.38 | 58.22 | 44.97 | 60.35 |
| hsa-miR-588 | 32.44 | 43.71 | 65.75 | 63.18 | 137.11 | 91.97 | 87.36 | 62.54 | 49.26 | 71.33 |
| hsa-miR-554 | 25.23 | 78.68 | 49.31 | 101.09 | 126.95 | 66.88 | 87.36 | 49.6 | 66.39 | 52.12 |
| hsa-miR-483-5p | 46.86 | 61.19 | 49.31 | 94.77 | 106.64 | 79.43 | 55.91 | 71.16 | 64.25 | 74.07 |
| hsa-miR-1914-5p | 36.05 | 78.68 | 41.09 | 151.63 | 106.64 | 58.52 | 62.9 | 45.28 | 66.39 | 54.87 |
| hsa-miR-1273e | 39.65 | 61.19 | 65.75 | 82.13 | 91.41 | 100.33 | 80.37 | 45.28 | 66.39 | 68.58 |
| hsa-miR-498 | 48.67 | 52.45 | 45.2 | 88.45 | 55.86 | 87.79 | 73.38 | 88.41 | 72.82 | 87.79 |
| hsa-miR-199a-3p+hsa-miR-199b-3p | 32.44 | 26.23 | 32.88 | 94.77 | 81.25 | 87.79 | 101.34 | 84.1 | 85.67 | 74.07 |
| hsa-miR-3187-3p | 28.84 | 52.45 | 45.2 | 88.45 | 66.02 | 58.52 | 87.36 | 81.94 | 79.24 | 112.48 |
| hsa-miR-548h-5p | 46.86 | 34.97 | 55.48 | 75.81 | 126.95 | 83.61 | 83.87 | 66.85 | 59.97 | 65.84 |
| hsa-miR-1297 | 28.84 | 43.71 | 47.26 | 56.86 | 162.5 | 75.24 | 97.84 | 64.69 | 51.4 | 71.33 |
| hsa-miR-589-5p | 36.05 | 61.19 | 45.2 | 126.36 | 106.64 | 66.88 | 73.38 | 53.91 | 49.26 | 79.56 |
| hsa-miR-23b-3p | 48.67 | 52.45 | 36.98 | 113.72 | 116.8 | 83.61 | 76.88 | 47.44 | 55.68 | 65.84 |
| hsa-miR-101-3p | 32.44 | 61.19 | 47.26 | 63.18 | 76.17 | 83.61 | 97.84 | 64.69 | 85.67 | 85.04 |
| hsa-miR-507 | 19.83 | 43.71 | 36.98 | 82.13 | 126.95 | 33.44 | 97.84 | 84.1 | 92.09 | 79.56 |
| hsa-miR-1266 | 30.64 | 43.71 | 53.42 | 101.09 | 96.49 | 75.24 | 97.84 | 60.38 | 57.82 | 79.56 |
| hsa-miR-1321 | 50.47 | 69.94 | 45.2 | 132.67 | 86.33 | 96.15 | 73.38 | 45.28 | 36.41 | 60.35 |
| hsa-miR-600 | 50.47 | 61.19 | 32.88 | 63.18 | 66.02 | 54.34 | 125.8 | 69 | 79.24 | 93.27 |
| hsa-miR-933 | 32.44 | 52.45 | 51.37 | 107.4 | 60.94 | 54.34 | 66.39 | 109.98 | 85.67 | 74.07 |
| hsa-miR-639 | 46.86 | 43.71 | 76.02 | 120.04 | 106.64 | 121.23 | 38.44 | 40.97 | 51.4 | 49.38 |
| hsa-miR-215 | 37.85 | 61.19 | 45.2 | 88.45 | 137.11 | 50.16 | 87.36 | 62.54 | 44.97 | 79.56 |
| hsa-miR-548d-5p | 37.85 | 69.94 | 55.48 | 82.13 | 147.27 | 79.43 | 66.39 | 64.69 | 57.82 | 32.92 |
| hsa-miR-548s | 23.43 | 61.19 | 51.37 | 94.77 | 106.64 | 62.7 | 80.37 | 73.32 | 79.24 | 60.35 |
| hsa-miR-675-5p | 39.65 | 78.68 | 53.42 | 132.67 | 121.88 | 100.33 | 27.96 | 49.6 | 44.97 | 43.89 |
| hsa-miR-4448 | 28.84 | 69.94 | 22.6 | 101.09 | 101.56 | 83.61 | 80.37 | 69 | 64.25 | 71.33 |
| hsa-miR-378f | 50.47 | 52.45 | 55.48 | 126.36 | 111.72 | 100.33 | 52.42 | 60.38 | 38.55 | 43.89 |
| hsa-miR-558 | 30.64 | 78.68 | 30.82 | 94.77 | 76.17 | 87.79 | 87.36 | 51.75 | 77.1 | 76.81 |
| hsa-miR-378b | 36.05 | 69.94 | 51.37 | 69.5 | 106.64 | 33.44 | 111.82 | 75.47 | 68.53 | 68.58 |
| hsa-miR-524-5p | 41.46 | 34.97 | 55.48 | 113.72 | 106.64 | 50.16 | 80.37 | 58.22 | 53.54 | 96.02 |
| hsa-miR-654-5p | 12.62 | 69.94 | 49.31 | 63.18 | 96.49 | 62.7 | 80.37 | 86.26 | 87.81 | 79.56 |
| hsa-miR-30b-5p | 45.06 | 43.71 | 47.26 | 50.54 | 55.86 | 58.52 | 132.79 | 107.82 | 64.25 | 82.3 |
| hsa-miR-338-5p | 32.44 | 78.68 | 28.77 | 113.72 | 91.41 | 75.24 | 97.84 | 69 | 51.4 | 49.38 |
| hsa-miR-545-3p | 21.63 | 69.94 | 34.93 | 101.09 | 101.56 | 75.24 | 87.36 | 47.44 | 68.53 | 79.56 |
| hsa-miR-619 | 25.23 | 131.13 | 67.81 | 82.13 | 116.8 | 58.52 | 24.46 | 56.07 | 47.12 | 76.81 |
| hsa-miR-499b-5p | 43.26 | 34.97 | 41.09 | 120.04 | 91.41 | 91.97 | 69.89 | 47.44 | 81.38 | 63.1 |
| hsa-miR-603 | 45.06 | 78.68 | 73.97 | 94.77 | 81.25 | 75.24 | 73.38 | 45.28 | 64.25 | 52.12 |
| hsa-miR-711 | 34.25 | 43.71 | 30.82 | 132.67 | 106.64 | 66.88 | 69.89 | 49.6 | 64.25 | 85.04 |
| hsa-miR-767-5p | 28.84 | 87.42 | 51.37 | 88.45 | 86.33 | 62.7 | 97.84 | 62.54 | 49.26 | 68.58 |
| hsa-miR-421 | 37.85 | 96.16 | 36.98 | 75.81 | 101.56 | 50.16 | 83.87 | 60.38 | 57.82 | 82.3 |
| hsa-miR-197-3p | 46.86 | 87.42 | 41.09 | 101.09 | 76.17 | 96.15 | 55.91 | 58.22 | 53.54 | 65.84 |
| hsa-miR-1264 | 28.84 | 61.19 | 39.04 | 138.99 | 126.95 | 58.52 | 62.9 | 49.6 | 57.82 | 57.61 |
| hsa-miR-3127-5p | 41.46 | 52.45 | 53.42 | 94.77 | 91.41 | 87.79 | 73.38 | 71.16 | 57.82 | 57.61 |
| hsa-miR-548an | 34.25 | 52.45 | 63.7 | 82.13 | 86.33 | 45.98 | 90.85 | 64.69 | 87.81 | 71.33 |
| hsa-miR-567 | 46.86 | 69.94 | 43.15 | 63.18 | 81.25 | 71.06 | 80.37 | 75.47 | 68.53 | 79.56 |
| hsa-miR-648 | 43.26 | 61.19 | 53.42 | 63.18 | 60.94 | 50.16 | 87.36 | 99.19 | 102.8 | 57.61 |
| hsa-miR-3178 | 27.04 | 78.68 | 39.04 | 69.5 | 76.17 | 87.79 | 94.35 | 79.79 | 49.26 | 74.07 |
| hsa-miR-520a-3p | 55.88 | 61.19 | 73.97 | 82.13 | 76.17 | 79.43 | 59.4 | 60.38 | 59.97 | 65.84 |
| hsa-miR-1972 | 32.44 | 69.94 | 51.37 | 88.45 | 91.41 | 71.06 | 55.91 | 71.16 | 79.24 | 63.1 |
| hsa-miR-640 | 46.86 | 113.65 | 80.13 | 82.13 | 60.94 | 117.05 | 52.42 | 45.28 | 38.55 | 35.66 |
| hsa-miR-559 | 43.26 | 78.68 | 24.66 | 107.4 | 101.56 | 45.98 | 45.43 | 75.47 | 62.11 | 87.79 |
| hsa-miR-20a-5p+hsa-miR-20b-5p | 25.23 | 61.19 | 34.93 | 101.09 | 76.17 | 54.34 | 55.91 | 75.47 | 94.23 | 93.27 |
| hsa-miR-200b-3p | 28.84 | 26.23 | 36.98 | 75.81 | 81.25 | 37.62 | 83.87 | 120.76 | 92.09 | 87.79 |
| hsa-miR-1288 | 32.44 | 34.97 | 43.15 | 69.5 | 121.88 | 50.16 | 97.84 | 84.1 | 62.11 | 74.07 |
| hsa-miR-548l | 43.26 | 61.19 | 47.26 | 126.36 | 86.33 | 83.61 | 48.92 | 62.54 | 57.82 | 52.12 |
| hsa-miR-323a-5p | 30.64 | 87.42 | 30.82 | 88.45 | 132.03 | 71.06 | 52.42 | 71.16 | 44.97 | 57.61 |
| hsa-miR-660-5p | 28.84 | 26.23 | 47.26 | 69.5 | 86.33 | 58.52 | 94.35 | 99.19 | 79.24 | 76.81 |
| hsa-miR-488-3p | 34.25 | 69.94 | 41.09 | 56.86 | 106.64 | 62.7 | 76.88 | 58.22 | 66.39 | 93.27 |
| hsa-miR-1273g-5p | 27.04 | 69.94 | 51.37 | 75.81 | 76.17 | 71.06 | 76.88 | 71.16 | 64.25 | 82.3 |
| hsa-miR-342-5p | 34.25 | 34.97 | 36.98 | 63.18 | 76.17 | 45.98 | 97.84 | 77.63 | 94.23 | 104.25 |
| hsa-miR-331-3p | 39.65 | 26.23 | 49.31 | 94.77 | 106.64 | 54.34 | 73.38 | 53.91 | 74.96 | 90.53 |
| hsa-miR-943 | 23.43 | 26.23 | 36.98 | 94.77 | 55.86 | 45.98 | 132.79 | 56.07 | 89.95 | 101.5 |
| hsa-miR-193a-5p | 39.65 | 69.94 | 39.04 | 94.77 | 101.56 | 79.43 | 62.9 | 60.38 | 38.55 | 76.81 |
| hsa-miR-1291 | 64.89 | 61.19 | 71.92 | 63.18 | 81.25 | 71.06 | 69.89 | 64.69 | 34.27 | 79.56 |
| hsa-miR-548y | 46.86 | 61.19 | 49.31 | 126.36 | 91.41 | 96.15 | 45.43 | 38.81 | 51.4 | 54.87 |
| hsa-miR-487b | 41.46 | 17.48 | 47.26 | 101.09 | 96.49 | 66.88 | 80.37 | 64.69 | 59.97 | 85.04 |
| hsa-miR-455-3p | 23.43 | 34.97 | 41.09 | 18.95 | 40.63 | 58.52 | 125.8 | 92.72 | 109.22 | 115.22 |
| hsa-miR-592 | 46.86 | 43.71 | 82.19 | 63.18 | 71.09 | 79.43 | 87.36 | 71.16 | 49.26 | 65.84 |
| hsa-miR-633 | 16.22 | 52.45 | 43.15 | 94.77 | 71.09 | 83.61 | 97.84 | 77.63 | 68.53 | 52.12 |
| hsa-miR-196a-5p | 21.63 | 61.19 | 45.2 | 44.22 | 76.17 | 37.62 | 108.33 | 86.26 | 66.39 | 109.74 |
| hsa-miR-509-3p | 41.46 | 87.42 | 45.2 | 94.77 | 81.25 | 75.24 | 90.85 | 32.35 | 47.12 | 60.35 |
| hsa-miR-219-2-3p | 37.85 | 26.23 | 39.04 | 113.72 | 71.09 | 58.52 | 90.85 | 58.22 | 83.52 | 76.81 |
| hsa-miR-183-5p | 55.88 | 52.45 | 49.31 | 50.54 | 106.64 | 100.33 | 45.43 | 60.38 | 57.82 | 76.81 |
| hsa-miR-599 | 34.25 | 78.68 | 28.77 | 88.45 | 50.78 | 54.34 | 101.34 | 64.69 | 77.1 | 76.81 |
| hsa-miR-135a-5p | 32.44 | 122.39 | 32.88 | 63.18 | 71.09 | 87.79 | 80.37 | 53.91 | 36.41 | 74.07 |
| hsa-miR-1468 | 27.04 | 34.97 | 59.59 | 56.86 | 91.41 | 66.88 | 90.85 | 60.38 | 77.1 | 87.79 |
| hsa-miR-371b-5p | 45.06 | 61.19 | 39.04 | 82.13 | 91.41 | 41.8 | 55.91 | 75.47 | 92.09 | 68.58 |
| hsa-miR-451a | 39.65 | 78.68 | 41.09 | 94.77 | 81.25 | 58.52 | 94.35 | 49.6 | 32.12 | 82.3 |
| hsa-miR-485-3p | 28.84 | 61.19 | 49.31 | 82.13 | 71.09 | 108.69 | 73.38 | 58.22 | 53.54 | 65.84 |
| hsa-miR-1287 | 30.64 | 52.45 | 34.93 | 69.5 | 86.33 | 62.7 | 83.87 | 62.54 | 83.52 | 85.04 |
| hsa-miR-1181 | 34.25 | 61.19 | 51.37 | 94.77 | 81.25 | 20.9 | 76.88 | 77.63 | 70.67 | 82.3 |
| hsa-miR-224-5p | 28.84 | 43.71 | 71.92 | 63.18 | 81.25 | 58.52 | 83.87 | 64.69 | 72.82 | 82.3 |
| hsa-miR-889 | 32.44 | 69.94 | 47.26 | 88.45 | 116.8 | 45.98 | 62.9 | 66.85 | 59.97 | 60.35 |
| hsa-miR-1265 | 41.46 | 113.65 | 45.2 | 132.67 | 106.64 | 45.98 | 52.42 | 23.72 | 40.69 | 46.64 |
| hsa-miR-372 | 34.25 | 26.23 | 43.15 | 120.04 | 101.56 | 87.79 | 80.37 | 40.97 | 51.4 | 63.1 |
| hsa-miR-543 | 46.86 | 43.71 | 69.86 | 94.77 | 106.64 | 58.52 | 69.89 | 51.75 | 42.83 | 63.1 |
| hsa-miR-145-5p | 28.84 | 52.45 | 36.98 | 56.86 | 106.64 | 79.43 | 76.88 | 69 | 49.26 | 90.53 |
| hsa-miR-514b-3p | 27.04 | 43.71 | 47.26 | 101.09 | 137.11 | 50.16 | 48.92 | 56.07 | 57.82 | 76.81 |
| hsa-miR-624-3p | 21.63 | 43.71 | 45.2 | 101.09 | 66.02 | 62.7 | 80.37 | 66.85 | 51.4 | 106.99 |
| hsa-miR-1179 | 23.43 | 61.19 | 51.37 | 63.18 | 126.95 | 45.98 | 69.89 | 88.41 | 64.25 | 49.38 |
| hsa-miR-548v | 37.85 | 34.97 | 47.26 | 94.77 | 71.09 | 79.43 | 66.39 | 58.22 | 79.24 | 74.07 |
| hsa-miR-664-3p | 32.44 | 43.71 | 30.82 | 120.04 | 86.33 | 71.06 | 45.43 | 77.63 | 64.25 | 71.33 |
| hsa-miR-766-3p | 52.27 | 69.94 | 26.71 | 75.81 | 96.49 | 50.16 | 80.37 | 75.47 | 49.26 | 65.84 |
| hsa-miR-1284 | 39.65 | 34.97 | 51.37 | 88.45 | 55.86 | 71.06 | 80.37 | 73.32 | 64.25 | 82.3 |
| hsa-miR-221-3p | 28.84 | 52.45 | 49.31 | 50.54 | 86.33 | 91.97 | 59.4 | 58.22 | 81.38 | 82.3 |
| hsa-miR-4435 | 34.25 | 43.71 | 39.04 | 101.09 | 81.25 | 50.16 | 87.36 | 53.91 | 62.11 | 87.79 |
| hsa-miR-548ab | 50.47 | 87.42 | 41.09 | 75.81 | 86.33 | 83.61 | 59.4 | 58.22 | 51.4 | 46.64 |
| hsa-miR-491-5p | 30.64 | 78.68 | 30.82 | 82.13 | 106.64 | 37.62 | 83.87 | 38.81 | 79.24 | 71.33 |
| hsa-miR-646 | 48.67 | 78.68 | 41.09 | 151.63 | 71.09 | 45.98 | 45.43 | 32.35 | 47.12 | 76.81 |
| hsa-miR-760 | 27.04 | 61.19 | 26.71 | 88.45 | 91.41 | 50.16 | 73.38 | 66.85 | 62.11 | 90.53 |
| hsa-miR-31-5p | 32.44 | 96.16 | 45.2 | 88.45 | 81.25 | 66.88 | 59.4 | 45.28 | 55.68 | 65.84 |
| hsa-let-7e-5p | 36.05 | 87.42 | 63.7 | 88.45 | 96.49 | 54.34 | 41.93 | 75.47 | 32.12 | 60.35 |
| hsa-miR-520e | 34.25 | 87.42 | 36.98 | 94.77 | 101.56 | 87.79 | 59.4 | 38.81 | 53.54 | 41.15 |
| hsa-miR-657 | 37.85 | 52.45 | 32.88 | 50.54 | 96.49 | 29.26 | 90.85 | 75.47 | 81.38 | 85.04 |
| hsa-miR-1245b-3p | 50.47 | 96.16 | 67.81 | 88.45 | 71.09 | 66.88 | 55.91 | 43.13 | 53.54 | 38.41 |
| hsa-miR-300 | 21.63 | 69.94 | 36.98 | 101.09 | 96.49 | 66.88 | 73.38 | 51.75 | 44.97 | 68.58 |
| hsa-miR-106b-5p | 45.06 | 43.71 | 39.04 | 88.45 | 55.86 | 37.62 | 101.34 | 75.47 | 62.11 | 82.3 |
| hsa-miR-202-3p | 52.27 | 43.71 | 61.64 | 63.18 | 71.09 | 71.06 | 76.88 | 62.54 | 59.97 | 68.58 |
| hsa-miR-564 | 27.04 | 43.71 | 18.49 | 88.45 | 66.02 | 25.08 | 104.83 | 84.1 | 79.24 | 93.27 |
| hsa-miR-3123 | 34.25 | 52.45 | 36.98 | 88.45 | 60.94 | 96.15 | 66.39 | 47.44 | 62.11 | 85.04 |
| hsa-miR-376c | 25.23 | 43.71 | 24.66 | 82.13 | 116.8 | 66.88 | 73.38 | 64.69 | 66.39 | 65.84 |
| hsa-miR-1302 | 32.44 | 52.45 | 61.64 | 69.5 | 66.02 | 50.16 | 87.36 | 66.85 | 68.53 | 74.07 |
| hsa-miR-4792 | 28.84 | 34.97 | 36.98 | 101.09 | 121.88 | 75.24 | 48.92 | 47.44 | 62.11 | 71.33 |
| hsa-miR-1237 | 34.25 | 43.71 | 39.04 | 63.18 | 71.09 | 58.52 | 73.38 | 69 | 94.23 | 82.3 |
| hsa-miR-194-5p | 16.22 | 61.19 | 39.04 | 44.22 | 101.56 | 66.88 | 83.87 | 60.38 | 64.25 | 90.53 |
| hsa-miR-522-3p | 43.26 | 43.71 | 59.59 | 31.59 | 116.8 | 58.52 | 87.36 | 58.22 | 64.25 | 63.1 |
| hsa-miR-618 | 36.05 | 61.19 | 41.09 | 75.81 | 91.41 | 62.7 | 97.84 | 47.44 | 40.69 | 71.33 |
| hsa-miR-548ag | 25.23 | 43.71 | 39.04 | 120.04 | 76.17 | 66.88 | 87.36 | 58.22 | 53.54 | 54.87 |
| hsa-miR-302c-3p | 34.25 | 78.68 | 47.26 | 101.09 | 60.94 | 66.88 | 66.39 | 62.54 | 49.26 | 57.61 |
| hsa-miR-1303 | 39.65 | 69.94 | 39.04 | 37.91 | 45.7 | 45.98 | 101.34 | 81.94 | 77.1 | 85.04 |
| hsa-miR-3185 | 30.64 | 61.19 | 22.6 | 120.04 | 101.56 | 45.98 | 59.4 | 66.85 | 59.97 | 54.87 |
| hsa-miR-936 | 32.44 | 43.71 | 41.09 | 50.54 | 96.49 | 79.43 | 94.35 | 53.91 | 42.83 | 87.79 |
| hsa-miR-128 | 34.25 | 87.42 | 20.55 | 69.5 | 106.64 | 54.34 | 90.85 | 53.91 | 38.55 | 65.84 |
| hsa-miR-1255b-5p | 30.64 | 8.74 | 53.42 | 50.54 | 55.86 | 54.34 | 101.34 | 114.29 | 74.96 | 76.81 |
| hsa-miR-610 | 16.22 | 43.71 | 32.88 | 63.18 | 71.09 | 50.16 | 122.3 | 71.16 | 79.24 | 68.58 |
| hsa-miR-661 | 48.67 | 26.23 | 57.53 | 56.86 | 71.09 | 37.62 | 97.84 | 71.16 | 57.82 | 93.27 |
| hsa-miR-499b-3p | 27.04 | 34.97 | 32.88 | 94.77 | 91.41 | 108.69 | 59.4 | 58.22 | 36.41 | 74.07 |
| hsa-miR-500a-5p+hsa-miR-501-5p | 46.86 | 34.97 | 65.75 | 37.91 | 76.17 | 50.16 | 87.36 | 62.54 | 79.24 | 76.81 |
| hsa-miR-27b-3p | 34.25 | 113.65 | 34.93 | 44.22 | 81.25 | 66.88 | 62.9 | 69 | 49.26 | 60.35 |
| hsa-miR-328 | 37.85 | 52.45 | 57.53 | 88.45 | 96.49 | 66.88 | 45.43 | 56.07 | 53.54 | 60.35 |
| hsa-miR-4286 | 50.47 | 26.23 | 67.81 | 44.22 | 55.86 | 79.43 | 62.9 | 71.16 | 62.11 | 93.27 |
| hsa-miR-509-3-5p | 46.86 | 43.71 | 53.42 | 63.18 | 86.33 | 66.88 | 62.9 | 69 | 57.82 | 63.1 |
| hsa-miR-663b | 27.04 | 43.71 | 55.48 | 82.13 | 76.17 | 83.61 | 66.39 | 64.69 | 53.54 | 60.35 |
| hsa-miR-593-3p | 27.04 | 61.19 | 32.88 | 82.13 | 81.25 | 58.52 | 59.4 | 75.47 | 70.67 | 63.1 |
| hsa-miR-518e-3p | 19.83 | 52.45 | 24.66 | 50.54 | 81.25 | 71.06 | 97.84 | 56.07 | 94.23 | 63.1 |
| hsa-miR-486-3p | 28.84 | 43.71 | 28.77 | 82.13 | 81.25 | 50.16 | 73.38 | 66.85 | 72.82 | 82.3 |
| hsa-miR-19b-3p | 45.06 | 61.19 | 51.37 | 94.77 | 71.09 | 62.7 | 66.39 | 58.22 | 49.26 | 49.38 |
| hsa-miR-496 | 52.27 | 26.23 | 43.15 | 56.86 | 60.94 | 75.24 | 76.88 | 71.16 | 96.37 | 49.38 |
| hsa-miR-1180 | 37.85 | 52.45 | 28.77 | 63.18 | 91.41 | 83.61 | 62.9 | 90.57 | 42.83 | 54.87 |
| hsa-miR-892a | 27.04 | 61.19 | 20.55 | 69.5 | 71.09 | 75.24 | 73.38 | 56.07 | 68.53 | 85.04 |
| hsa-miR-140-3p | 23.43 | 43.71 | 32.88 | 37.91 | 76.17 | 75.24 | 94.35 | 60.38 | 72.82 | 90.53 |
| hsa-miR-4451 | 36.05 | 78.68 | 69.86 | 56.86 | 66.02 | 62.7 | 55.91 | 56.07 | 53.54 | 71.33 |
| hsa-miR-1206 | 57.68 | 78.68 | 61.64 | 75.81 | 71.09 | 37.62 | 76.88 | 36.66 | 55.68 | 54.87 |
| hsa-miR-21-5p | 72.1 | 8.74 | 2.05 | 50.54 | 5.08 | 4.18 | 90.85 | 232.89 | 137.06 | 2.74 |
| hsa-miR-1290 | 39.65 | 43.71 | 53.42 | 101.09 | 71.09 | 71.06 | 83.87 | 38.81 | 55.68 | 43.89 |
| hsa-miR-19a-3p | 18.02 | 87.42 | 57.53 | 82.13 | 111.72 | 54.34 | 48.92 | 66.85 | 25.7 | 49.38 |
| hsa-miR-625-5p | 34.25 | 34.97 | 57.53 | 75.81 | 81.25 | 33.44 | 83.87 | 71.16 | 62.11 | 65.84 |
| hsa-miR-2054 | 30.64 | 52.45 | 30.82 | 120.04 | 86.33 | 71.06 | 27.96 | 75.47 | 47.12 | 57.61 |
| hsa-miR-217 | 21.63 | 52.45 | 32.88 | 63.18 | 116.8 | 75.24 | 66.39 | 58.22 | 40.69 | 71.33 |
| hsa-miR-374a-5p | 48.67 | 34.97 | 26.71 | 75.81 | 96.49 | 66.88 | 87.36 | 49.6 | 59.97 | 52.12 |
| hsa-miR-616-3p | 18.02 | 87.42 | 32.88 | 107.4 | 86.33 | 75.24 | 52.42 | 47.44 | 38.55 | 52.12 |
| hsa-miR-551a | 16.22 | 61.19 | 20.55 | 113.72 | 71.09 | 41.8 | 66.39 | 79.79 | 53.54 | 71.33 |
| hsa-miR-369-3p | 30.64 | 52.45 | 47.26 | 82.13 | 71.09 | 75.24 | 59.4 | 51.75 | 53.54 | 71.33 |
| hsa-miR-525-3p | 18.02 | 43.71 | 16.44 | 63.18 | 101.56 | 54.34 | 66.39 | 51.75 | 87.81 | 90.53 |
| hsa-miR-1271-5p | 37.85 | 34.97 | 45.2 | 56.86 | 66.02 | 41.8 | 129.29 | 62.54 | 47.12 | 71.33 |
| hsa-miR-299-3p | 45.06 | 34.97 | 45.2 | 94.77 | 81.25 | 62.7 | 52.42 | 47.44 | 57.82 | 71.33 |
| hsa-miR-653 | 27.04 | 43.71 | 24.66 | 82.13 | 96.49 | 79.43 | 80.37 | 51.75 | 49.26 | 57.61 |
| hsa-miR-369-5p | 14.42 | 52.45 | 30.82 | 94.77 | 66.02 | 45.98 | 94.35 | 64.69 | 51.4 | 76.81 |
| hsa-miR-572 | 16.22 | 52.45 | 43.15 | 101.09 | 96.49 | 71.06 | 59.4 | 32.35 | 49.26 | 68.58 |
| hsa-miR-490-5p | 37.85 | 26.23 | 20.55 | 75.81 | 137.11 | 54.34 | 87.36 | 25.88 | 55.68 | 68.58 |
| hsa-miR-187-3p | 27.04 | 52.45 | 28.77 | 88.45 | 71.09 | 58.52 | 76.88 | 66.85 | 59.97 | 57.61 |
| hsa-miR-875-5p | 30.64 | 52.45 | 45.2 | 94.77 | 76.17 | 66.88 | 55.91 | 53.91 | 53.54 | 57.61 |
| hsa-miR-199a-5p | 48.67 | 17.48 | 65.75 | 75.81 | 71.09 | 58.52 | 41.93 | 66.85 | 74.96 | 65.84 |
| hsa-miR-642a-5p | 28.84 | 87.42 | 47.26 | 107.4 | 91.41 | 41.8 | 48.92 | 36.66 | 40.69 | 54.87 |
| hsa-miR-346 | 32.44 | 61.19 | 55.48 | 94.77 | 55.86 | 54.34 | 55.91 | 60.38 | 53.54 | 60.35 |
| hsa-miR-508-5p | 30.64 | 52.45 | 20.55 | 50.54 | 66.02 | 54.34 | 87.36 | 73.32 | 79.24 | 68.58 |
| hsa-miR-204-5p | 30.64 | 69.94 | 43.15 | 75.81 | 101.56 | 58.52 | 76.88 | 38.81 | 47.12 | 38.41 |
| hsa-miR-573 | 28.84 | 69.94 | 41.09 | 126.36 | 96.49 | 50.16 | 27.96 | 45.28 | 47.12 | 46.64 |
| hsa-miR-374b-5p | 28.84 | 52.45 | 26.71 | 56.86 | 101.56 | 66.88 | 48.92 | 69 | 64.25 | 63.1 |
| hsa-miR-154-5p | 19.83 | 61.19 | 18.49 | 82.13 | 45.7 | 58.52 | 83.87 | 66.85 | 62.11 | 79.56 |
| hsa-miR-449b-5p | 27.04 | 52.45 | 39.04 | 145.31 | 86.33 | 33.44 | 48.92 | 45.28 | 53.54 | 46.64 |
| hsa-miR-378g | 41.46 | 52.45 | 39.04 | 113.72 | 91.41 | 66.88 | 27.96 | 43.13 | 49.26 | 52.12 |
| hsa-miR-1289 | 37.85 | 52.45 | 34.93 | 63.18 | 45.7 | 71.06 | 90.85 | 47.44 | 81.38 | 52.12 |
| hsa-miR-1267 | 63.09 | 69.94 | 45.2 | 82.13 | 76.17 | 45.98 | 45.43 | 53.91 | 55.68 | 38.41 |
| hsa-miR-4284 | 30.64 | 34.97 | 49.31 | 50.54 | 55.86 | 45.98 | 80.37 | 58.22 | 79.24 | 90.53 |
| hsa-miR-877-5p | 43.26 | 34.97 | 47.26 | 50.54 | 25.39 | 54.34 | 115.32 | 81.94 | 55.68 | 65.84 |
| hsa-miR-582-5p | 34.25 | 96.16 | 28.77 | 63.18 | 60.94 | 83.61 | 59.4 | 45.28 | 34.27 | 68.58 |
| hsa-miR-3180-5p | 25.23 | 43.71 | 45.2 | 50.54 | 30.47 | 54.34 | 69.89 | 77.63 | 81.38 | 96.02 |
| hsa-miR-1254 | 34.25 | 26.23 | 41.09 | 44.22 | 81.25 | 62.7 | 83.87 | 56.07 | 62.11 | 82.3 |
| hsa-miR-1292 | 34.25 | 69.94 | 34.93 | 50.54 | 71.09 | 62.7 | 59.4 | 43.13 | 62.11 | 85.04 |
| hsa-miR-571 | 37.85 | 61.19 | 43.15 | 56.86 | 55.86 | 45.98 | 62.9 | 53.91 | 66.39 | 87.79 |
| hsa-miR-1268b | 27.04 | 87.42 | 28.77 | 113.72 | 111.72 | 54.34 | 45.43 | 32.35 | 32.12 | 38.41 |
| hsa-miR-2113 | 37.85 | 34.97 | 82.19 | 56.86 | 60.94 | 45.98 | 76.88 | 73.32 | 55.68 | 46.64 |
| hsa-miR-548o-3p | 28.84 | 17.48 | 36.98 | 69.5 | 86.33 | 41.8 | 101.34 | 69 | 68.53 | 49.38 |
| hsa-miR-608 | 36.05 | 52.45 | 61.64 | 75.81 | 71.09 | 33.44 | 62.9 | 53.91 | 49.26 | 71.33 |
| hsa-miR-765 | 39.65 | 43.71 | 59.59 | 94.77 | 50.78 | 58.52 | 55.91 | 53.91 | 49.26 | 60.35 |
| hsa-miR-1248 | 30.64 | 43.71 | 28.77 | 63.18 | 81.25 | 50.16 | 52.42 | 77.63 | 64.25 | 74.07 |
| hsa-miR-452-5p | 46.86 | 17.48 | 30.82 | 63.18 | 71.09 | 54.34 | 76.88 | 56.07 | 74.96 | 74.07 |
| hsa-miR-1280 | 32.44 | 61.19 | 51.37 | 50.54 | 86.33 | 45.98 | 62.9 | 53.91 | 59.97 | 60.35 |
| hsa-miR-491-3p | 28.84 | 26.23 | 43.15 | 56.86 | 71.09 | 71.06 | 87.36 | 56.07 | 66.39 | 57.61 |
| hsa-miR-188-3p | 34.25 | 52.45 | 45.2 | 82.13 | 76.17 | 50.16 | 59.4 | 49.6 | 49.26 | 65.84 |
| hsa-miR-107 | 36.05 | 17.48 | 34.93 | 75.81 | 71.09 | 33.44 | 108.33 | 51.75 | 66.39 | 68.58 |
| hsa-miR-92b-3p | 34.25 | 61.19 | 36.98 | 63.18 | 111.72 | 45.98 | 62.9 | 43.13 | 49.26 | 54.87 |
| hsa-miR-1269b | 52.27 | 52.45 | 55.48 | 126.36 | 60.94 | 62.7 | 41.93 | 38.81 | 44.97 | 27.43 |
| hsa-miR-1249 | 23.43 | 52.45 | 26.71 | 75.81 | 106.64 | 66.88 | 55.91 | 66.85 | 47.12 | 41.15 |
| hsa-miR-934 | 28.84 | 78.68 | 28.77 | 56.86 | 71.09 | 45.98 | 73.38 | 64.69 | 42.83 | 71.33 |
| hsa-miR-1304-5p | 34.25 | 78.68 | 43.15 | 63.18 | 60.94 | 33.44 | 90.85 | 36.66 | 57.82 | 63.1 |
| hsa-miR-130b-3p | 18.02 | 52.45 | 30.82 | 56.86 | 81.25 | 58.52 | 45.43 | 90.57 | 59.97 | 65.84 |
| hsa-miR-33b-5p | 27.04 | 61.19 | 36.98 | 75.81 | 66.02 | 50.16 | 80.37 | 45.28 | 53.54 | 63.1 |
| hsa-miR-508-3p | 27.04 | 61.19 | 24.66 | 50.54 | 81.25 | 71.06 | 76.88 | 38.81 | 55.68 | 71.33 |
| hsa-miR-576-3p | 28.84 | 26.23 | 28.77 | 101.09 | 66.02 | 62.7 | 73.38 | 77.63 | 40.69 | 52.12 |
| hsa-miR-1537 | 19.83 | 78.68 | 45.2 | 63.18 | 71.09 | 37.62 | 62.9 | 47.44 | 59.97 | 71.33 |
| hsa-miR-505-3p | 39.65 | 34.97 | 47.26 | 75.81 | 81.25 | 71.06 | 48.92 | 51.75 | 47.12 | 57.61 |
| hsa-miR-324-5p | 36.05 | 8.74 | 49.31 | 56.86 | 106.64 | 87.79 | 48.92 | 62.54 | 42.83 | 54.87 |
| hsa-miR-122-5p | 41.46 | 43.71 | 47.26 | 82.13 | 96.49 | 87.79 | 38.44 | 43.13 | 40.69 | 32.92 |
| hsa-miR-662 | 28.84 | 69.94 | 43.15 | 50.54 | 121.88 | 62.7 | 38.44 | 49.6 | 38.55 | 49.38 |
| hsa-miR-1236 | 18.02 | 43.71 | 24.66 | 25.27 | 40.63 | 50.16 | 111.82 | 71.16 | 79.24 | 87.79 |
| hsa-miR-378h | 34.25 | 69.94 | 34.93 | 63.18 | 45.7 | 87.79 | 48.92 | 58.22 | 51.4 | 57.61 |
| hsa-miR-520d-3p | 27.04 | 43.71 | 43.15 | 44.22 | 60.94 | 66.88 | 87.36 | 45.28 | 55.68 | 76.81 |
| hsa-miR-876-5p | 39.65 | 17.48 | 63.7 | 31.59 | 60.94 | 50.16 | 66.39 | 66.85 | 57.82 | 96.02 |
| hsa-miR-597 | 39.65 | 43.71 | 39.04 | 88.45 | 76.17 | 71.06 | 38.44 | 56.07 | 49.26 | 46.64 |
| hsa-miR-708-5p | 39.65 | 26.23 | 49.31 | 56.86 | 86.33 | 58.52 | 59.4 | 47.44 | 64.25 | 60.35 |
| hsa-miR-501-3p | 41.46 | 52.45 | 30.82 | 56.86 | 86.33 | 62.7 | 66.39 | 40.97 | 62.11 | 46.64 |
| hsa-miR-146b-3p | 30.64 | 34.97 | 55.48 | 31.59 | 50.78 | 37.62 | 80.37 | 64.69 | 79.24 | 79.56 |
| hsa-miR-139-5p | 23.43 | 43.71 | 45.2 | 82.13 | 60.94 | 41.8 | 66.39 | 51.75 | 59.97 | 68.58 |
| hsa-miR-542-5p | 39.65 | 69.94 | 36.98 | 82.13 | 71.09 | 45.98 | 66.39 | 47.44 | 34.27 | 49.38 |
| hsa-miR-577 | 23.43 | 43.71 | 24.66 | 82.13 | 91.41 | 50.16 | 55.91 | 58.22 | 49.26 | 60.35 |
| hsa-miR-125a-5p | 30.64 | 52.45 | 32.88 | 101.09 | 40.63 | 66.88 | 45.43 | 77.63 | 47.12 | 43.89 |
| hsa-miR-944 | 54.07 | 78.68 | 36.98 | 82.13 | 50.78 | 33.44 | 38.44 | 62.54 | 53.54 | 46.64 |
| hsa-miR-345-5p | 32.44 | 43.71 | 39.04 | 63.18 | 40.63 | 41.8 | 87.36 | 66.85 | 64.25 | 57.61 |
| hsa-miR-556-3p | 27.04 | 34.97 | 41.09 | 69.5 | 60.94 | 62.7 | 94.35 | 62.54 | 53.54 | 30.18 |
| hsa-miR-1263 | 34.25 | 52.45 | 20.55 | 31.59 | 25.39 | 8.36 | 87.36 | 64.69 | 124.22 | 87.79 |
| hsa-miR-1285-3p | 30.64 | 61.19 | 65.75 | 37.91 | 60.94 | 71.06 | 41.93 | 47.44 | 53.54 | 65.84 |
| hsa-miR-18b-5p | 27.04 | 43.71 | 34.93 | 101.09 | 106.64 | 50.16 | 38.44 | 38.81 | 44.97 | 49.38 |
| hsa-miR-30a-5p | 39.65 | 26.23 | 36.98 | 88.45 | 86.33 | 62.7 | 24.46 | 58.22 | 55.68 | 54.87 |
| hsa-miR-3161 | 23.43 | 69.94 | 59.59 | 50.54 | 96.49 | 25.08 | 62.9 | 45.28 | 40.69 | 57.61 |
| hsa-miR-584-5p | 27.04 | 26.23 | 39.04 | 69.5 | 76.17 | 45.98 | 76.88 | 56.07 | 51.4 | 63.1 |
| hsa-miR-433 | 28.84 | 96.16 | 30.82 | 69.5 | 101.56 | 37.62 | 34.94 | 28.03 | 40.69 | 63.1 |
| hsa-miR-1295a | 28.84 | 69.94 | 61.64 | 88.45 | 60.94 | 45.98 | 41.93 | 49.6 | 38.55 | 43.89 |
| hsa-miR-637 | 16.22 | 34.97 | 24.66 | 107.4 | 66.02 | 41.8 | 59.4 | 56.07 | 64.25 | 57.61 |
| hsa-miR-607 | 37.85 | 26.23 | 45.2 | 56.86 | 71.09 | 37.62 | 66.39 | 51.75 | 55.68 | 79.56 |
| hsa-miR-500b | 30.64 | 34.97 | 39.04 | 56.86 | 71.09 | 79.43 | 52.42 | 56.07 | 44.97 | 60.35 |
| hsa-miR-520g | 30.64 | 87.42 | 32.88 | 82.13 | 60.94 | 79.43 | 41.93 | 34.5 | 36.41 | 38.41 |
| hsa-miR-526b-5p | 32.44 | 43.71 | 45.2 | 88.45 | 55.86 | 58.52 | 52.42 | 47.44 | 53.54 | 46.64 |
| hsa-miR-3154 | 41.46 | 61.19 | 59.59 | 69.5 | 35.55 | 54.34 | 48.92 | 49.6 | 51.4 | 52.12 |
| hsa-miR-29b-3p | 28.84 | 43.71 | 53.42 | 69.5 | 60.94 | 71.06 | 62.9 | 49.6 | 44.97 | 38.41 |
| hsa-miR-523-3p | 32.44 | 61.19 | 59.59 | 44.22 | 81.25 | 71.06 | 41.93 | 36.66 | 44.97 | 49.38 |
| hsa-miR-3195 | 32.44 | 87.42 | 45.2 | 75.81 | 86.33 | 58.52 | 24.46 | 45.28 | 34.27 | 32.92 |
| hsa-miR-92a-3p | 45.06 | 61.19 | 82.19 | 56.86 | 66.02 | 58.52 | 45.43 | 34.5 | 36.41 | 35.66 |
| hsa-miR-497-5p | 28.84 | 78.68 | 41.09 | 69.5 | 55.86 | 66.88 | 83.87 | 32.35 | 36.41 | 27.43 |
| hsa-miR-148a-3p | 25.23 | 26.23 | 162.32 | 50.54 | 50.78 | 41.8 | 48.92 | 49.6 | 32.12 | 32.92 |
| hsa-miR-611 | 21.63 | 34.97 | 32.88 | 88.45 | 40.63 | 45.98 | 76.88 | 47.44 | 59.97 | 71.33 |
| hsa-miR-492 | 21.63 | 69.94 | 28.77 | 69.5 | 60.94 | 41.8 | 66.39 | 64.69 | 47.12 | 49.38 |
| hsa-miR-490-3p | 30.64 | 61.19 | 34.93 | 75.81 | 71.09 | 66.88 | 52.42 | 38.81 | 49.26 | 38.41 |
| hsa-miR-802 | 30.64 | 34.97 | 32.88 | 82.13 | 81.25 | 37.62 | 69.89 | 53.91 | 29.98 | 65.84 |
| hsa-miR-151b | 30.64 | 17.48 | 45.2 | 113.72 | 40.63 | 58.52 | 59.4 | 49.6 | 40.69 | 63.1 |
| hsa-miR-521 | 19.83 | 43.71 | 32.88 | 56.86 | 60.94 | 25.08 | 104.83 | 53.91 | 72.82 | 46.64 |
| hsa-miR-1260b | 32.44 | 78.68 | 30.82 | 88.45 | 81.25 | 66.88 | 31.45 | 30.19 | 49.26 | 27.43 |
| hsa-miR-127-5p | 36.05 | 34.97 | 36.98 | 56.86 | 50.78 | 33.44 | 69.89 | 73.32 | 44.97 | 79.56 |
| hsa-miR-548b-3p | 19.83 | 52.45 | 28.77 | 56.86 | 40.63 | 50.16 | 87.36 | 81.94 | 59.97 | 38.41 |
| hsa-miR-1278 | 37.85 | 34.97 | 30.82 | 44.22 | 106.64 | 54.34 | 38.44 | 69 | 42.83 | 54.87 |
| hsa-miR-378d | 25.23 | 61.19 | 41.09 | 37.91 | 60.94 | 71.06 | 73.38 | 53.91 | 44.97 | 43.89 |
| hsa-miR-3164 | 16.22 | 52.45 | 28.77 | 75.81 | 71.09 | 54.34 | 34.94 | 53.91 | 57.82 | 65.84 |
| hsa-miR-628-5p | 25.23 | 61.19 | 36.98 | 44.22 | 106.64 | 87.79 | 38.44 | 38.81 | 29.98 | 38.41 |
| hsa-miR-299-5p | 45.06 | 61.19 | 41.09 | 50.54 | 45.7 | 50.16 | 66.39 | 49.6 | 44.97 | 52.12 |
| hsa-miR-129-5p | 18.02 | 61.19 | 39.04 | 56.86 | 81.25 | 66.88 | 48.92 | 49.6 | 29.98 | 54.87 |
| hsa-miR-1301 | 36.05 | 61.19 | 57.53 | 63.18 | 101.56 | 33.44 | 27.96 | 28.03 | 47.12 | 49.38 |
| hsa-miR-532-3p | 36.05 | 34.97 | 49.31 | 56.86 | 116.8 | 75.24 | 34.94 | 40.97 | 12.85 | 46.64 |
| hsa-miR-361-5p | 25.23 | 26.23 | 20.55 | 82.13 | 40.63 | 79.43 | 52.42 | 66.85 | 44.97 | 65.84 |
| hsa-miR-1245a | 27.04 | 69.94 | 47.26 | 88.45 | 81.25 | 58.52 | 24.46 | 28.03 | 21.42 | 57.61 |
| hsa-miR-668 | 27.04 | 17.48 | 24.66 | 88.45 | 86.33 | 50.16 | 52.42 | 38.81 | 59.97 | 57.61 |
| hsa-miR-548e | 27.04 | 26.23 | 32.88 | 69.5 | 55.86 | 75.24 | 55.91 | 56.07 | 40.69 | 63.1 |
| hsa-miR-645 | 16.22 | 8.74 | 36.98 | 69.5 | 45.7 | 45.98 | 69.89 | 69 | 66.39 | 74.07 |
| hsa-miR-30d-5p | 36.05 | 34.97 | 34.93 | 75.81 | 66.02 | 29.26 | 52.42 | 81.94 | 51.4 | 38.41 |
| hsa-miR-887 | 39.65 | 52.45 | 51.37 | 31.59 | 66.02 | 62.7 | 59.4 | 49.6 | 38.55 | 49.38 |
| hsa-miR-1269a | 30.64 | 61.19 | 30.82 | 94.77 | 55.86 | 54.34 | 34.94 | 38.81 | 36.41 | 60.35 |
| hsa-miR-544b | 21.63 | 26.23 | 36.98 | 50.54 | 66.02 | 37.62 | 69.89 | 51.75 | 57.82 | 79.56 |
| hsa-miR-576-5p | 21.63 | 34.97 | 53.42 | 25.27 | 76.17 | 41.8 | 66.39 | 53.91 | 66.39 | 57.61 |
| hsa-miR-181b-5p+hsa-miR-181d | 34.25 | 43.71 | 41.09 | 69.5 | 76.17 | 33.44 | 38.44 | 38.81 | 53.54 | 68.58 |
| hsa-miR-339-5p | 27.04 | 26.23 | 34.93 | 82.13 | 25.39 | 66.88 | 69.89 | 30.19 | 68.53 | 65.84 |
| hsa-miR-548q | 61.28 | 17.48 | 47.26 | 12.64 | 66.02 | 37.62 | 62.9 | 45.28 | 70.67 | 74.07 |
| hsa-miR-3144-5p | 45.06 | 43.71 | 34.93 | 63.18 | 30.47 | 50.16 | 83.87 | 32.35 | 64.25 | 46.64 |
| hsa-miR-649 | 34.25 | 43.71 | 53.42 | 63.18 | 66.02 | 87.79 | 34.94 | 30.19 | 36.41 | 43.89 |
| hsa-miR-99a-5p | 30.64 | 43.71 | 39.04 | 37.91 | 45.7 | 50.16 | 59.4 | 47.44 | 70.67 | 68.58 |
| hsa-miR-206 | 27.04 | 52.45 | 47.26 | 69.5 | 45.7 | 79.43 | 73.38 | 32.35 | 29.98 | 32.92 |
| hsa-miR-551b-3p | 23.43 | 17.48 | 39.04 | 75.81 | 66.02 | 41.8 | 83.87 | 36.66 | 44.97 | 60.35 |
| hsa-miR-617 | 34.25 | 87.42 | 36.98 | 44.22 | 76.17 | 54.34 | 34.94 | 36.66 | 34.27 | 49.38 |
| hsa-miR-362-3p | 28.84 | 87.42 | 32.88 | 63.18 | 60.94 | 29.26 | 48.92 | 47.44 | 47.12 | 41.15 |
| hsa-miR-595 | 39.65 | 34.97 | 63.7 | 63.18 | 60.94 | 62.7 | 34.94 | 40.97 | 36.41 | 49.38 |
| hsa-miR-591 | 18.02 | 34.97 | 30.82 | 44.22 | 76.17 | 54.34 | 55.91 | 58.22 | 53.54 | 60.35 |
| hsa-miR-671-3p | 25.23 | 69.94 | 30.82 | 63.18 | 45.7 | 45.98 | 55.91 | 51.75 | 51.4 | 46.64 |
| hsa-miR-98 | 34.25 | 26.23 | 32.88 | 63.18 | 76.17 | 54.34 | 66.39 | 25.88 | 59.97 | 46.64 |
| hsa-miR-1910 | 25.23 | 69.94 | 41.09 | 88.45 | 66.02 | 62.7 | 24.46 | 30.19 | 29.98 | 46.64 |
| hsa-miR-324-3p | 30.64 | 61.19 | 39.04 | 82.13 | 55.86 | 50.16 | 52.42 | 34.5 | 38.55 | 38.41 |
| hsa-miR-199b-5p | 19.83 | 61.19 | 51.37 | 44.22 | 35.55 | 50.16 | 52.42 | 45.28 | 55.68 | 65.84 |
| hsa-miR-30c-5p | 28.84 | 26.23 | 53.42 | 63.18 | 66.02 | 58.52 | 48.92 | 45.28 | 47.12 | 43.89 |
| hsa-miR-181c-5p | 34.25 | 78.68 | 26.71 | 31.59 | 76.17 | 45.98 | 55.91 | 32.35 | 40.69 | 57.61 |
| hsa-miR-455-5p | 23.43 | 43.71 | 28.77 | 101.09 | 40.63 | 58.52 | 45.43 | 34.5 | 51.4 | 52.12 |
| hsa-miR-548j | 36.05 | 43.71 | 26.71 | 31.59 | 60.94 | 45.98 | 66.39 | 43.13 | 53.54 | 68.58 |
| hsa-miR-371b-3p | 36.05 | 34.97 | 41.09 | 63.18 | 45.7 | 45.98 | 66.39 | 56.07 | 38.55 | 46.64 |
| hsa-miR-3151 | 28.84 | 34.97 | 47.26 | 69.5 | 30.47 | 33.44 | 62.9 | 66.85 | 44.97 | 52.12 |
| hsa-miR-1227 | 34.25 | 52.45 | 30.82 | 56.86 | 35.55 | 54.34 | 48.92 | 43.13 | 51.4 | 63.1 |
| hsa-miR-1203 | 10.81 | 26.23 | 47.26 | 44.22 | 40.63 | 33.44 | 73.38 | 64.69 | 64.25 | 65.84 |
| hsa-miR-377-3p | 28.84 | 52.45 | 28.77 | 44.22 | 40.63 | 54.34 | 48.92 | 45.28 | 62.11 | 63.1 |
| hsa-miR-323b-5p | 23.43 | 69.94 | 34.93 | 18.95 | 66.02 | 58.52 | 27.96 | 56.07 | 51.4 | 57.61 |
| hsa-miR-326 | 30.64 | 69.94 | 59.59 | 63.18 | 45.7 | 29.26 | 38.44 | 30.19 | 34.27 | 63.1 |
| hsa-miR-4484 | 21.63 | 52.45 | 26.71 | 18.95 | 91.41 | 50.16 | 48.92 | 53.91 | 44.97 | 54.87 |
| hsa-miR-513b | 30.64 | 52.45 | 47.26 | 37.91 | 81.25 | 66.88 | 45.43 | 32.35 | 36.41 | 32.92 |
| hsa-miR-4521 | 34.25 | 26.23 | 24.66 | 37.91 | 66.02 | 29.26 | 76.88 | 53.91 | 57.82 | 54.87 |
| hsa-miR-379-5p | 34.25 | 52.45 | 45.2 | 31.59 | 55.86 | 54.34 | 45.43 | 38.81 | 44.97 | 57.61 |
| hsa-miR-138-5p | 18.02 | 61.19 | 53.42 | 56.86 | 50.78 | 50.16 | 38.44 | 36.66 | 44.97 | 49.38 |
| hsa-miR-362-5p | 10.81 | 69.94 | 47.26 | 69.5 | 45.7 | 45.98 | 45.43 | 30.19 | 44.97 | 46.64 |
| hsa-miR-3192 | 23.43 | 17.48 | 24.66 | 69.5 | 55.86 | 33.44 | 62.9 | 53.91 | 68.53 | 46.64 |
| hsa-miR-133a | 19.83 | 69.94 | 34.93 | 56.86 | 40.63 | 41.8 | 41.93 | 56.07 | 36.41 | 57.61 |
| hsa-miR-513c-5p | 23.43 | 26.23 | 24.66 | 56.86 | 45.7 | 45.98 | 55.91 | 47.44 | 66.39 | 63.1 |
| hsa-miR-1273c | 50.47 | 34.97 | 49.31 | 101.09 | 45.7 | 62.7 | 27.96 | 25.88 | 23.56 | 32.92 |
| hsa-miR-296-5p | 10.81 | 43.71 | 43.15 | 56.86 | 55.86 | 62.7 | 48.92 | 47.44 | 38.55 | 43.89 |
| hsa-miR-615-3p | 16.22 | 43.71 | 18.49 | 44.22 | 40.63 | 71.06 | 90.85 | 53.91 | 25.7 | 46.64 |
| hsa-miR-186-5p | 30.64 | 52.45 | 30.82 | 18.95 | 50.78 | 45.98 | 55.91 | 45.28 | 53.54 | 63.1 |
| hsa-miR-1243 | 34.25 | 43.71 | 22.6 | 44.22 | 101.56 | 41.8 | 24.46 | 43.13 | 36.41 | 54.87 |
| hsa-miR-720 | 41.46 | 52.45 | 41.09 | 44.22 | 20.31 | 41.8 | 41.93 | 51.75 | 47.12 | 63.1 |
| hsa-miR-609 | 18.02 | 34.97 | 20.55 | 69.5 | 71.09 | 66.88 | 59.4 | 34.5 | 25.7 | 43.89 |
| hsa-miR-921 | 27.04 | 34.97 | 30.82 | 63.18 | 50.78 | 45.98 | 45.43 | 58.22 | 40.69 | 46.64 |
| hsa-miR-184 | 18.02 | 34.97 | 41.09 | 56.86 | 50.78 | 29.26 | 55.91 | 43.13 | 47.12 | 65.84 |
| hsa-miR-2277-5p | 12.62 | 34.97 | 14.38 | 44.22 | 40.63 | 37.62 | 87.36 | 71.16 | 55.68 | 43.89 |
| hsa-miR-509-5p | 32.44 | 8.74 | 51.37 | 50.54 | 60.94 | 54.34 | 59.4 | 40.97 | 29.98 | 49.38 |
| hsa-miR-642b-3p | 21.63 | 52.45 | 36.98 | 56.86 | 96.49 | 37.62 | 34.94 | 19.41 | 42.83 | 38.41 |
| hsa-miR-373-3p | 34.25 | 61.19 | 32.88 | 63.18 | 60.94 | 58.52 | 27.96 | 45.28 | 27.84 | 24.69 |
| hsa-miR-1207-3p | 36.05 | 52.45 | 34.93 | 37.91 | 25.39 | 50.16 | 55.91 | 43.13 | 51.4 | 46.64 |
| hsa-miR-454-3p | 27.04 | 8.74 | 36.98 | 25.27 | 25.39 | 41.8 | 73.38 | 53.91 | 72.82 | 68.58 |
| hsa-miR-146b-5p | 25.23 | 17.48 | 24.66 | 50.54 | 76.17 | 45.98 | 45.43 | 53.91 | 36.41 | 57.61 |
| hsa-miR-517b-3p | 39.65 | 52.45 | 34.93 | 44.22 | 81.25 | 54.34 | 45.43 | 25.88 | 21.42 | 32.92 |
| hsa-miR-181a-5p | 28.84 | 52.45 | 43.15 | 44.22 | 45.7 | 66.88 | 48.92 | 36.66 | 29.98 | 35.66 |
| hsa-miR-1293 | 18.02 | 43.71 | 34.93 | 69.5 | 55.86 | 62.7 | 27.96 | 32.35 | 38.55 | 46.64 |
| hsa-miR-24-3p | 34.25 | 34.97 | 30.82 | 37.91 | 30.47 | 41.8 | 41.93 | 90.57 | 32.12 | 54.87 |
| hsa-miR-1260a | 14.42 | 69.94 | 39.04 | 31.59 | 60.94 | 71.06 | 34.94 | 40.97 | 27.84 | 35.66 |
| hsa-miR-1261 | 41.46 | 26.23 | 45.2 | 69.5 | 50.78 | 37.62 | 48.92 | 34.5 | 34.27 | 35.66 |
| hsa-miR-548f | 32.44 | 34.97 | 30.82 | 50.54 | 66.02 | 54.34 | 45.43 | 25.88 | 34.27 | 49.38 |
| hsa-miR-920 | 18.02 | 17.48 | 32.88 | 18.95 | 91.41 | 66.88 | 55.91 | 43.13 | 36.41 | 41.15 |
| hsa-miR-548ac | 36.05 | 34.97 | 36.98 | 69.5 | 60.94 | 41.8 | 24.46 | 38.81 | 36.41 | 38.41 |
| hsa-miR-423-5p | 25.23 | 34.97 | 14.38 | 50.54 | 50.78 | 20.9 | 55.91 | 73.32 | 42.83 | 49.38 |
| hsa-miR-365a-3p | 23.43 | 61.19 | 20.55 | 37.91 | 60.94 | 25.08 | 48.92 | 32.35 | 47.12 | 60.35 |
| hsa-miR-130a-3p | 28.84 | 52.45 | 24.66 | 31.59 | 30.47 | 37.62 | 48.92 | 51.75 | 36.41 | 74.07 |
| hsa-miR-147a | 27.04 | 43.71 | 28.77 | 31.59 | 50.78 | 41.8 | 31.45 | 56.07 | 38.55 | 65.84 |
| hsa-miR-1913 | 18.02 | 78.68 | 26.71 | 63.18 | 76.17 | 45.98 | 24.46 | 30.19 | 21.42 | 30.18 |
| hsa-miR-374c-5p | 52.27 | 52.45 | 24.66 | 37.91 | 55.86 | 33.44 | 38.44 | 30.19 | 44.97 | 43.89 |
| hsa-miR-1306-3p | 23.43 | 26.23 | 30.82 | 56.86 | 101.56 | 62.7 | 38.44 | 28.03 | 25.7 | 19.2 |
| hsa-miR-1247-5p | 23.43 | 34.97 | 18.49 | 56.86 | 66.02 | 50.16 | 48.92 | 38.81 | 40.69 | 32.92 |
| hsa-miR-298 | 16.22 | 26.23 | 14.38 | 25.27 | 35.55 | 37.62 | 73.38 | 56.07 | 44.97 | 79.56 |
| hsa-miR-1273f | 32.44 | 34.97 | 30.82 | 82.13 | 60.94 | 33.44 | 38.44 | 25.88 | 38.55 | 30.18 |
| hsa-miR-1184 | 43.26 | 8.74 | 49.31 | 44.22 | 45.7 | 37.62 | 52.42 | 28.03 | 53.54 | 41.15 |
| hsa-miR-1229 | 27.04 | 52.45 | 28.77 | 31.59 | 30.47 | 25.08 | 55.91 | 51.75 | 38.55 | 60.35 |
| hsa-miR-940 | 23.43 | 69.94 | 28.77 | 50.54 | 55.86 | 45.98 | 10.48 | 36.66 | 49.26 | 30.18 |
| hsa-miR-103a-3p | 25.23 | 17.48 | 36.98 | 69.5 | 76.17 | 54.34 | 31.45 | 32.35 | 36.41 | 16.46 |
| hsa-miR-652-3p | 19.83 | 43.71 | 22.6 | 56.86 | 71.09 | 58.52 | 38.44 | 23.72 | 29.98 | 30.18 |
| hsa-miR-1976 | 18.02 | 26.23 | 28.77 | 44.22 | 55.86 | 50.16 | 59.4 | 45.28 | 25.7 | 41.15 |
| hsa-miR-371a-5p | 16.22 | 17.48 | 22.6 | 37.91 | 35.55 | 33.44 | 66.39 | 43.13 | 53.54 | 65.84 |
| hsa-miR-2115-5p | 23.43 | 17.48 | 28.77 | 56.86 | 45.7 | 37.62 | 52.42 | 49.6 | 38.55 | 41.15 |
| hsa-miR-555 | 28.84 | 78.68 | 26.71 | 63.18 | 45.7 | 66.88 | 27.96 | 12.94 | 17.13 | 21.95 |
| hsa-miR-874 | 19.83 | 34.97 | 28.77 | 56.86 | 55.86 | 45.98 | 48.92 | 43.13 | 27.84 | 27.43 |
| hsa-miR-1915-3p | 23.43 | 8.74 | 26.71 | 44.22 | 66.02 | 29.26 | 48.92 | 60.38 | 34.27 | 46.64 |
| hsa-miR-150-5p | 45.06 | 43.71 | 22.6 | 44.22 | 30.47 | 33.44 | 34.94 | 56.07 | 29.98 | 46.64 |
| hsa-miR-602 | 23.43 | 61.19 | 30.82 | 50.54 | 55.86 | 45.98 | 34.94 | 25.88 | 36.41 | 21.95 |
| hsa-miR-190b | 21.63 | 43.71 | 34.93 | 37.91 | 55.86 | 50.16 | 34.94 | 36.66 | 32.12 | 38.41 |
| hsa-miR-623 | 21.63 | 17.48 | 36.98 | 44.22 | 35.55 | 58.52 | 27.96 | 40.97 | 47.12 | 54.87 |
| hsa-miR-136-5p | 10.81 | 34.97 | 32.88 | 44.22 | 30.47 | 45.98 | 55.91 | 49.6 | 47.12 | 32.92 |
| hsa-miR-425-5p | 23.43 | 17.48 | 36.98 | 44.22 | 30.47 | 20.9 | 45.43 | 51.75 | 55.68 | 49.38 |
| hsa-miR-4443 | 1.8 | 8.74 | 2.05 | 107.4 | 101.56 | 4.18 | 136.28 | 2.16 | 2.14 | 2.74 |
| hsa-miR-620 | 32.44 | 17.48 | 22.6 | 37.91 | 55.86 | 50.16 | 27.96 | 38.81 | 34.27 | 49.38 |
| hsa-miR-1224-3p | 19.83 | 61.19 | 22.6 | 31.59 | 55.86 | 45.98 | 27.96 | 36.66 | 23.56 | 41.15 |
| hsa-miR-1182 | 27.04 | 26.23 | 41.09 | 44.22 | 40.63 | 66.88 | 31.45 | 25.88 | 21.42 | 41.15 |
| hsa-miR-518d-3p | 36.05 | 17.48 | 26.71 | 82.13 | 30.47 | 25.08 | 24.46 | 45.28 | 27.84 | 49.38 |
| hsa-miR-548u | 19.83 | 52.45 | 22.6 | 37.91 | 50.78 | 37.62 | 31.45 | 53.91 | 12.85 | 35.66 |
| hsa-miR-636 | 37.85 | 43.71 | 34.93 | 50.54 | 20.31 | 16.72 | 55.91 | 10.78 | 40.69 | 41.15 |
| hsa-miR-1469 | 27.04 | 43.71 | 18.49 | 25.27 | 25.39 | 37.62 | 52.42 | 45.28 | 36.41 | 35.66 |
| hsa-miR-296-3p | 28.84 | 34.97 | 24.66 | 37.91 | 60.94 | 33.44 | 34.94 | 19.41 | 32.12 | 35.66 |
| hsa-miR-539-5p | 30.64 | 34.97 | 39.04 | 44.22 | 35.55 | 37.62 | 34.94 | 34.5 | 25.7 | 24.69 |
| hsa-miR-548b-5p | 16.22 | 43.71 | 34.93 | 25.27 | 40.63 | 29.26 | 45.43 | 34.5 | 29.98 | 30.18 |
| hsa-miR-518f-3p | 25.23 | 26.23 | 30.82 | 56.86 | 45.7 | 41.8 | 27.96 | 32.35 | 12.85 | 19.2 |
| hsa-miR-873-5p | 10.81 | 17.48 | 24.66 | 50.54 | 30.47 | 25.08 | 45.43 | 21.56 | 42.83 | 46.64 |
| hsa-miR-1296 | 14.42 | 8.74 | 18.49 | 50.54 | 55.86 | 50.16 | 20.97 | 45.28 | 23.56 | 24.69 |
| hsa-miR-1253 | 1.8 | 8.74 | 2.05 | 94.77 | 116.8 | 4.18 | 48.92 | 2.16 | 2.14 | 2.74 |
| hsa-miR-1246 | 1.8 | 34.97 | 2.05 | 88.45 | 10.16 | 12.54 | 90.85 | 2.16 | 2.14 | 2.74 |
| hsa-miR-143-3p | 1.8 | 8.74 | 2.05 | 88.45 | 111.72 | 4.18 | 3.49 | 2.16 | 2.14 | 2.74 |
| hsa-miR-4485 | 19.83 | 8.74 | 30.82 | 12.64 | 5.08 | 16.72 | 10.48 | 19.41 | 21.42 | 13.72 |
| hsa-miR-155-5p | 1.8 | 8.74 | 36.98 | 31.59 | 15.23 | 4.18 | 52.42 | 2.16 | 2.14 | 2.74 |
| hsa-miR-363-3p | 1.8 | 8.74 | 2.05 | 44.22 | 10.16 | 58.52 | 13.98 | 2.16 | 2.14 | 2.74 |
| hsa-miR-1283 | 1.8 | 8.74 | 2.05 | 6.32 | 5.08 | 4.18 | 3.49 | 2.16 | 2.14 | 2.74 |
| hsa-miR-549 | 1.8 | 8.74 | 2.05 | 6.32 | 5.08 | 4.18 | 3.49 | 2.16 | 2.14 | 2.74 |
