# Supplementary material for: Airway Secretory microRNAome Changes during Rhinovirus Infection in Early Childhood
Source: PLoS One. 2016 Sep 19;11(9):e0162244. doi: 10.1371/journal.pone.0162244 (PMC5028059; doi:10.1371/journal.pone.0162244)
Supplement: S3 Table — (DOCX) [file pone.0162244.s003.docx]

**Table 3 S Nasal airway extracellular miRs in RV infected subjects (n=10 children)**

|  | **RV1** | **RV2** | **RV3** | **RV4** | **RV5** | **RV6** | **RV7** | **RV8** | **RV9** | **RV10** |
| --- | --- | --- | --- | --- | --- | --- | --- | --- | --- | --- |
| hsa-miR-630 | 804.82 | 5039.21 | 124.52 | 283.93 | 1088.28 | 395.8 | 156.69 | 577.63 | 755.46 | 144.98 |
| hsa-miR-320e | 539.82 | 1611.63 | 134.73 | 204.49 | 3487.98 | 1696.3 | 146.77 | 567.1 | 228.4 | 178.75 |
| hsa-miR-155-5p | 112.87 | 28.42 | 108.19 | 4912.11 | 58.59 | 80.78 | 224.13 | 258.73 | 1367.86 | 67.53 |
| hsa-miR-378e | 225.74 | 995.93 | 640.98 | 478.12 | 479.94 | 268.58 | 696.19 | 713.01 | 85.33 | 657.39 |
| hsa-miR-21-5p | 107.96 | 477.67 | 620.56 | 476.65 | 716.79 | 173.67 | 811.23 | 720.53 | 148.08 | 631.57 |
| hsa-miR-302d-3p | 554.54 | 165.09 | 412.35 | 335.42 | 311.65 | 373.59 | 450.24 | 449.77 | 557.18 | 361.46 |
| hsa-miR-144-3p | 299.35 | 227.33 | 265.37 | 263.33 | 193.22 | 100.97 | 374.87 | 300.85 | 133.02 | 482.61 |
| hsa-miR-25-3p | 206.11 | 186.74 | 289.87 | 176.54 | 224.39 | 238.29 | 440.32 | 355 | 115.45 | 256.2 |
| hsa-miR-188-5p | 171.76 | 136.67 | 234.75 | 188.3 | 198.21 | 183.77 | 347.1 | 243.69 | 170.67 | 315.78 |
| hsa-miR-495 | 137.41 | 47.36 | 240.88 | 185.36 | 203.2 | 232.23 | 249.91 | 272.27 | 195.77 | 299.89 |
| hsa-miR-579 | 147.22 | 143.44 | 277.62 | 172.12 | 211.92 | 143.38 | 327.27 | 257.23 | 110.43 | 242.3 |
| hsa-miR-375 | 147.22 | 54.13 | 206.17 | 155.94 | 147.1 | 270.6 | 202.31 | 201.57 | 250.98 | 276.06 |
| hsa-miR-548al | 58.89 | 24.36 | 249.04 | 179.48 | 182 | 228.19 | 238.01 | 249.7 | 228.4 | 264.15 |
| hsa-miR-570-3p | 73.61 | 127.2 | 263.33 | 161.82 | 201.95 | 153.47 | 259.83 | 278.29 | 153.1 | 226.41 |
| hsa-miR-631 | 112.87 | 139.38 | 255.17 | 158.88 | 175.77 | 147.42 | 305.45 | 215.11 | 125.49 | 220.45 |
| hsa-miR-4516 | 157.04 | 170.5 | 187.8 | 86.8 | 246.83 | 228.19 | 89.25 | 126.36 | 358.91 | 77.46 |
| hsa-miR-125b-5p | 260.1 | 196.21 | 230.67 | 139.76 | 93.49 | 199.92 | 144.79 | 165.47 | 100.39 | 190.66 |
| hsa-miR-612 | 348.43 | 104.19 | 130.65 | 132.4 | 68.56 | 183.77 | 150.74 | 145.91 | 225.89 | 144.98 |
| hsa-miR-192-5p | 299.35 | 105.55 | 179.64 | 98.57 | 124.66 | 147.42 | 174.54 | 142.9 | 193.26 | 158.88 |
| hsa-miR-1283 | 14.72 | 836.26 | 14.29 | 1.47 | 1.25 | 652.27 | 1.98 | 49.64 | 2.51 | 43.69 |
| hsa-miR-574-5p | 112.87 | 67.66 | 193.93 | 154.47 | 164.55 | 149.44 | 245.95 | 141.4 | 170.67 | 190.66 |
| hsa-miR-1183 | 206.11 | 86.6 | 193.93 | 113.28 | 172.03 | 173.67 | 97.19 | 171.48 | 125.49 | 216.48 |
| hsa-miR-1827 | 196.3 | 77.13 | 206.17 | 108.86 | 117.18 | 131.26 | 158.68 | 148.92 | 208.32 | 156.9 |
| hsa-miR-499a-3p | 137.41 | 39.24 | 185.76 | 114.75 | 119.67 | 171.65 | 208.26 | 157.95 | 205.81 | 156.9 |
| hsa-miR-598 | 98.15 | 67.66 | 238.84 | 151.53 | 172.03 | 137.32 | 111.07 | 136.89 | 117.96 | 212.51 |
| hsa-miR-601 | 196.3 | 251.69 | 75.53 | 73.56 | 157.07 | 127.22 | 103.14 | 129.37 | 188.24 | 103.28 |
| hsa-miR-450b-5p | 137.41 | 41.95 | 175.55 | 89.74 | 108.45 | 141.36 | 182.48 | 176 | 188.24 | 152.93 |
| hsa-miR-222-3p | 147.22 | 106.9 | 157.18 | 108.86 | 109.7 | 76.74 | 148.76 | 160.95 | 140.55 | 158.88 |
| hsa-miR-516a-5p | 78.52 | 35.18 | 163.31 | 122.1 | 102.22 | 173.67 | 142.81 | 176 | 158.12 | 162.86 |
| hsa-miR-384 | 132.5 | 27.06 | 140.85 | 114.75 | 99.73 | 143.38 | 150.74 | 144.41 | 188.24 | 166.83 |
| hsa-miR-548ad | 152.13 | 48.71 | 146.98 | 107.39 | 109.7 | 123.18 | 178.51 | 120.34 | 160.63 | 160.87 |
| hsa-miR-1277-3p | 107.96 | 27.06 | 171.47 | 104.45 | 91 | 197.9 | 162.64 | 132.37 | 145.57 | 162.86 |
| hsa-miR-149-5p | 166.85 | 120.43 | 122.48 | 107.39 | 94.74 | 113.09 | 146.77 | 147.42 | 102.9 | 172.79 |
| hsa-miR-448 | 161.95 | 39.24 | 151.06 | 98.57 | 102.22 | 149.44 | 152.72 | 102.29 | 165.65 | 168.81 |
| hsa-miR-548d-3p | 83.43 | 44.65 | 198.01 | 100.04 | 119.67 | 127.22 | 160.66 | 165.47 | 140.55 | 146.97 |
| hsa-miR-370 | 117.78 | 32.48 | 171.47 | 104.45 | 115.93 | 139.34 | 138.84 | 135.38 | 150.59 | 172.79 |
| hsa-miR-342-3p | 78.52 | 29.77 | 130.65 | 101.51 | 93.49 | 155.49 | 156.69 | 156.44 | 155.61 | 180.73 |
| hsa-miR-627 | 191.39 | 62.25 | 136.77 | 79.44 | 95.99 | 141.36 | 150.74 | 136.89 | 140.55 | 101.29 |
| hsa-miR-656 | 132.5 | 37.89 | 124.52 | 77.97 | 110.95 | 151.45 | 168.59 | 117.33 | 135.53 | 178.75 |
| hsa-miR-518b | 98.15 | 47.36 | 157.18 | 82.38 | 124.66 | 139.34 | 128.92 | 133.88 | 148.08 | 164.84 |
| hsa-miR-4508 | 58.89 | 27.06 | 151.06 | 89.74 | 119.67 | 131.26 | 150.74 | 151.93 | 160.63 | 182.72 |
| hsa-miR-761 | 157.04 | 50.07 | 173.51 | 86.8 | 104.71 | 113.09 | 99.17 | 139.89 | 143.06 | 146.97 |
| hsa-miR-4454 | 137.41 | 51.42 | 132.69 | 89.74 | 118.43 | 115.11 | 103.14 | 121.84 | 203.3 | 139.02 |
| hsa-miR-4425 | 181.58 | 41.95 | 116.36 | 91.21 | 82.28 | 141.36 | 136.86 | 127.86 | 155.61 | 127.11 |
| hsa-miR-654-3p | 107.96 | 37.89 | 153.1 | 85.33 | 99.73 | 147.42 | 130.91 | 120.34 | 173.18 | 144.98 |
| hsa-miR-515-5p | 137.41 | 39.24 | 151.06 | 75.03 | 87.26 | 159.53 | 158.68 | 117.33 | 122.98 | 139.02 |
| hsa-miR-338-3p | 122.69 | 97.43 | 183.72 | 116.22 | 104.71 | 102.99 | 134.87 | 109.81 | 75.3 | 131.08 |
| hsa-miR-888-5p | 103.06 | 56.83 | 142.89 | 89.74 | 130.89 | 84.81 | 150.74 | 154.94 | 122.98 | 141.01 |
| hsa-miR-548z | 117.78 | 58.19 | 132.69 | 69.14 | 113.44 | 88.85 | 113.06 | 109.81 | 233.42 | 131.08 |
| hsa-miR-548x-3p | 206.11 | 55.48 | 100.03 | 98.57 | 67.32 | 117.13 | 107.11 | 118.84 | 128 | 164.84 |
| hsa-miR-2682-5p | 107.96 | 102.84 | 155.14 | 86.8 | 82.28 | 86.83 | 174.54 | 114.32 | 75.3 | 170.8 |
| hsa-miR-548ah-5p | 142.32 | 67.66 | 93.9 | 89.74 | 94.74 | 109.05 | 124.96 | 127.86 | 125.49 | 172.79 |
| hsa-miR-489 | 117.78 | 116.37 | 138.81 | 98.57 | 119.67 | 78.76 | 156.69 | 129.37 | 62.75 | 129.09 |
| hsa-miR-580 | 73.61 | 21.65 | 185.76 | 58.85 | 103.47 | 113.09 | 140.82 | 120.34 | 185.73 | 143 |
| hsa-miR-302e | 142.32 | 62.25 | 159.22 | 73.56 | 112.19 | 113.09 | 136.86 | 126.36 | 110.43 | 109.23 |
| hsa-miR-548k | 157.04 | 40.6 | 120.44 | 98.57 | 73.55 | 113.09 | 124.96 | 135.38 | 150.59 | 113.21 |
| hsa-miR-1323 | 157.04 | 44.65 | 134.73 | 110.33 | 79.78 | 115.11 | 109.09 | 118.84 | 150.59 | 105.26 |
| hsa-miR-297 | 127.59 | 83.9 | 108.19 | 89.74 | 91 | 139.34 | 158.68 | 121.84 | 75.3 | 129.09 |
| hsa-miR-1185-5p | 73.61 | 18.94 | 169.43 | 86.8 | 100.97 | 125.2 | 134.87 | 108.31 | 138.04 | 158.88 |
| hsa-miR-548p | 112.87 | 44.65 | 120.44 | 95.62 | 93.49 | 105.01 | 115.04 | 133.88 | 117.96 | 168.81 |
| hsa-miR-2116-5p | 112.87 | 69.01 | 149.02 | 98.57 | 87.26 | 109.05 | 136.86 | 153.43 | 70.28 | 117.18 |
| hsa-miR-10a-5p | 132.5 | 69.01 | 140.85 | 88.27 | 102.22 | 90.87 | 150.74 | 105.3 | 67.77 | 141.01 |
| hsa-miR-2053 | 186.48 | 66.31 | 91.86 | 70.61 | 69.81 | 151.45 | 105.12 | 97.78 | 120.47 | 121.15 |
| hsa-miR-4455 | 117.78 | 60.89 | 136.77 | 83.85 | 99.73 | 135.3 | 120.99 | 100.78 | 87.84 | 133.07 |
| hsa-miR-1286 | 176.67 | 62.25 | 122.48 | 70.61 | 73.55 | 107.03 | 122.97 | 97.78 | 110.43 | 127.11 |
| hsa-miR-544a | 157.04 | 54.13 | 136.77 | 91.21 | 94.74 | 115.11 | 101.16 | 108.31 | 115.45 | 91.36 |
| hsa-miR-412 | 220.84 | 46.01 | 126.56 | 61.79 | 83.52 | 133.28 | 107.11 | 105.3 | 95.37 | 81.43 |
| hsa-miR-514b-5p | 83.43 | 56.83 | 130.65 | 107.39 | 86.02 | 131.26 | 122.97 | 139.89 | 70.28 | 127.11 |
| hsa-miR-30e-5p | 176.67 | 58.19 | 106.15 | 70.61 | 93.49 | 90.87 | 158.68 | 129.37 | 77.81 | 91.36 |
| hsa-miR-519b-5p+hsa-miR-519c-5p | 137.41 | 29.77 | 122.48 | 58.85 | 76.04 | 131.26 | 111.07 | 133.88 | 122.98 | 125.12 |
| hsa-miR-568 | 49.07 | 25.71 | 155.14 | 80.91 | 93.49 | 147.42 | 109.09 | 133.88 | 135.53 | 117.18 |
| hsa-miR-1972 | 83.43 | 33.83 | 93.9 | 48.55 | 335.33 | 54.52 | 63.47 | 97.78 | 87.84 | 141.01 |
| hsa-miR-371a-3p | 132.5 | 81.19 | 120.44 | 113.28 | 83.52 | 80.78 | 144.79 | 81.23 | 80.31 | 119.16 |
| hsa-miR-769-5p | 112.87 | 36.54 | 151.06 | 70.61 | 99.73 | 100.97 | 73.39 | 120.34 | 107.92 | 160.87 |
| hsa-let-7d-5p | 137.41 | 60.89 | 132.69 | 86.8 | 63.58 | 115.11 | 109.09 | 73.71 | 115.45 | 135.05 |
| hsa-miR-590-5p | 122.69 | 39.24 | 126.56 | 85.33 | 71.06 | 90.87 | 119.01 | 135.38 | 120.47 | 119.16 |
| hsa-miR-659-3p | 58.89 | 25.71 | 142.89 | 76.5 | 95.99 | 127.22 | 107.11 | 118.84 | 130.51 | 144.98 |
| hsa-miR-566 | 107.96 | 69.01 | 126.56 | 63.26 | 69.81 | 121.16 | 113.06 | 120.34 | 90.35 | 146.97 |
| hsa-miR-28-3p | 137.41 | 47.36 | 128.6 | 70.61 | 79.78 | 107.03 | 99.17 | 97.78 | 125.49 | 133.07 |
| hsa-miR-216b | 107.96 | 73.07 | 118.4 | 79.44 | 103.47 | 105.01 | 117.02 | 123.35 | 100.39 | 97.32 |
| hsa-miR-663a | 117.78 | 44.65 | 128.6 | 73.56 | 59.84 | 107.03 | 126.94 | 100.78 | 138.04 | 125.12 |
| hsa-miR-2117 | 147.22 | 62.25 | 120.44 | 80.91 | 59.84 | 115.11 | 95.21 | 120.34 | 117.96 | 99.3 |
| hsa-miR-548aj-3p | 49.07 | 18.94 | 114.31 | 113.28 | 92.25 | 133.28 | 117.02 | 100.78 | 138.04 | 139.02 |
| hsa-miR-148b-3p | 137.41 | 46.01 | 108.19 | 63.26 | 86.02 | 94.91 | 95.21 | 102.29 | 170.67 | 109.23 |
| hsa-miR-4421 | 220.84 | 79.84 | 93.9 | 52.96 | 74.8 | 123.18 | 89.25 | 79.73 | 105.41 | 87.39 |
| hsa-miR-1 | 171.76 | 31.12 | 146.98 | 70.61 | 51.11 | 109.05 | 105.12 | 109.81 | 100.39 | 111.22 |
| hsa-miR-548a-3p | 63.8 | 29.77 | 110.23 | 69.14 | 82.28 | 139.34 | 138.84 | 112.82 | 117.96 | 135.05 |
| hsa-miR-133b | 152.13 | 52.77 | 97.98 | 80.91 | 66.07 | 117.13 | 103.14 | 108.31 | 128 | 91.36 |
| hsa-miR-33a-5p | 73.61 | 50.07 | 144.93 | 82.38 | 69.81 | 135.3 | 117.02 | 109.81 | 102.9 | 109.23 |
| hsa-miR-23c | 122.69 | 33.83 | 124.52 | 91.21 | 84.77 | 109.05 | 77.35 | 112.82 | 85.33 | 152.93 |
| hsa-miR-1225-5p | 63.8 | 36.54 | 112.27 | 77.97 | 71.06 | 139.34 | 107.11 | 111.31 | 135.53 | 139.02 |
| hsa-miR-337-3p | 117.78 | 44.65 | 128.6 | 88.27 | 87.26 | 80.78 | 109.09 | 130.87 | 110.43 | 95.33 |
| hsa-miR-548aa | 103.06 | 67.66 | 122.48 | 75.03 | 89.75 | 76.74 | 130.91 | 109.81 | 105.41 | 111.22 |
| hsa-miR-644a | 142.32 | 66.31 | 97.98 | 70.61 | 79.78 | 86.83 | 105.12 | 111.31 | 95.37 | 129.09 |
| hsa-miR-4461 | 132.5 | 43.3 | 116.36 | 76.5 | 72.3 | 107.03 | 113.06 | 109.81 | 95.37 | 117.18 |
| hsa-miR-302b-3p | 137.41 | 58.19 | 97.98 | 60.32 | 51.11 | 107.03 | 144.79 | 90.25 | 120.47 | 113.21 |
| hsa-miR-518c-3p | 122.69 | 52.77 | 112.27 | 73.56 | 79.78 | 98.95 | 101.16 | 103.79 | 110.43 | 123.14 |
| hsa-miR-449c-5p | 127.59 | 37.89 | 132.69 | 63.26 | 79.78 | 121.16 | 105.12 | 108.31 | 95.37 | 101.29 |
| hsa-miR-1226-3p | 83.43 | 35.18 | 91.86 | 75.03 | 69.81 | 115.11 | 134.87 | 120.34 | 122.98 | 119.16 |
| hsa-miR-1202 | 166.85 | 63.6 | 124.52 | 52.96 | 72.3 | 94.91 | 113.06 | 103.79 | 75.3 | 99.3 |
| hsa-miR-1273d | 250.28 | 69.01 | 95.94 | 54.43 | 44.88 | 88.85 | 95.21 | 75.21 | 87.84 | 95.33 |
| hsa-miR-1908 | 147.22 | 43.3 | 128.6 | 61.79 | 73.55 | 107.03 | 99.17 | 87.25 | 112.94 | 95.33 |
| hsa-miR-494 | 93.24 | 48.71 | 132.69 | 75.03 | 73.55 | 109.05 | 124.96 | 84.24 | 122.98 | 91.36 |
| hsa-miR-1252 | 122.69 | 32.48 | 106.15 | 60.32 | 71.06 | 125.2 | 111.07 | 96.27 | 105.41 | 117.18 |
| hsa-miR-1197 | 88.33 | 37.89 | 116.36 | 70.61 | 82.28 | 121.16 | 109.09 | 88.75 | 105.41 | 127.11 |
| hsa-miR-876-3p | 157.04 | 35.18 | 114.31 | 70.61 | 72.3 | 109.05 | 91.24 | 82.73 | 110.43 | 103.28 |
| hsa-miR-520h | 103.06 | 59.54 | 89.82 | 75.03 | 88.51 | 115.11 | 113.06 | 103.79 | 72.79 | 125.12 |
| hsa-miR-361-3p | 127.59 | 85.25 | 112.27 | 89.74 | 63.58 | 76.74 | 119.01 | 102.29 | 72.79 | 95.33 |
| hsa-miR-548ai | 127.59 | 98.78 | 91.86 | 63.26 | 89.75 | 78.76 | 134.87 | 94.77 | 72.79 | 91.36 |
| hsa-miR-606 | 73.61 | 28.42 | 130.65 | 60.32 | 74.8 | 92.89 | 109.09 | 112.82 | 138.04 | 119.16 |
| hsa-miR-219-1-3p | 63.8 | 12.18 | 110.23 | 70.61 | 95.99 | 102.99 | 122.97 | 105.3 | 133.02 | 119.16 |
| hsa-miR-4531 | 132.5 | 66.31 | 104.11 | 76.5 | 69.81 | 66.64 | 117.02 | 93.26 | 107.92 | 101.29 |
| hsa-miR-95 | 122.69 | 47.36 | 95.94 | 61.79 | 79.78 | 92.89 | 109.09 | 103.79 | 100.39 | 113.21 |
| hsa-let-7g-5p | 147.22 | 44.65 | 100.03 | 47.08 | 66.07 | 94.91 | 105.12 | 79.73 | 128 | 113.21 |
| hsa-miR-1909-3p | 132.5 | 36.54 | 118.4 | 66.2 | 66.07 | 92.89 | 101.16 | 82.73 | 100.39 | 129.09 |
| hsa-miR-891a | 127.59 | 55.48 | 73.49 | 75.03 | 88.51 | 86.83 | 97.19 | 88.75 | 82.82 | 148.95 |
| hsa-miR-890 | 107.96 | 44.65 | 91.86 | 77.97 | 69.81 | 111.07 | 120.99 | 88.75 | 110.43 | 99.3 |
| hsa-miR-223-3p | 255.19 | 119.08 | 57.16 | 44.13 | 36.15 | 70.68 | 39.67 | 48.14 | 180.71 | 69.51 |
| hsa-miR-647 | 112.87 | 35.18 | 85.74 | 57.37 | 76.04 | 113.09 | 85.29 | 88.75 | 133.02 | 131.08 |
| hsa-miR-3934 | 166.85 | 41.95 | 122.48 | 50.02 | 66.07 | 82.8 | 79.34 | 93.26 | 97.88 | 115.19 |
| hsa-miR-2276 | 83.43 | 56.83 | 95.94 | 63.26 | 56.1 | 111.07 | 120.99 | 115.83 | 102.9 | 107.25 |
| hsa-miR-4458 | 117.78 | 56.83 | 120.44 | 54.43 | 71.06 | 90.87 | 83.3 | 103.79 | 115.45 | 99.3 |
| hsa-miR-325 | 83.43 | 28.42 | 106.15 | 76.5 | 88.51 | 111.07 | 95.21 | 102.29 | 105.41 | 115.19 |
| hsa-miR-574-3p | 107.96 | 44.65 | 112.27 | 55.9 | 69.81 | 127.22 | 95.21 | 96.27 | 97.88 | 101.29 |
| hsa-miR-450a-5p | 107.96 | 47.36 | 116.36 | 58.85 | 62.33 | 88.85 | 95.21 | 93.26 | 120.47 | 117.18 |
| hsa-miR-1257 | 122.69 | 54.13 | 102.07 | 64.73 | 74.8 | 94.91 | 111.07 | 100.78 | 80.31 | 95.33 |
| hsa-miR-200c-3p | 49.07 | 46.01 | 91.86 | 64.73 | 63.58 | 137.32 | 115.04 | 93.26 | 117.96 | 121.15 |
| hsa-miR-658 | 107.96 | 31.12 | 89.82 | 72.09 | 48.62 | 113.09 | 95.21 | 93.26 | 125.49 | 117.18 |
| hsa-miR-301b | 176.67 | 52.77 | 73.49 | 67.67 | 61.08 | 96.93 | 79.34 | 87.25 | 100.39 | 97.32 |
| hsa-miR-938 | 157.04 | 50.07 | 89.82 | 58.85 | 71.06 | 96.93 | 85.29 | 88.75 | 117.96 | 75.47 |
| hsa-miR-550b-3p | 117.78 | 60.89 | 120.44 | 39.72 | 48.62 | 98.95 | 124.96 | 96.27 | 47.69 | 129.09 |
| hsa-miR-1231 | 103.06 | 31.12 | 108.19 | 64.73 | 76.04 | 86.83 | 99.17 | 87.25 | 102.9 | 125.12 |
| hsa-miR-770-5p | 161.95 | 44.65 | 81.65 | 60.32 | 67.32 | 64.62 | 109.09 | 85.74 | 110.43 | 97.32 |
| hsa-miR-424-5p | 98.15 | 27.06 | 100.03 | 67.67 | 68.56 | 107.03 | 97.19 | 106.8 | 107.92 | 101.29 |
| hsa-miR-3182 | 93.24 | 25.71 | 110.23 | 80.91 | 59.84 | 109.05 | 89.25 | 102.29 | 120.47 | 87.39 |
| hsa-miR-548am-3p | 107.96 | 35.18 | 87.78 | 88.27 | 76.04 | 94.91 | 99.17 | 102.29 | 100.39 | 85.4 |
| hsa-miR-635 | 93.24 | 33.83 | 132.69 | 60.32 | 48.62 | 111.07 | 97.19 | 84.24 | 102.9 | 113.21 |
| hsa-miR-455-3p | 44.17 | 24.36 | 134.73 | 82.38 | 69.81 | 121.16 | 95.21 | 100.78 | 100.39 | 103.28 |
| hsa-miR-376a-3p | 44.17 | 48.71 | 104.11 | 72.09 | 107.21 | 84.81 | 120.99 | 108.31 | 87.84 | 97.32 |
| hsa-miR-520b | 132.5 | 69.01 | 118.4 | 73.56 | 62.33 | 70.68 | 99.17 | 81.23 | 80.31 | 87.39 |
| hsa-miR-1288 | 152.13 | 43.3 | 85.74 | 64.73 | 66.07 | 90.87 | 77.35 | 96.27 | 107.92 | 89.37 |
| hsa-miR-320c | 63.8 | 18.94 | 100.03 | 70.61 | 71.06 | 96.93 | 119.01 | 105.3 | 110.43 | 117.18 |
| hsa-miR-605 | 132.5 | 33.83 | 95.94 | 75.03 | 61.08 | 90.87 | 77.35 | 94.77 | 105.41 | 105.26 |
| hsa-miR-563 | 107.96 | 24.36 | 122.48 | 45.61 | 62.33 | 133.28 | 109.09 | 78.22 | 100.39 | 87.39 |
| hsa-miR-1275 | 152.13 | 55.48 | 69.41 | 77.97 | 64.82 | 76.74 | 91.24 | 87.25 | 80.31 | 115.19 |
| hsa-miR-578 | 122.69 | 41.95 | 122.48 | 69.14 | 48.62 | 78.76 | 109.09 | 84.24 | 115.45 | 75.47 |
| hsa-miR-2052 | 103.06 | 18.94 | 95.94 | 66.2 | 47.37 | 94.91 | 103.14 | 87.25 | 115.45 | 135.05 |
| hsa-miR-519e-3p | 98.15 | 39.24 | 81.65 | 70.61 | 67.32 | 109.05 | 124.96 | 88.75 | 87.84 | 99.3 |
| hsa-miR-200a-3p | 127.59 | 78.48 | 108.19 | 61.79 | 62.33 | 70.68 | 89.25 | 88.75 | 87.84 | 91.36 |
| hsa-miR-626 | 68.7 | 32.48 | 102.07 | 57.37 | 83.52 | 113.09 | 103.14 | 108.31 | 95.37 | 99.3 |
| hsa-miR-1208 | 127.59 | 27.06 | 97.98 | 61.79 | 69.81 | 94.91 | 97.19 | 87.25 | 87.84 | 111.22 |
| hsa-miR-212-3p | 112.87 | 41.95 | 81.65 | 76.5 | 52.36 | 88.85 | 89.25 | 112.82 | 95.37 | 107.25 |
| hsa-miR-2277-3p | 142.32 | 37.89 | 100.03 | 57.37 | 57.34 | 88.85 | 81.32 | 100.78 | 112.94 | 77.46 |
| hsa-miR-3175 | 117.78 | 27.06 | 95.94 | 51.49 | 71.06 | 94.91 | 113.06 | 76.72 | 115.45 | 91.36 |
| hsa-miR-759 | 171.76 | 64.95 | 69.41 | 50.02 | 63.58 | 82.8 | 91.24 | 72.2 | 85.33 | 101.29 |
| hsa-miR-586 | 68.7 | 35.18 | 126.56 | 52.96 | 68.56 | 92.89 | 109.09 | 79.73 | 120.47 | 97.32 |
| hsa-miR-330-3p | 147.22 | 46.01 | 59.2 | 48.55 | 62.33 | 107.03 | 97.19 | 82.73 | 90.35 | 109.23 |
| hsa-miR-553 | 166.85 | 43.3 | 61.24 | 67.67 | 71.06 | 102.99 | 83.3 | 100.78 | 80.31 | 71.5 |
| hsa-miR-18a-5p | 83.43 | 40.6 | 87.78 | 54.43 | 54.85 | 119.14 | 93.22 | 82.73 | 112.94 | 119.16 |
| hsa-miR-152 | 137.41 | 46.01 | 73.49 | 52.96 | 51.11 | 82.8 | 93.22 | 90.25 | 120.47 | 99.3 |
| hsa-miR-614 | 78.52 | 41.95 | 104.11 | 58.85 | 69.81 | 102.99 | 73.39 | 85.74 | 122.98 | 103.28 |
| hsa-miR-28-5p | 107.96 | 37.89 | 83.69 | 51.49 | 68.56 | 82.8 | 79.34 | 97.78 | 112.94 | 119.16 |
| hsa-miR-335-5p | 68.7 | 44.65 | 112.27 | 50.02 | 61.08 | 111.07 | 89.25 | 97.78 | 128 | 77.46 |
| hsa-miR-1324 | 201.21 | 47.36 | 79.61 | 69.14 | 39.89 | 80.78 | 73.39 | 72.2 | 87.84 | 87.39 |
| hsa-miR-548ak | 127.59 | 33.83 | 87.78 | 47.08 | 62.33 | 88.85 | 109.09 | 99.28 | 72.79 | 105.26 |
| hsa-miR-367-3p | 171.76 | 31.12 | 71.45 | 42.66 | 52.36 | 100.97 | 122.97 | 81.23 | 65.26 | 93.34 |
| hsa-miR-892b | 147.22 | 28.42 | 93.9 | 54.43 | 74.8 | 62.6 | 87.27 | 97.78 | 110.43 | 75.47 |
| hsa-miR-423-3p | 112.87 | 35.18 | 73.49 | 76.5 | 66.07 | 123.18 | 73.39 | 85.74 | 87.84 | 97.32 |
| hsa-miR-548n | 112.87 | 48.71 | 77.57 | 47.08 | 68.56 | 90.87 | 93.22 | 87.25 | 110.43 | 93.34 |
| hsa-let-7c | 161.95 | 43.3 | 106.15 | 42.66 | 73.55 | 78.76 | 85.29 | 75.21 | 75.3 | 87.39 |
| hsa-miR-604 | 122.69 | 36.54 | 73.49 | 63.26 | 49.86 | 88.85 | 93.22 | 90.25 | 115.45 | 95.33 |
| hsa-miR-769-3p | 166.85 | 41.95 | 95.94 | 47.08 | 73.55 | 88.85 | 63.47 | 76.72 | 85.33 | 85.4 |
| hsa-miR-330-5p | 73.61 | 13.53 | 83.69 | 73.56 | 56.1 | 90.87 | 124.96 | 85.74 | 115.45 | 107.25 |
| hsa-miR-147b | 78.52 | 25.71 | 87.78 | 54.43 | 72.3 | 125.2 | 101.16 | 96.27 | 95.37 | 85.4 |
| hsa-miR-484 | 137.41 | 41.95 | 93.9 | 35.31 | 54.85 | 109.05 | 93.22 | 76.72 | 72.79 | 105.26 |
| hsa-miR-629-5p | 157.04 | 47.36 | 89.82 | 55.9 | 54.85 | 88.85 | 79.34 | 73.71 | 80.31 | 91.36 |
| hsa-miR-320d | 44.17 | 20.3 | 95.94 | 55.9 | 61.08 | 90.87 | 126.94 | 76.72 | 133.02 | 113.21 |
| hsa-miR-23a-3p | 186.48 | 41.95 | 71.45 | 42.66 | 43.63 | 92.89 | 61.49 | 67.69 | 133.02 | 71.5 |
| hsa-miR-193b-3p | 127.59 | 47.36 | 81.65 | 60.32 | 72.3 | 80.78 | 99.17 | 61.67 | 92.86 | 87.39 |
| hsa-miR-4741 | 117.78 | 50.07 | 79.61 | 50.02 | 52.36 | 90.87 | 93.22 | 81.23 | 117.96 | 77.46 |
| hsa-miR-517a-3p | 93.24 | 32.48 | 110.23 | 50.02 | 56.1 | 90.87 | 81.32 | 94.77 | 85.33 | 115.19 |
| hsa-miR-1244 | 147.22 | 47.36 | 75.53 | 57.37 | 64.82 | 68.66 | 87.27 | 84.24 | 75.3 | 101.29 |
| hsa-miR-135b-5p | 107.96 | 29.77 | 104.11 | 69.14 | 68.56 | 86.83 | 77.35 | 87.25 | 100.39 | 75.47 |
| hsa-miR-141-3p | 147.22 | 39.24 | 104.11 | 45.61 | 57.34 | 86.83 | 77.35 | 88.75 | 62.75 | 97.32 |
| hsa-miR-182-5p | 73.61 | 24.36 | 112.27 | 48.55 | 69.81 | 88.85 | 113.06 | 78.22 | 92.86 | 103.28 |
| hsa-miR-891b | 103.06 | 39.24 | 102.07 | 60.32 | 63.58 | 90.87 | 77.35 | 102.29 | 60.24 | 105.26 |
| hsa-miR-762 | 107.96 | 32.48 | 91.86 | 55.9 | 47.37 | 105.01 | 101.16 | 84.24 | 72.79 | 105.26 |
| hsa-miR-581 | 83.43 | 24.36 | 91.86 | 73.56 | 48.62 | 92.89 | 107.11 | 93.26 | 95.37 | 93.34 |
| hsa-miR-3196 | 112.87 | 41.95 | 75.53 | 48.55 | 61.08 | 82.8 | 115.04 | 73.71 | 90.35 | 101.29 |
| hsa-miR-449a | 112.87 | 60.89 | 97.98 | 60.32 | 34.9 | 86.83 | 91.24 | 54.15 | 107.92 | 95.33 |
| hsa-miR-503 | 107.96 | 39.24 | 122.48 | 50.02 | 69.81 | 64.62 | 93.22 | 66.19 | 82.82 | 103.28 |
| hsa-miR-524-3p | 103.06 | 33.83 | 95.94 | 61.79 | 59.84 | 102.99 | 91.24 | 84.24 | 87.84 | 77.46 |
| hsa-miR-329 | 122.69 | 35.18 | 97.98 | 50.02 | 48.62 | 74.72 | 81.32 | 88.75 | 75.3 | 121.15 |
| hsa-miR-513a-5p | 215.93 | 62.25 | 65.32 | 41.19 | 37.4 | 74.72 | 81.32 | 67.69 | 77.81 | 71.5 |
| hsa-miR-323a-3p | 49.07 | 16.24 | 134.73 | 66.2 | 63.58 | 86.83 | 105.12 | 100.78 | 75.3 | 93.34 |
| hsa-miR-1238 | 112.87 | 32.48 | 95.94 | 64.73 | 49.86 | 88.85 | 83.3 | 85.74 | 97.88 | 79.44 |
| hsa-miR-924 | 122.69 | 35.18 | 75.53 | 55.9 | 79.78 | 100.97 | 83.3 | 75.21 | 90.35 | 71.5 |
| hsa-miR-548w | 44.17 | 21.65 | 83.69 | 63.26 | 44.88 | 115.11 | 128.92 | 91.76 | 95.37 | 101.29 |
| hsa-miR-450b-3p | 98.15 | 33.83 | 79.61 | 42.66 | 66.07 | 88.85 | 71.4 | 94.77 | 107.92 | 105.26 |
| hsa-miR-641 | 117.78 | 33.83 | 91.86 | 66.2 | 56.1 | 74.72 | 91.24 | 94.77 | 67.77 | 93.34 |
| hsa-miR-512-5p | 107.96 | 43.3 | 71.45 | 83.85 | 52.36 | 66.64 | 93.22 | 79.73 | 85.33 | 103.28 |
| hsa-miR-99b-5p | 142.32 | 25.71 | 67.36 | 57.37 | 51.11 | 68.66 | 85.29 | 90.25 | 102.9 | 95.33 |
| hsa-miR-1246 | 112.87 | 121.79 | 2.04 | 1.47 | 1.25 | 56.54 | 1.98 | 1.5 | 484.4 | 1.99 |
| hsa-miR-548ae | 93.24 | 20.3 | 112.27 | 58.85 | 49.86 | 80.78 | 95.21 | 108.31 | 75.3 | 91.36 |
| hsa-miR-3690 | 132.5 | 47.36 | 75.53 | 60.32 | 61.08 | 82.8 | 93.22 | 72.2 | 75.3 | 83.41 |
| hsa-miR-432-5p | 117.78 | 36.54 | 87.78 | 58.85 | 64.82 | 76.74 | 79.34 | 78.22 | 97.88 | 85.4 |
| hsa-miR-638 | 88.33 | 32.48 | 69.41 | 42.66 | 81.03 | 92.89 | 111.07 | 88.75 | 85.33 | 91.36 |
| hsa-miR-301a-3p | 161.95 | 47.36 | 61.24 | 42.66 | 62.33 | 70.68 | 71.4 | 67.69 | 90.35 | 107.25 |
| hsa-miR-127-3p | 44.17 | 35.18 | 114.31 | 61.79 | 53.6 | 90.87 | 85.29 | 103.79 | 105.41 | 87.39 |
| hsa-miR-3190-5p | 211.02 | 64.95 | 67.36 | 54.43 | 44.88 | 76.74 | 79.34 | 61.67 | 47.69 | 73.48 |
| hsa-let-7f-5p | 103.06 | 47.36 | 73.49 | 73.56 | 38.64 | 70.68 | 83.3 | 79.73 | 107.92 | 103.28 |
| hsa-miR-1281 | 58.89 | 18.94 | 67.36 | 73.56 | 64.82 | 102.99 | 89.25 | 84.24 | 100.39 | 119.16 |
| hsa-miR-1272 | 107.96 | 31.12 | 104.11 | 55.9 | 58.59 | 98.95 | 89.25 | 78.22 | 67.77 | 87.39 |
| hsa-miR-191-5p | 127.59 | 36.54 | 79.61 | 57.37 | 76.04 | 82.8 | 89.25 | 76.72 | 67.77 | 85.4 |
| hsa-miR-211-5p | 304.26 | 56.83 | 44.91 | 45.61 | 33.66 | 56.54 | 55.54 | 46.63 | 60.24 | 73.48 |
| hsa-miR-502-3p | 132.5 | 31.12 | 63.28 | 51.49 | 51.11 | 92.89 | 87.27 | 81.23 | 82.82 | 103.28 |
| hsa-miR-548c-5p | 142.32 | 51.42 | 87.78 | 48.55 | 49.86 | 58.56 | 122.97 | 61.67 | 67.77 | 85.4 |
| hsa-miR-511 | 137.41 | 41.95 | 59.2 | 63.26 | 72.3 | 70.68 | 71.4 | 82.73 | 87.84 | 89.37 |
| hsa-let-7b-5p | 137.41 | 44.65 | 75.53 | 44.13 | 52.36 | 90.87 | 93.22 | 82.73 | 62.75 | 91.36 |
| hsa-miR-208a | 68.7 | 25.71 | 112.27 | 69.14 | 57.34 | 82.8 | 81.32 | 88.75 | 85.33 | 103.28 |
| hsa-miR-885-5p | 73.61 | 25.71 | 100.03 | 58.85 | 63.58 | 78.76 | 85.29 | 81.23 | 95.37 | 111.22 |
| hsa-miR-1303 | 68.7 | 17.59 | 79.61 | 61.79 | 58.59 | 60.58 | 97.19 | 94.77 | 115.45 | 119.16 |
| hsa-miR-3168 | 107.96 | 35.18 | 93.9 | 52.96 | 58.59 | 80.78 | 83.3 | 78.22 | 100.39 | 79.44 |
| hsa-miR-3136-5p | 117.78 | 40.6 | 83.69 | 63.26 | 69.81 | 86.83 | 75.37 | 88.75 | 80.31 | 63.55 |
| hsa-miR-342-5p | 44.17 | 24.36 | 59.2 | 76.5 | 63.58 | 100.97 | 109.09 | 115.83 | 82.82 | 93.34 |
| hsa-miR-30b-5p | 63.8 | 28.42 | 85.74 | 64.73 | 52.36 | 100.97 | 103.14 | 100.78 | 90.35 | 79.44 |
| hsa-miR-3928 | 112.87 | 37.89 | 87.78 | 48.55 | 44.88 | 84.81 | 81.32 | 82.73 | 85.33 | 103.28 |
| hsa-miR-1538 | 142.32 | 56.83 | 89.82 | 48.55 | 52.36 | 76.74 | 95.21 | 63.18 | 70.28 | 73.48 |
| hsa-miR-16-5p | 63.8 | 58.19 | 91.86 | 54.43 | 63.58 | 74.72 | 99.17 | 82.73 | 47.69 | 131.08 |
| hsa-miR-532-5p | 107.96 | 31.12 | 87.78 | 48.55 | 52.36 | 96.93 | 81.32 | 73.71 | 112.94 | 73.48 |
| hsa-miR-1205 | 98.15 | 39.24 | 89.82 | 47.08 | 72.3 | 82.8 | 97.19 | 81.23 | 80.31 | 77.46 |
| hsa-miR-153 | 132.5 | 32.48 | 91.86 | 48.55 | 52.36 | 88.85 | 85.29 | 66.19 | 95.37 | 71.5 |
| hsa-miR-499a-5p | 142.32 | 52.77 | 61.24 | 55.9 | 57.34 | 58.56 | 53.55 | 88.75 | 92.86 | 101.29 |
| hsa-miR-485-5p | 112.87 | 46.01 | 87.78 | 50.02 | 59.84 | 72.7 | 89.25 | 81.23 | 95.37 | 69.51 |
| hsa-miR-7-5p | 103.06 | 35.18 | 104.11 | 66.2 | 36.15 | 80.78 | 63.47 | 93.26 | 92.86 | 89.37 |
| hsa-miR-1255b-5p | 29.44 | 31.12 | 110.23 | 60.32 | 71.06 | 70.68 | 95.21 | 106.8 | 77.81 | 111.22 |
| hsa-miR-142-3p | 98.15 | 75.78 | 83.69 | 51.49 | 58.59 | 68.66 | 71.4 | 91.76 | 92.86 | 71.5 |
| hsa-miR-1298 | 176.67 | 58.19 | 57.16 | 58.85 | 39.89 | 68.66 | 79.34 | 60.17 | 95.37 | 69.51 |
| hsa-miR-520d-5p+hsa-miR-518a-5p+hsa-miR-527 | 83.43 | 77.13 | 77.57 | 77.97 | 73.55 | 42.41 | 105.12 | 96.27 | 32.63 | 97.32 |
| hsa-miR-34a-5p | 88.33 | 25.71 | 81.65 | 50.02 | 62.33 | 84.81 | 97.19 | 78.22 | 90.35 | 103.28 |
| hsa-miR-382-5p | 220.84 | 44.65 | 36.74 | 48.55 | 52.36 | 76.74 | 85.29 | 58.67 | 72.79 | 63.55 |
| hsa-miR-1255a | 98.15 | 24.36 | 95.94 | 48.55 | 51.11 | 98.95 | 91.24 | 67.69 | 85.33 | 97.32 |
| hsa-miR-3605-5p | 127.59 | 43.3 | 112.27 | 58.85 | 61.08 | 82.8 | 85.29 | 67.69 | 62.75 | 55.61 |
| hsa-miR-146a-5p | 73.61 | 36.54 | 106.15 | 51.49 | 58.59 | 70.68 | 93.22 | 90.25 | 90.35 | 85.4 |
| hsa-miR-22-3p | 98.15 | 43.3 | 75.53 | 51.49 | 48.62 | 105.01 | 87.27 | 58.67 | 110.43 | 77.46 |
| hsa-miR-219-5p | 73.61 | 35.18 | 97.98 | 60.32 | 52.36 | 84.81 | 73.39 | 87.25 | 67.77 | 123.14 |
| hsa-miR-195-5p | 88.33 | 27.06 | 91.86 | 61.79 | 44.88 | 88.85 | 91.24 | 72.2 | 97.88 | 85.4 |
| hsa-miR-504 | 206.11 | 64.95 | 59.2 | 35.31 | 51.11 | 70.68 | 59.5 | 60.17 | 77.81 | 63.55 |
| hsa-miR-1973 | 83.43 | 37.89 | 89.82 | 72.09 | 54.85 | 80.78 | 105.12 | 51.14 | 87.84 | 83.41 |
| hsa-miR-1276 | 107.96 | 36.54 | 81.65 | 58.85 | 61.08 | 76.74 | 57.52 | 66.19 | 100.39 | 99.3 |
| hsa-miR-516a-3p | 98.15 | 32.48 | 102.07 | 63.26 | 59.84 | 78.76 | 67.44 | 66.19 | 107.92 | 69.51 |
| hsa-miR-1251 | 63.8 | 29.77 | 110.23 | 67.67 | 54.85 | 82.8 | 73.39 | 82.73 | 115.45 | 63.55 |
| hsa-miR-1825 | 127.59 | 41.95 | 71.45 | 52.96 | 56.1 | 90.87 | 83.3 | 67.69 | 80.31 | 71.5 |
| hsa-miR-383 | 142.32 | 35.18 | 73.49 | 45.61 | 44.88 | 70.68 | 75.37 | 91.76 | 97.88 | 65.54 |
| hsa-miR-1200 | 93.24 | 21.65 | 69.41 | 67.67 | 61.08 | 88.85 | 79.34 | 84.24 | 77.81 | 99.3 |
| hsa-miR-615-5p | 93.24 | 32.48 | 65.32 | 51.49 | 54.85 | 96.93 | 73.39 | 70.7 | 95.37 | 107.25 |
| hsa-miR-129-2-3p | 107.96 | 31.12 | 89.82 | 61.79 | 49.86 | 109.05 | 89.25 | 52.65 | 77.81 | 71.5 |
| hsa-miR-933 | 83.43 | 29.77 | 95.94 | 47.08 | 49.86 | 98.95 | 79.34 | 87.25 | 67.77 | 101.29 |
| hsa-miR-15b-5p | 73.61 | 29.77 | 81.65 | 60.32 | 53.6 | 72.7 | 85.29 | 72.2 | 125.49 | 85.4 |
| hsa-miR-151a-5p | 88.33 | 28.42 | 75.53 | 55.9 | 47.37 | 92.89 | 91.24 | 69.2 | 77.81 | 111.22 |
| hsa-miR-1233 | 93.24 | 33.83 | 67.36 | 44.13 | 59.84 | 58.56 | 91.24 | 85.74 | 117.96 | 85.4 |
| hsa-miR-1539 | 147.22 | 35.18 | 83.69 | 47.08 | 46.12 | 70.68 | 81.32 | 64.68 | 77.81 | 83.41 |
| hsa-miR-34c-5p | 93.24 | 27.06 | 91.86 | 57.37 | 51.11 | 72.7 | 91.24 | 75.21 | 82.82 | 93.34 |
| hsa-miR-124-3p | 206.11 | 50.07 | 55.12 | 39.72 | 37.4 | 66.64 | 73.39 | 46.63 | 87.84 | 71.5 |
| hsa-miR-922 | 122.69 | 50.07 | 83.69 | 64.73 | 47.37 | 62.6 | 53.55 | 88.75 | 90.35 | 69.51 |
| hsa-let-7i-5p | 93.24 | 31.12 | 87.78 | 55.9 | 44.88 | 96.93 | 81.32 | 75.21 | 85.33 | 81.43 |
| hsa-miR-939 | 132.5 | 41.95 | 83.69 | 58.85 | 53.6 | 68.66 | 67.44 | 69.2 | 72.79 | 83.41 |
| hsa-miR-1250 | 58.89 | 29.77 | 79.61 | 51.49 | 52.36 | 105.01 | 93.22 | 90.25 | 80.31 | 87.39 |
| hsa-miR-519d | 58.89 | 39.24 | 104.11 | 47.08 | 61.08 | 70.68 | 101.16 | 103.79 | 47.69 | 93.34 |
| hsa-miR-1234 | 93.24 | 46.01 | 71.45 | 47.08 | 67.32 | 76.74 | 77.35 | 66.19 | 80.31 | 101.29 |
| hsa-miR-670 | 132.5 | 39.24 | 65.32 | 48.55 | 59.84 | 68.66 | 99.17 | 58.67 | 75.3 | 79.44 |
| hsa-miR-302a-3p | 83.43 | 28.42 | 91.86 | 60.32 | 46.12 | 84.81 | 97.19 | 81.23 | 77.81 | 75.47 |
| hsa-miR-409-3p | 98.15 | 23 | 102.07 | 52.96 | 48.62 | 88.85 | 69.42 | 84.24 | 65.26 | 93.34 |
| hsa-miR-29c-3p | 132.5 | 35.18 | 57.16 | 67.67 | 41.14 | 86.83 | 89.25 | 76.72 | 62.75 | 75.47 |
| hsa-miR-4488 | 137.41 | 43.3 | 61.24 | 45.61 | 44.88 | 62.6 | 91.24 | 73.71 | 72.79 | 89.37 |
| hsa-miR-411-5p | 147.22 | 47.36 | 73.49 | 39.72 | 54.85 | 52.5 | 85.29 | 63.18 | 70.28 | 87.39 |
| hsa-miR-500a-5p+hsa-miR-501-5p | 73.61 | 40.6 | 106.15 | 52.96 | 66.07 | 64.62 | 63.47 | 94.77 | 57.73 | 101.29 |
| hsa-miR-3184-5p | 93.24 | 24.36 | 79.61 | 51.49 | 56.1 | 62.6 | 83.3 | 99.28 | 72.79 | 97.32 |
| hsa-miR-3141 | 103.06 | 29.77 | 83.69 | 51.49 | 57.34 | 96.93 | 97.19 | 72.2 | 52.71 | 75.47 |
| hsa-miR-142-5p | 112.87 | 31.12 | 93.9 | 44.13 | 61.08 | 60.58 | 71.4 | 85.74 | 95.37 | 63.55 |
| hsa-miR-205-5p | 107.96 | 28.42 | 46.95 | 50.02 | 63.58 | 102.99 | 79.34 | 90.25 | 85.33 | 63.55 |
| hsa-miR-15a-5p | 78.52 | 31.12 | 69.41 | 44.13 | 41.14 | 107.03 | 95.21 | 70.7 | 75.3 | 105.26 |
| hsa-miR-1299 | 112.87 | 28.42 | 118.4 | 58.85 | 66.07 | 68.66 | 69.42 | 69.2 | 62.75 | 61.57 |
| hsa-miR-526a+hsa-miR-520c-5p+hsa-miR-518d-5p | 98.15 | 48.71 | 108.19 | 35.31 | 42.38 | 70.68 | 83.3 | 70.7 | 82.82 | 75.47 |
| hsa-miR-196a-5p | 88.33 | 47.36 | 97.98 | 54.43 | 54.85 | 60.58 | 71.4 | 82.73 | 80.31 | 77.46 |
| hsa-miR-214-3p | 44.17 | 27.06 | 81.65 | 55.9 | 63.58 | 94.91 | 73.39 | 91.76 | 67.77 | 115.19 |
| hsa-miR-548g-3p | 103.06 | 43.3 | 59.2 | 73.56 | 53.6 | 84.81 | 67.44 | 55.66 | 72.79 | 101.29 |
| hsa-miR-93-5p | 112.87 | 51.42 | 100.03 | 45.61 | 44.88 | 70.68 | 83.3 | 54.15 | 75.3 | 75.47 |
| hsa-miR-942 | 53.98 | 25.71 | 97.98 | 60.32 | 62.33 | 62.6 | 95.21 | 84.24 | 67.77 | 103.28 |
| hsa-miR-941 | 73.61 | 31.12 | 65.32 | 55.9 | 57.34 | 92.89 | 79.34 | 67.69 | 87.84 | 101.29 |
| hsa-miR-564 | 44.17 | 13.53 | 89.82 | 64.73 | 57.34 | 94.91 | 83.3 | 72.2 | 80.31 | 111.22 |
| hsa-miR-196b-5p | 98.15 | 40.6 | 83.69 | 45.61 | 59.84 | 68.66 | 79.34 | 79.73 | 80.31 | 75.47 |
| hsa-miR-585 | 93.24 | 40.6 | 77.57 | 52.96 | 69.81 | 84.81 | 59.5 | 69.2 | 77.81 | 85.4 |
| hsa-miR-200b-3p | 53.98 | 31.12 | 91.86 | 50.02 | 48.62 | 84.81 | 95.21 | 78.22 | 75.3 | 101.29 |
| hsa-miR-422a | 122.69 | 35.18 | 89.82 | 44.13 | 44.88 | 76.74 | 81.32 | 63.18 | 70.28 | 81.43 |
| hsa-miR-657 | 83.43 | 27.06 | 100.03 | 55.9 | 47.37 | 76.74 | 89.25 | 72.2 | 70.28 | 85.4 |
| hsa-miR-548an | 58.89 | 23 | 89.82 | 47.08 | 61.08 | 78.76 | 57.52 | 99.28 | 90.35 | 101.29 |
| hsa-miR-622 | 78.52 | 31.12 | 89.82 | 57.37 | 59.84 | 100.97 | 71.4 | 85.74 | 60.24 | 71.5 |
| hsa-miR-1322 | 63.8 | 36.54 | 77.57 | 57.37 | 58.59 | 70.68 | 79.34 | 82.73 | 72.79 | 105.26 |
| hsa-miR-937 | 103.06 | 32.48 | 85.74 | 47.08 | 53.6 | 94.91 | 61.49 | 84.24 | 75.3 | 63.55 |
| hsa-miR-431-5p | 88.33 | 27.06 | 83.69 | 66.2 | 59.84 | 96.93 | 65.45 | 64.68 | 75.3 | 73.48 |
| hsa-miR-502-5p | 98.15 | 17.59 | 89.82 | 41.19 | 58.59 | 82.8 | 89.25 | 76.72 | 75.3 | 71.5 |
| hsa-miR-1266 | 107.96 | 25.71 | 73.49 | 54.43 | 48.62 | 78.76 | 95.21 | 60.17 | 72.79 | 83.41 |
| hsa-miR-542-3p | 157.04 | 55.48 | 106.15 | 54.43 | 48.62 | 42.41 | 51.57 | 78.22 | 42.67 | 63.55 |
| hsa-miR-665 | 44.17 | 23 | 100.03 | 70.61 | 47.37 | 92.89 | 73.39 | 84.24 | 60.24 | 103.28 |
| hsa-miR-596 | 122.69 | 48.71 | 67.36 | 54.43 | 51.11 | 50.48 | 77.35 | 43.62 | 97.88 | 85.4 |
| hsa-miR-26a-5p | 147.22 | 48.71 | 75.53 | 41.19 | 39.89 | 60.58 | 63.47 | 82.73 | 75.3 | 63.55 |
| hsa-miR-516b-5p | 68.7 | 44.65 | 57.16 | 70.61 | 51.11 | 82.8 | 91.24 | 70.7 | 67.77 | 93.34 |
| hsa-miR-320a | 117.78 | 50.07 | 79.61 | 39.72 | 48.62 | 58.56 | 73.39 | 79.73 | 72.79 | 77.46 |
| hsa-miR-203 | 83.43 | 31.12 | 85.74 | 57.37 | 57.34 | 68.66 | 87.27 | 90.25 | 72.79 | 63.55 |
| hsa-miR-936 | 98.15 | 23 | 83.69 | 39.72 | 48.62 | 94.91 | 87.27 | 57.16 | 72.79 | 89.37 |
| hsa-miR-515-3p | 107.96 | 27.06 | 73.49 | 54.43 | 47.37 | 76.74 | 67.44 | 82.73 | 87.84 | 69.51 |
| hsa-miR-507 | 53.98 | 33.83 | 75.53 | 48.55 | 53.6 | 88.85 | 75.37 | 78.22 | 87.84 | 97.32 |
| hsa-miR-3147 | 137.41 | 31.12 | 65.32 | 45.61 | 42.38 | 78.76 | 65.45 | 69.2 | 80.31 | 77.46 |
| hsa-miR-140-5p | 107.96 | 25.71 | 75.53 | 44.13 | 53.6 | 80.78 | 83.3 | 84.24 | 62.75 | 73.48 |
| hsa-miR-96-5p | 103.06 | 28.42 | 63.28 | 45.61 | 42.38 | 88.85 | 63.47 | 69.2 | 115.45 | 71.5 |
| hsa-miR-29a-3p | 58.89 | 31.12 | 69.41 | 51.49 | 66.07 | 86.83 | 83.3 | 72.2 | 67.77 | 103.28 |
| hsa-miR-4431 | 78.52 | 27.06 | 79.61 | 35.31 | 64.82 | 70.68 | 103.14 | 78.22 | 85.33 | 67.53 |
| hsa-miR-524-5p | 93.24 | 23 | 95.94 | 42.66 | 56.1 | 90.87 | 55.54 | 79.73 | 77.81 | 73.48 |
| hsa-miR-3180 | 83.43 | 35.18 | 87.78 | 47.08 | 41.14 | 117.13 | 69.42 | 70.7 | 80.31 | 53.62 |
| hsa-miR-151a-3p | 127.59 | 24.36 | 77.57 | 33.84 | 51.11 | 82.8 | 61.49 | 67.69 | 75.3 | 83.41 |
| hsa-miR-410 | 107.96 | 24.36 | 100.03 | 44.13 | 39.89 | 76.74 | 89.25 | 69.2 | 70.28 | 59.58 |
| hsa-miR-216a | 112.87 | 35.18 | 97.98 | 51.49 | 53.6 | 72.7 | 59.5 | 54.15 | 70.28 | 73.48 |
| hsa-miR-548q | 49.07 | 46.01 | 120.44 | 58.85 | 81.03 | 32.31 | 79.34 | 99.28 | 45.18 | 69.51 |
| hsa-miR-571 | 63.8 | 27.06 | 67.36 | 50.02 | 53.6 | 56.54 | 115.04 | 88.75 | 75.3 | 83.41 |
| hsa-miR-520f | 83.43 | 36.54 | 69.41 | 44.13 | 47.37 | 80.78 | 69.42 | 106.8 | 57.73 | 83.41 |
| hsa-miR-199a-3p+hsa-miR-199b-3p | 93.24 | 29.77 | 75.53 | 52.96 | 56.1 | 48.47 | 89.25 | 75.21 | 82.82 | 75.47 |
| hsa-miR-520c-3p | 137.41 | 31.12 | 69.41 | 38.25 | 42.38 | 76.74 | 53.55 | 55.66 | 97.88 | 75.47 |
| hsa-miR-218-5p | 58.89 | 33.83 | 79.61 | 60.32 | 43.63 | 78.76 | 73.39 | 67.69 | 100.39 | 79.44 |
| hsa-miR-643 | 68.7 | 32.48 | 83.69 | 29.42 | 49.86 | 76.74 | 87.27 | 63.18 | 65.26 | 119.16 |
| hsa-miR-548a-5p | 68.7 | 39.24 | 85.74 | 41.19 | 47.37 | 64.62 | 99.17 | 97.78 | 60.24 | 71.5 |
| hsa-miR-105-5p | 127.59 | 27.06 | 61.24 | 44.13 | 39.89 | 68.66 | 55.54 | 72.2 | 90.35 | 87.39 |
| hsa-miR-496 | 53.98 | 52.77 | 87.78 | 44.13 | 69.81 | 64.62 | 83.3 | 87.25 | 57.73 | 71.5 |
| hsa-let-7a-5p | 58.89 | 63.6 | 77.57 | 54.43 | 46.12 | 74.72 | 85.29 | 64.68 | 87.84 | 59.58 |
| hsa-miR-1307-3p | 83.43 | 21.65 | 63.28 | 45.61 | 49.86 | 82.8 | 67.44 | 76.72 | 70.28 | 111.22 |
| hsa-miR-3131 | 83.43 | 25.71 | 61.24 | 63.26 | 52.36 | 80.78 | 59.5 | 66.19 | 107.92 | 71.5 |
| hsa-miR-548d-5p | 122.69 | 21.65 | 67.36 | 44.13 | 47.37 | 58.56 | 45.62 | 64.68 | 138.04 | 61.57 |
| hsa-miR-1228-3p | 117.78 | 46.01 | 67.36 | 35.31 | 43.63 | 74.72 | 83.3 | 60.17 | 67.77 | 75.47 |
| hsa-miR-718 | 63.8 | 23 | 81.65 | 50.02 | 56.1 | 90.87 | 87.27 | 66.19 | 72.79 | 79.44 |
| hsa-miR-518a-3p | 103.06 | 29.77 | 91.86 | 29.42 | 41.14 | 74.72 | 77.35 | 70.7 | 75.3 | 77.46 |
| hsa-miR-575 | 137.41 | 40.6 | 36.74 | 41.19 | 53.6 | 80.78 | 87.27 | 46.63 | 72.79 | 73.48 |
| hsa-miR-1258 | 93.24 | 31.12 | 69.41 | 47.08 | 47.37 | 86.83 | 105.12 | 64.68 | 67.77 | 57.6 |
| hsa-miR-1178 | 78.52 | 29.77 | 89.82 | 41.19 | 48.62 | 94.91 | 67.44 | 67.69 | 90.35 | 61.57 |
| hsa-miR-551a | 98.15 | 27.06 | 79.61 | 48.55 | 68.56 | 70.68 | 63.47 | 60.17 | 60.24 | 91.36 |
| hsa-miR-522-3p | 83.43 | 27.06 | 61.24 | 45.61 | 49.86 | 72.7 | 61.49 | 78.22 | 80.31 | 107.25 |
| hsa-miR-331-3p | 58.89 | 39.24 | 87.78 | 52.96 | 51.11 | 78.76 | 93.22 | 66.19 | 70.28 | 67.53 |
| hsa-miR-487a | 73.61 | 43.3 | 69.41 | 51.49 | 64.82 | 66.64 | 69.42 | 76.72 | 72.79 | 77.46 |
| hsa-miR-20a-5p+hsa-miR-20b-5p | 63.8 | 24.36 | 67.36 | 44.13 | 43.63 | 88.85 | 73.39 | 70.7 | 82.82 | 105.26 |
| hsa-miR-651 | 39.26 | 28.42 | 89.82 | 51.49 | 56.1 | 68.66 | 67.44 | 84.24 | 85.33 | 93.34 |
| hsa-miR-512-3p | 103.06 | 29.77 | 77.57 | 36.78 | 41.14 | 56.54 | 85.29 | 69.2 | 82.82 | 81.43 |
| hsa-miR-139-3p | 93.24 | 37.89 | 89.82 | 38.25 | 53.6 | 66.64 | 87.27 | 61.67 | 65.26 | 69.51 |
| hsa-miR-185-5p | 39.26 | 21.65 | 93.9 | 54.43 | 56.1 | 70.68 | 89.25 | 79.73 | 62.75 | 95.33 |
| hsa-miR-3187-3p | 53.98 | 29.77 | 91.86 | 50.02 | 63.58 | 72.7 | 59.5 | 64.68 | 105.41 | 71.5 |
| hsa-miR-219-2-3p | 73.61 | 21.65 | 104.11 | 57.37 | 38.64 | 72.7 | 73.39 | 69.2 | 72.79 | 79.44 |
| hsa-miR-633 | 98.15 | 24.36 | 93.9 | 44.13 | 44.88 | 68.66 | 71.4 | 79.73 | 67.77 | 69.51 |
| hsa-miR-429 | 58.89 | 46.01 | 79.61 | 54.43 | 42.38 | 66.64 | 65.45 | 72.2 | 85.33 | 91.36 |
| hsa-miR-378b | 83.43 | 33.83 | 89.82 | 50.02 | 49.86 | 58.56 | 67.44 | 79.73 | 57.73 | 91.36 |
| hsa-miR-661 | 68.7 | 29.77 | 63.28 | 48.55 | 62.33 | 72.7 | 95.21 | 96.27 | 65.26 | 59.58 |
| hsa-miR-600 | 44.17 | 24.36 | 93.9 | 48.55 | 42.38 | 82.8 | 111.07 | 49.64 | 75.3 | 89.37 |
| hsa-miR-378a-3p+hsa-miR-378i | 83.43 | 28.42 | 91.86 | 44.13 | 73.55 | 76.74 | 65.45 | 78.22 | 50.2 | 69.51 |
| hsa-miR-548t-5p | 78.52 | 23 | 108.19 | 50.02 | 39.89 | 52.5 | 71.4 | 69.2 | 75.3 | 93.34 |
| hsa-miR-558 | 112.87 | 28.42 | 65.32 | 58.85 | 41.14 | 94.91 | 75.37 | 48.14 | 67.77 | 67.53 |
| hsa-miR-621 | 88.33 | 36.54 | 63.28 | 50.02 | 54.85 | 70.68 | 59.5 | 52.65 | 90.35 | 93.34 |
| hsa-miR-548l | 122.69 | 32.48 | 77.57 | 39.72 | 49.86 | 60.58 | 69.42 | 49.64 | 87.84 | 69.51 |
| hsa-miR-513a-3p | 107.96 | 51.42 | 61.24 | 33.84 | 54.85 | 66.64 | 69.42 | 79.73 | 70.28 | 63.55 |
| hsa-miR-1245b-5p | 98.15 | 46.01 | 69.41 | 52.96 | 58.59 | 66.64 | 67.44 | 66.19 | 72.79 | 59.58 |
| hsa-miR-1291 | 78.52 | 40.6 | 89.82 | 48.55 | 52.36 | 66.64 | 83.3 | 58.67 | 65.26 | 71.5 |
| hsa-miR-556-5p | 235.56 | 67.66 | 57.16 | 27.95 | 26.18 | 52.5 | 41.65 | 43.62 | 42.67 | 59.58 |
| hsa-miR-1204 | 88.33 | 37.89 | 87.78 | 50.02 | 32.41 | 72.7 | 51.57 | 72.2 | 67.77 | 93.34 |
| hsa-miR-519c-3p | 98.15 | 40.6 | 63.28 | 42.66 | 33.66 | 88.85 | 71.4 | 84.24 | 62.75 | 65.54 |
| hsa-miR-593-3p | 78.52 | 23 | 73.49 | 38.25 | 46.12 | 82.8 | 59.5 | 67.69 | 90.35 | 91.36 |
| hsa-miR-518e-3p | 44.17 | 24.36 | 67.36 | 61.79 | 44.88 | 92.89 | 55.54 | 85.74 | 92.86 | 81.43 |
| hsa-miR-1263 | 19.63 | 24.36 | 73.49 | 57.37 | 48.62 | 80.78 | 85.29 | 84.24 | 80.31 | 95.33 |
| hsa-miR-599 | 63.8 | 21.65 | 53.07 | 50.02 | 47.37 | 80.78 | 81.32 | 69.2 | 80.31 | 101.29 |
| hsa-miR-320b | 78.52 | 21.65 | 46.95 | 38.25 | 34.9 | 84.81 | 77.35 | 81.23 | 75.3 | 109.23 |
| hsa-miR-628-3p | 68.7 | 21.65 | 79.61 | 64.73 | 58.59 | 68.66 | 63.47 | 58.67 | 90.35 | 73.48 |
| hsa-miR-767-3p | 58.89 | 40.6 | 87.78 | 38.25 | 48.62 | 70.68 | 89.25 | 82.73 | 65.26 | 65.54 |
| hsa-miR-372 | 93.24 | 31.12 | 55.12 | 42.66 | 39.89 | 72.7 | 89.25 | 67.69 | 67.77 | 87.39 |
| hsa-miR-767-5p | 88.33 | 23 | 85.74 | 45.61 | 41.14 | 74.72 | 85.29 | 52.65 | 87.84 | 61.57 |
| hsa-miR-302f | 112.87 | 44.65 | 83.69 | 39.72 | 44.88 | 62.6 | 65.45 | 45.13 | 80.31 | 65.54 |
| hsa-miR-3178 | 107.96 | 27.06 | 55.12 | 45.61 | 49.86 | 72.7 | 85.29 | 61.67 | 65.26 | 73.48 |
| hsa-miR-550a-5p | 49.07 | 35.18 | 75.53 | 47.08 | 76.04 | 64.62 | 59.5 | 85.74 | 75.3 | 75.47 |
| hsa-miR-569 | 122.69 | 32.48 | 67.36 | 39.72 | 44.88 | 64.62 | 49.59 | 69.2 | 85.33 | 67.53 |
| hsa-miR-766-3p | 88.33 | 32.48 | 77.57 | 29.42 | 39.89 | 80.78 | 73.39 | 79.73 | 70.28 | 69.51 |
| hsa-miR-1236 | 68.7 | 21.65 | 73.49 | 42.66 | 48.62 | 90.87 | 73.39 | 69.2 | 72.79 | 79.44 |
| hsa-miR-107 | 44.17 | 31.12 | 87.78 | 50.02 | 61.08 | 64.62 | 67.44 | 76.72 | 70.28 | 87.39 |
| hsa-miR-548i | 98.15 | 24.36 | 85.74 | 41.19 | 48.62 | 54.52 | 73.39 | 64.68 | 92.86 | 55.61 |
| hsa-miR-2114-5p | 152.13 | 29.77 | 59.2 | 38.25 | 41.14 | 66.64 | 67.44 | 49.64 | 65.26 | 69.51 |
| hsa-miR-3180-5p | 53.98 | 18.94 | 95.94 | 55.9 | 53.6 | 82.8 | 59.5 | 70.7 | 77.81 | 69.51 |
| hsa-miR-525-3p | 73.61 | 23 | 71.45 | 48.55 | 53.6 | 78.76 | 77.35 | 69.2 | 65.26 | 77.46 |
| hsa-miR-654-5p | 63.8 | 24.36 | 91.86 | 45.61 | 48.62 | 52.5 | 75.37 | 73.71 | 80.31 | 81.43 |
| hsa-miR-9-5p | 68.7 | 41.95 | 79.61 | 48.55 | 56.1 | 66.64 | 53.55 | 67.69 | 100.39 | 53.62 |
| hsa-miR-625-5p | 127.59 | 35.18 | 89.82 | 39.72 | 42.38 | 46.45 | 79.34 | 69.2 | 45.18 | 61.57 |
| hsa-miR-587 | 83.43 | 36.54 | 95.94 | 48.55 | 58.59 | 50.48 | 69.42 | 55.66 | 65.26 | 71.5 |
| hsa-miR-1268a | 93.24 | 37.89 | 61.24 | 44.13 | 44.88 | 68.66 | 95.21 | 49.64 | 75.3 | 63.55 |
| hsa-miR-1181 | 58.89 | 31.12 | 65.32 | 36.78 | 37.4 | 78.76 | 87.27 | 64.68 | 77.81 | 95.33 |
| hsa-miR-663b | 83.43 | 40.6 | 93.9 | 36.78 | 58.59 | 64.62 | 49.59 | 57.16 | 72.79 | 75.47 |
| hsa-miR-671-5p | 88.33 | 23 | 87.78 | 39.72 | 47.37 | 76.74 | 73.39 | 51.14 | 65.26 | 79.44 |
| hsa-miR-378f | 132.5 | 39.24 | 51.03 | 41.19 | 38.64 | 60.58 | 69.42 | 42.12 | 77.81 | 79.44 |
| hsa-miR-126-3p | 49.07 | 35.18 | 65.32 | 69.14 | 37.4 | 74.72 | 83.3 | 81.23 | 50.2 | 85.4 |
| hsa-miR-590-3p | 157.04 | 31.12 | 59.2 | 38.25 | 31.16 | 64.62 | 53.55 | 60.17 | 70.28 | 65.54 |
| hsa-miR-337-5p | 132.5 | 33.83 | 65.32 | 35.31 | 39.89 | 54.52 | 67.44 | 70.7 | 77.81 | 51.64 |
| hsa-miR-559 | 44.17 | 21.65 | 65.32 | 48.55 | 46.12 | 82.8 | 67.44 | 91.76 | 72.79 | 87.39 |
| hsa-miR-421 | 117.78 | 36.54 | 53.07 | 39.72 | 42.38 | 82.8 | 81.32 | 52.65 | 60.24 | 59.58 |
| hsa-miR-561-3p | 53.98 | 17.59 | 91.86 | 50.02 | 47.37 | 70.68 | 61.49 | 57.16 | 100.39 | 75.47 |
| hsa-miR-557 | 63.8 | 35.18 | 89.82 | 39.72 | 31.16 | 80.78 | 77.35 | 81.23 | 75.3 | 51.64 |
| hsa-miR-198 | 98.15 | 24.36 | 81.65 | 54.43 | 41.14 | 66.64 | 65.45 | 63.18 | 75.3 | 55.61 |
| hsa-miR-210 | 93.24 | 29.77 | 71.45 | 32.36 | 51.11 | 82.8 | 69.42 | 60.17 | 57.73 | 77.46 |
| hsa-miR-1273e | 93.24 | 33.83 | 67.36 | 42.66 | 58.59 | 64.62 | 65.45 | 61.67 | 60.24 | 77.46 |
| hsa-miR-1273a | 166.85 | 46.01 | 46.95 | 42.66 | 33.66 | 58.56 | 51.57 | 52.65 | 60.24 | 65.54 |
| hsa-miR-134 | 132.5 | 17.59 | 69.41 | 41.19 | 38.64 | 72.7 | 65.45 | 61.67 | 47.69 | 75.47 |
| hsa-miR-27a-3p | 103.06 | 27.06 | 71.45 | 44.13 | 47.37 | 58.56 | 83.3 | 51.14 | 62.75 | 73.48 |
| hsa-miR-106b-5p | 73.61 | 24.36 | 77.57 | 39.72 | 49.86 | 48.47 | 87.27 | 88.75 | 55.22 | 77.46 |
| hsa-miR-552 | 107.96 | 32.48 | 46.95 | 45.61 | 32.41 | 74.72 | 57.52 | 69.2 | 87.84 | 67.53 |
| hsa-miR-34c-3p | 93.24 | 31.12 | 71.45 | 55.9 | 38.64 | 84.81 | 71.4 | 49.64 | 60.24 | 65.54 |
| hsa-miR-3123 | 44.17 | 32.48 | 91.86 | 44.13 | 47.37 | 72.7 | 63.47 | 63.18 | 72.79 | 89.37 |
| hsa-miR-943 | 73.61 | 18.94 | 93.9 | 41.19 | 39.89 | 66.64 | 65.45 | 69.2 | 70.28 | 81.43 |
| hsa-miR-221-3p | 73.61 | 33.83 | 81.65 | 52.96 | 37.4 | 76.74 | 87.27 | 57.16 | 60.24 | 59.58 |
| hsa-miR-1256 | 53.98 | 18.94 | 65.32 | 36.78 | 43.63 | 84.81 | 87.27 | 81.23 | 92.86 | 55.61 |
| hsa-miR-508-5p | 53.98 | 21.65 | 83.69 | 50.02 | 49.86 | 76.74 | 81.32 | 66.19 | 65.26 | 71.5 |
| hsa-miR-510 | 112.87 | 39.24 | 81.65 | 35.31 | 42.38 | 62.6 | 51.57 | 43.62 | 82.82 | 67.53 |
| hsa-miR-32-5p | 127.59 | 32.48 | 69.41 | 48.55 | 47.37 | 66.64 | 57.52 | 57.16 | 52.71 | 59.58 |
| hsa-miR-139-5p | 107.96 | 20.3 | 81.65 | 27.95 | 48.62 | 56.54 | 77.35 | 54.15 | 70.28 | 73.48 |
| hsa-miR-485-3p | 83.43 | 27.06 | 75.53 | 50.02 | 46.12 | 68.66 | 63.47 | 64.68 | 75.3 | 63.55 |
| hsa-miR-1305 | 127.59 | 132.61 | 48.99 | 25.01 | 68.56 | 46.45 | 51.57 | 42.12 | 42.67 | 31.78 |
| hsa-miR-3614-5p | 98.15 | 37.89 | 55.12 | 41.19 | 43.63 | 38.37 | 83.3 | 52.65 | 82.82 | 83.41 |
| hsa-miR-548y | 112.87 | 32.48 | 57.16 | 45.61 | 62.33 | 68.66 | 35.7 | 75.21 | 42.67 | 83.41 |
| hsa-miR-323b-3p | 78.52 | 29.77 | 51.03 | 38.25 | 51.11 | 86.83 | 73.39 | 45.13 | 80.31 | 81.43 |
| hsa-miR-1470 | 98.15 | 39.24 | 63.28 | 36.78 | 57.34 | 58.56 | 67.44 | 42.12 | 82.82 | 69.51 |
| hsa-miR-2278 | 93.24 | 27.06 | 48.99 | 47.08 | 37.4 | 70.68 | 69.42 | 61.67 | 70.28 | 89.37 |
| hsa-miR-1206 | 93.24 | 31.12 | 63.28 | 52.96 | 29.92 | 72.7 | 65.45 | 67.69 | 45.18 | 93.34 |
| hsa-miR-1468 | 93.24 | 14.88 | 73.49 | 54.43 | 36.15 | 60.58 | 67.44 | 69.2 | 77.81 | 67.53 |
| hsa-miR-506-3p | 68.7 | 21.65 | 69.41 | 35.31 | 47.37 | 60.58 | 83.3 | 75.21 | 67.77 | 85.4 |
| hsa-miR-610 | 49.07 | 14.88 | 69.41 | 48.55 | 53.6 | 88.85 | 93.22 | 64.68 | 72.79 | 59.58 |
| hsa-miR-100-5p | 98.15 | 24.36 | 67.36 | 48.55 | 46.12 | 68.66 | 69.42 | 64.68 | 75.3 | 51.64 |
| hsa-miR-562 | 103.06 | 29.77 | 81.65 | 33.84 | 44.88 | 66.64 | 77.35 | 55.66 | 55.22 | 65.54 |
| hsa-miR-875-3p | 53.98 | 20.3 | 73.49 | 57.37 | 51.11 | 78.76 | 97.19 | 64.68 | 62.75 | 53.62 |
| hsa-miR-488-3p | 53.98 | 25.71 | 61.24 | 47.08 | 53.6 | 90.87 | 81.32 | 52.65 | 80.31 | 65.54 |
| hsa-let-7e-5p | 58.89 | 39.24 | 65.32 | 45.61 | 48.62 | 70.68 | 47.6 | 76.72 | 85.33 | 73.48 |
| hsa-miR-374a-5p | 103.06 | 28.42 | 79.61 | 36.78 | 44.88 | 60.58 | 53.55 | 57.16 | 72.79 | 73.48 |
| hsa-miR-4532 | 93.24 | 36.54 | 63.28 | 60.32 | 43.63 | 66.64 | 71.4 | 54.15 | 50.2 | 69.51 |
| hsa-miR-1282 | 112.87 | 23 | 55.12 | 38.25 | 42.38 | 60.58 | 59.5 | 64.68 | 90.35 | 61.57 |
| hsa-miR-1193 | 73.61 | 28.42 | 53.07 | 38.25 | 39.89 | 62.6 | 71.4 | 67.69 | 87.84 | 85.4 |
| hsa-miR-764 | 83.43 | 29.77 | 61.24 | 45.61 | 46.12 | 68.66 | 77.35 | 49.64 | 72.79 | 73.48 |
| hsa-miR-517c-3p+hsa-miR-519a-3p | 122.69 | 50.07 | 59.2 | 29.42 | 38.64 | 64.62 | 83.3 | 51.14 | 65.26 | 43.69 |
| hsa-miR-1321 | 132.5 | 39.24 | 59.2 | 39.72 | 53.6 | 48.47 | 71.4 | 55.66 | 40.16 | 67.53 |
| hsa-miR-1254 | 78.52 | 13.53 | 59.2 | 38.25 | 34.9 | 70.68 | 87.27 | 67.69 | 87.84 | 69.51 |
| hsa-miR-135a-5p | 68.7 | 28.42 | 65.32 | 39.72 | 53.6 | 68.66 | 71.4 | 52.65 | 82.82 | 75.47 |
| hsa-miR-346 | 63.8 | 40.6 | 67.36 | 38.25 | 42.38 | 64.62 | 81.32 | 82.73 | 67.77 | 57.6 |
| hsa-miR-877-5p | 49.07 | 24.36 | 79.61 | 35.31 | 62.33 | 72.7 | 91.24 | 60.17 | 57.73 | 73.48 |
| hsa-miR-188-3p | 83.43 | 27.06 | 65.32 | 45.61 | 36.15 | 70.68 | 83.3 | 61.67 | 57.73 | 73.48 |
| hsa-miR-1270 | 171.76 | 55.48 | 55.12 | 44.13 | 26.18 | 36.35 | 63.47 | 54.15 | 40.16 | 57.6 |
| hsa-miR-3127-5p | 142.32 | 29.77 | 65.32 | 38.25 | 36.15 | 56.54 | 57.52 | 58.67 | 60.24 | 59.58 |
| hsa-miR-145-5p | 117.78 | 21.65 | 79.61 | 33.84 | 42.38 | 66.64 | 65.45 | 64.68 | 57.73 | 53.62 |
| hsa-miR-650 | 73.61 | 31.12 | 63.28 | 48.55 | 42.38 | 48.47 | 79.34 | 58.67 | 80.31 | 77.46 |
| hsa-miR-1248 | 58.89 | 29.77 | 73.49 | 51.49 | 48.62 | 90.87 | 61.49 | 54.15 | 72.79 | 61.57 |
| hsa-miR-648 | 63.8 | 27.06 | 75.53 | 41.19 | 54.85 | 80.78 | 63.47 | 66.19 | 70.28 | 59.58 |
| hsa-miR-885-3p | 98.15 | 27.06 | 51.03 | 58.85 | 52.36 | 66.64 | 63.47 | 43.62 | 77.81 | 63.55 |
| hsa-miR-1302 | 83.43 | 24.36 | 63.28 | 38.25 | 43.63 | 72.7 | 61.49 | 54.15 | 75.3 | 85.4 |
| hsa-miR-4647 | 83.43 | 35.18 | 73.49 | 44.13 | 28.67 | 62.6 | 71.4 | 67.69 | 75.3 | 59.58 |
| hsa-miR-541-3p | 103.06 | 33.83 | 75.53 | 35.31 | 36.15 | 68.66 | 53.55 | 73.71 | 67.77 | 53.62 |
| hsa-miR-498 | 58.89 | 31.12 | 71.45 | 48.55 | 42.38 | 58.56 | 77.35 | 70.7 | 47.69 | 93.34 |
| hsa-miR-409-5p | 58.89 | 39.24 | 71.45 | 38.25 | 51.11 | 66.64 | 67.44 | 70.7 | 62.75 | 73.48 |
| hsa-miR-376c | 68.7 | 40.6 | 63.28 | 55.9 | 49.86 | 72.7 | 55.54 | 37.61 | 70.28 | 85.4 |
| hsa-miR-378c | 53.98 | 20.3 | 59.2 | 42.66 | 42.38 | 86.83 | 99.17 | 49.64 | 67.77 | 77.46 |
| hsa-miR-548m | 98.15 | 41.95 | 53.07 | 41.19 | 63.58 | 60.58 | 65.45 | 67.69 | 40.16 | 67.53 |
| hsa-miR-548ab | 34.35 | 29.77 | 75.53 | 54.43 | 56.1 | 44.43 | 71.4 | 55.66 | 87.84 | 89.37 |
| hsa-miR-525-5p | 93.24 | 47.36 | 85.74 | 39.72 | 34.9 | 58.56 | 65.45 | 40.61 | 57.73 | 75.47 |
| hsa-miR-1271-5p | 78.52 | 32.48 | 65.32 | 35.31 | 44.88 | 72.7 | 57.52 | 64.68 | 70.28 | 75.47 |
| hsa-miR-624-3p | 73.61 | 32.48 | 87.78 | 35.31 | 48.62 | 58.56 | 63.47 | 58.67 | 55.22 | 83.41 |
| hsa-miR-520a-5p | 93.24 | 24.36 | 53.07 | 54.43 | 49.86 | 74.72 | 67.44 | 67.69 | 42.67 | 69.51 |
| hsa-miR-567 | 117.78 | 36.54 | 63.28 | 39.72 | 37.4 | 42.41 | 73.39 | 51.14 | 65.26 | 69.51 |
| hsa-miR-23b-3p | 107.96 | 25.71 | 63.28 | 42.66 | 32.41 | 66.64 | 65.45 | 45.13 | 70.28 | 75.47 |
| hsa-miR-4284 | 78.52 | 31.12 | 59.2 | 45.61 | 48.62 | 58.56 | 63.47 | 79.73 | 47.69 | 81.43 |
| hsa-miR-519b-3p | 201.21 | 54.13 | 42.87 | 33.84 | 36.15 | 50.48 | 45.62 | 27.08 | 55.22 | 45.68 |
| hsa-miR-744-5p | 83.43 | 39.24 | 69.41 | 36.78 | 37.4 | 78.76 | 51.57 | 57.16 | 52.71 | 83.41 |
| hsa-miR-577 | 83.43 | 23 | 75.53 | 38.25 | 48.62 | 62.6 | 49.59 | 60.17 | 72.79 | 75.47 |
| hsa-miR-193a-3p | 117.78 | 36.54 | 59.2 | 29.42 | 36.15 | 50.48 | 61.49 | 64.68 | 67.77 | 65.54 |
| hsa-miR-514b-3p | 78.52 | 21.65 | 53.07 | 57.37 | 32.41 | 72.7 | 77.35 | 69.2 | 55.22 | 71.5 |
| hsa-miR-634 | 73.61 | 28.42 | 91.86 | 39.72 | 46.12 | 76.74 | 57.52 | 58.67 | 60.24 | 55.61 |
| hsa-miR-197-3p | 147.22 | 46.01 | 73.49 | 35.31 | 29.92 | 40.39 | 51.57 | 51.14 | 75.3 | 37.74 |
| hsa-miR-215 | 73.61 | 40.6 | 89.82 | 41.19 | 39.89 | 72.7 | 51.57 | 58.67 | 70.28 | 49.65 |
| hsa-miR-4435 | 88.33 | 28.42 | 65.32 | 38.25 | 44.88 | 50.48 | 65.45 | 45.13 | 97.88 | 63.55 |
| hsa-miR-632 | 63.8 | 20.3 | 63.28 | 41.19 | 38.64 | 70.68 | 83.3 | 61.67 | 82.82 | 61.57 |
| hsa-miR-1537 | 83.43 | 32.48 | 42.87 | 32.36 | 48.62 | 82.8 | 59.5 | 57.16 | 90.35 | 57.6 |
| hsa-miR-140-3p | 63.8 | 14.88 | 63.28 | 45.61 | 42.38 | 82.8 | 63.47 | 55.66 | 77.81 | 77.46 |
| hsa-miR-183-5p | 73.61 | 35.18 | 79.61 | 30.89 | 41.14 | 58.56 | 71.4 | 42.12 | 75.3 | 77.46 |
| hsa-miR-324-5p | 127.59 | 27.06 | 61.24 | 35.31 | 38.64 | 66.64 | 59.5 | 64.68 | 52.71 | 51.64 |
| hsa-miR-1287 | 49.07 | 29.77 | 77.57 | 51.49 | 47.37 | 60.58 | 63.47 | 70.7 | 62.75 | 71.5 |
| hsa-miR-613 | 63.8 | 47.36 | 53.07 | 63.26 | 46.12 | 68.66 | 53.55 | 54.15 | 75.3 | 57.6 |
| hsa-miR-758 | 107.96 | 28.42 | 75.53 | 44.13 | 49.86 | 58.56 | 63.47 | 42.12 | 52.71 | 59.58 |
| hsa-miR-589-5p | 93.24 | 33.83 | 77.57 | 51.49 | 36.15 | 56.54 | 45.62 | 49.64 | 52.71 | 85.4 |
| hsa-miR-331-5p | 107.96 | 32.48 | 81.65 | 39.72 | 37.4 | 82.8 | 57.52 | 49.64 | 55.22 | 37.74 |
| hsa-miR-603 | 58.89 | 48.71 | 83.69 | 30.89 | 46.12 | 56.54 | 51.57 | 87.25 | 62.75 | 55.61 |
| hsa-miR-224-5p | 83.43 | 23 | 63.28 | 44.13 | 39.89 | 82.8 | 61.49 | 52.65 | 62.75 | 67.53 |
| hsa-miR-889 | 112.87 | 20.3 | 75.53 | 32.36 | 43.63 | 46.45 | 59.5 | 52.65 | 77.81 | 59.58 |
| hsa-miR-655 | 147.22 | 51.42 | 36.74 | 48.55 | 46.12 | 24.23 | 81.32 | 49.64 | 45.18 | 49.65 |
| hsa-miR-137 | 147.22 | 29.77 | 51.03 | 45.61 | 46.12 | 66.64 | 37.69 | 58.67 | 45.18 | 51.64 |
| hsa-miR-4448 | 83.43 | 13.53 | 53.07 | 29.42 | 38.64 | 80.78 | 71.4 | 69.2 | 57.73 | 81.43 |
| hsa-miR-544b | 49.07 | 13.53 | 57.16 | 25.01 | 52.36 | 80.78 | 71.4 | 52.65 | 72.79 | 103.28 |
| hsa-miR-376b | 112.87 | 41.95 | 57.16 | 42.66 | 37.4 | 44.43 | 53.55 | 51.14 | 82.82 | 53.62 |
| hsa-miR-1264 | 112.87 | 37.89 | 36.74 | 38.25 | 36.15 | 70.68 | 75.37 | 51.14 | 52.71 | 65.54 |
| hsa-miR-548j | 49.07 | 20.3 | 69.41 | 44.13 | 54.85 | 80.78 | 69.42 | 69.2 | 50.2 | 69.51 |
| hsa-miR-487b | 73.61 | 31.12 | 73.49 | 42.66 | 41.14 | 54.52 | 73.39 | 67.69 | 72.79 | 45.68 |
| hsa-miR-369-3p | 53.98 | 29.77 | 79.61 | 42.66 | 53.6 | 64.62 | 51.57 | 54.15 | 70.28 | 75.47 |
| hsa-miR-339-3p | 73.61 | 36.54 | 67.36 | 36.78 | 43.63 | 50.48 | 53.55 | 57.16 | 67.77 | 87.39 |
| hsa-miR-3200-3p | 215.93 | 69.01 | 44.91 | 20.6 | 18.7 | 40.39 | 41.65 | 25.57 | 45.18 | 51.64 |
| hsa-miR-199a-5p | 58.89 | 37.89 | 51.03 | 52.96 | 58.59 | 34.33 | 73.39 | 72.2 | 42.67 | 91.36 |
| hsa-miR-208b | 122.69 | 28.42 | 71.45 | 39.72 | 22.44 | 58.56 | 57.52 | 54.15 | 70.28 | 47.67 |
| hsa-miR-548v | 68.7 | 14.88 | 77.57 | 38.25 | 28.67 | 68.66 | 67.44 | 81.23 | 57.73 | 69.51 |
| hsa-miR-106a-5p+hsa-miR-17-5p | 68.7 | 33.83 | 77.57 | 48.55 | 36.15 | 62.6 | 59.5 | 57.16 | 70.28 | 57.6 |
| hsa-miR-1284 | 73.61 | 25.71 | 69.41 | 32.36 | 39.89 | 46.45 | 85.29 | 57.16 | 57.73 | 83.41 |
| hsa-miR-1237 | 63.8 | 28.42 | 63.28 | 51.49 | 47.37 | 60.58 | 71.4 | 66.19 | 52.71 | 65.54 |
| hsa-miR-132-3p | 93.24 | 28.42 | 79.61 | 35.31 | 37.4 | 52.5 | 55.54 | 67.69 | 65.26 | 55.61 |
| hsa-miR-548c-3p | 58.89 | 23 | 61.24 | 36.78 | 41.14 | 60.58 | 51.57 | 81.23 | 57.73 | 97.32 |
| hsa-miR-548s | 152.13 | 23 | 44.91 | 38.25 | 34.9 | 58.56 | 47.6 | 55.66 | 50.2 | 63.55 |
| hsa-miR-1279 | 83.43 | 36.54 | 69.41 | 36.78 | 51.11 | 56.54 | 55.54 | 58.67 | 55.22 | 63.55 |
| hsa-miR-345-5p | 73.61 | 32.48 | 65.32 | 33.84 | 47.37 | 76.74 | 69.42 | 40.61 | 57.73 | 69.51 |
| hsa-miR-4286 | 39.26 | 39.24 | 77.57 | 36.78 | 56.1 | 64.62 | 67.44 | 73.71 | 40.16 | 71.5 |
| hsa-miR-592 | 78.52 | 35.18 | 77.57 | 39.72 | 52.36 | 54.52 | 49.59 | 39.11 | 67.77 | 71.5 |
| hsa-miR-3164 | 34.35 | 21.65 | 95.94 | 41.19 | 36.15 | 76.74 | 53.55 | 66.19 | 70.28 | 69.51 |
| hsa-miR-653 | 112.87 | 24.36 | 57.16 | 41.19 | 41.14 | 32.31 | 67.44 | 70.7 | 52.71 | 65.54 |
| hsa-miR-765 | 117.78 | 21.65 | 51.03 | 35.31 | 36.15 | 54.52 | 55.54 | 60.17 | 57.73 | 73.48 |
| hsa-miR-548o-3p | 63.8 | 35.18 | 55.12 | 39.72 | 37.4 | 88.85 | 53.55 | 58.67 | 52.71 | 77.46 |
| hsa-miR-454-3p | 44.17 | 18.94 | 59.2 | 38.25 | 49.86 | 60.58 | 77.35 | 82.73 | 52.71 | 77.46 |
| hsa-miR-583 | 98.15 | 33.83 | 53.07 | 45.61 | 41.14 | 58.56 | 61.49 | 51.14 | 52.71 | 65.54 |
| hsa-miR-202-3p | 58.89 | 16.24 | 77.57 | 33.84 | 48.62 | 64.62 | 63.47 | 63.18 | 65.26 | 69.51 |
| hsa-miR-521 | 88.33 | 23 | 46.95 | 32.36 | 34.9 | 58.56 | 55.54 | 55.66 | 85.33 | 79.44 |
| hsa-miR-31-5p | 88.33 | 39.24 | 38.79 | 32.36 | 36.15 | 60.58 | 51.57 | 43.62 | 87.84 | 81.43 |
| hsa-miR-374b-5p | 107.96 | 36.54 | 61.24 | 35.31 | 31.16 | 58.56 | 47.6 | 70.7 | 52.71 | 57.6 |
| hsa-miR-875-5p | 98.15 | 25.71 | 57.16 | 36.78 | 52.36 | 52.5 | 49.59 | 75.21 | 50.2 | 61.57 |
| hsa-miR-639 | 88.33 | 31.12 | 77.57 | 33.84 | 47.37 | 62.6 | 41.65 | 48.14 | 70.28 | 57.6 |
| hsa-miR-3676-3p | 73.61 | 37.89 | 61.24 | 26.48 | 37.4 | 62.6 | 73.39 | 54.15 | 62.75 | 67.53 |
| hsa-miR-371b-5p | 73.61 | 32.48 | 65.32 | 44.13 | 46.12 | 72.7 | 47.6 | 42.12 | 75.3 | 55.61 |
| hsa-miR-548h-5p | 73.61 | 24.36 | 75.53 | 36.78 | 33.66 | 48.47 | 77.35 | 43.62 | 85.33 | 55.61 |
| hsa-miR-1203 | 49.07 | 14.88 | 83.69 | 45.61 | 49.86 | 60.58 | 57.52 | 73.71 | 55.22 | 63.55 |
| hsa-miR-27b-3p | 73.61 | 21.65 | 57.16 | 42.66 | 33.66 | 64.62 | 71.4 | 60.17 | 60.24 | 67.53 |
| hsa-miR-588 | 83.43 | 29.77 | 44.91 | 38.25 | 33.66 | 66.64 | 57.52 | 49.64 | 70.28 | 77.46 |
| hsa-miR-26b-5p | 98.15 | 31.12 | 51.03 | 47.08 | 37.4 | 50.48 | 51.57 | 61.67 | 75.3 | 47.67 |
| hsa-miR-1304-5p | 63.8 | 13.53 | 46.95 | 45.61 | 53.6 | 58.56 | 79.34 | 61.67 | 62.75 | 65.54 |
| hsa-miR-660-5p | 53.98 | 23 | 81.65 | 42.66 | 42.38 | 60.58 | 65.45 | 64.68 | 60.24 | 55.61 |
| hsa-miR-483-3p | 98.15 | 17.59 | 67.36 | 51.49 | 37.4 | 54.52 | 57.52 | 52.65 | 47.69 | 65.54 |
| hsa-miR-125a-3p | 83.43 | 24.36 | 69.41 | 41.19 | 54.85 | 36.35 | 63.47 | 49.64 | 45.18 | 81.43 |
| hsa-miR-451a | 83.43 | 25.71 | 51.03 | 36.78 | 32.41 | 50.48 | 77.35 | 67.69 | 72.79 | 51.64 |
| hsa-miR-1292 | 107.96 | 25.71 | 55.12 | 36.78 | 41.14 | 54.52 | 61.49 | 70.7 | 57.73 | 37.74 |
| hsa-miR-491-5p | 49.07 | 16.24 | 75.53 | 48.55 | 46.12 | 72.7 | 49.59 | 36.1 | 65.26 | 89.37 |
| hsa-miR-380-3p | 93.24 | 43.3 | 51.03 | 36.78 | 41.14 | 54.52 | 63.47 | 46.63 | 47.69 | 69.51 |
| hsa-miR-711 | 93.24 | 23 | 59.2 | 47.08 | 48.62 | 60.58 | 41.65 | 57.16 | 62.75 | 53.62 |
| hsa-miR-3161 | 53.98 | 23 | 57.16 | 30.89 | 52.36 | 44.43 | 65.45 | 82.73 | 62.75 | 73.48 |
| hsa-miR-545-3p | 49.07 | 27.06 | 63.28 | 42.66 | 48.62 | 60.58 | 69.42 | 45.13 | 72.79 | 67.53 |
| hsa-miR-1180 | 98.15 | 41.95 | 55.12 | 35.31 | 24.93 | 56.54 | 91.24 | 51.14 | 25.1 | 65.54 |
| hsa-miR-576-3p | 112.87 | 35.18 | 83.69 | 38.25 | 28.67 | 54.52 | 43.64 | 48.14 | 50.2 | 49.65 |
| hsa-miR-1289 | 68.7 | 13.53 | 69.41 | 36.78 | 46.12 | 60.58 | 79.34 | 46.63 | 67.77 | 55.61 |
| hsa-miR-101-3p | 44.17 | 27.06 | 75.53 | 32.36 | 39.89 | 72.7 | 61.49 | 64.68 | 55.22 | 69.51 |
| hsa-miR-892a | 98.15 | 18.94 | 89.82 | 41.19 | 56.1 | 70.68 | 43.64 | 51.14 | 35.14 | 37.74 |
| hsa-miR-369-5p | 73.61 | 18.94 | 67.36 | 26.48 | 38.64 | 58.56 | 77.35 | 52.65 | 62.75 | 65.54 |
| hsa-miR-338-5p | 44.17 | 21.65 | 67.36 | 47.08 | 41.14 | 44.43 | 75.37 | 63.18 | 77.81 | 59.58 |
| hsa-miR-520a-3p | 73.61 | 25.71 | 51.03 | 38.25 | 51.11 | 62.6 | 59.5 | 63.18 | 52.71 | 63.55 |
| hsa-miR-10b-5p | 53.98 | 23 | 67.36 | 51.49 | 27.43 | 78.76 | 57.52 | 54.15 | 52.71 | 73.48 |
| hsa-miR-520d-3p | 73.61 | 16.24 | 53.07 | 33.84 | 39.89 | 78.76 | 49.59 | 61.67 | 65.26 | 67.53 |
| hsa-miR-187-3p | 68.7 | 20.3 | 61.24 | 47.08 | 33.66 | 62.6 | 57.52 | 60.17 | 60.24 | 67.53 |
| hsa-miR-371b-3p | 68.7 | 16.24 | 63.28 | 48.55 | 38.64 | 76.74 | 67.44 | 63.18 | 40.16 | 55.61 |
| hsa-miR-298 | 34.35 | 10.83 | 65.32 | 45.61 | 41.14 | 56.54 | 59.5 | 58.67 | 85.33 | 79.44 |
| hsa-miR-554 | 78.52 | 29.77 | 79.61 | 30.89 | 47.37 | 44.43 | 53.55 | 58.67 | 60.24 | 53.62 |
| hsa-miR-381 | 73.61 | 37.89 | 48.99 | 36.78 | 38.64 | 54.52 | 69.42 | 52.65 | 45.18 | 77.46 |
| hsa-miR-668 | 78.52 | 23 | 61.24 | 39.72 | 41.14 | 42.41 | 63.47 | 54.15 | 52.71 | 77.46 |
| hsa-miR-492 | 73.61 | 14.88 | 71.45 | 39.72 | 29.92 | 62.6 | 57.52 | 57.16 | 80.31 | 45.68 |
| hsa-miR-194-5p | 58.89 | 25.71 | 44.91 | 35.31 | 41.14 | 78.76 | 65.45 | 60.17 | 72.79 | 49.65 |
| hsa-miR-33b-5p | 58.89 | 24.36 | 55.12 | 35.31 | 49.86 | 58.56 | 59.5 | 69.2 | 70.28 | 51.64 |
| hsa-miR-514a-3p | 98.15 | 24.36 | 59.2 | 47.08 | 41.14 | 52.5 | 35.7 | 48.14 | 72.79 | 53.62 |
| hsa-miR-486-3p | 49.07 | 32.48 | 53.07 | 36.78 | 38.64 | 76.74 | 59.5 | 55.66 | 72.79 | 57.6 |
| hsa-miR-646 | 78.52 | 18.94 | 69.41 | 32.36 | 46.12 | 56.54 | 55.54 | 48.14 | 62.75 | 63.55 |
| hsa-miR-452-5p | 58.89 | 14.88 | 42.87 | 60.32 | 46.12 | 48.47 | 55.54 | 55.66 | 75.3 | 73.48 |
| hsa-miR-675-5p | 166.85 | 43.3 | 38.79 | 23.54 | 28.67 | 42.41 | 43.64 | 54.15 | 37.65 | 51.64 |
| hsa-miR-944 | 49.07 | 32.48 | 83.69 | 51.49 | 36.15 | 46.45 | 57.52 | 57.16 | 55.22 | 59.58 |
| hsa-miR-548b-3p | 58.89 | 13.53 | 73.49 | 44.13 | 37.4 | 54.52 | 61.49 | 48.14 | 55.22 | 81.43 |
| hsa-miR-2110 | 73.61 | 18.94 | 63.28 | 41.19 | 27.43 | 62.6 | 49.59 | 45.13 | 72.79 | 73.48 |
| hsa-miR-4451 | 63.8 | 29.77 | 40.83 | 48.55 | 36.15 | 64.62 | 43.64 | 51.14 | 75.3 | 73.48 |
| hsa-miR-493-3p | 53.98 | 29.77 | 67.36 | 36.78 | 39.89 | 72.7 | 37.69 | 51.14 | 60.24 | 77.46 |
| hsa-miR-934 | 73.61 | 13.53 | 55.12 | 38.25 | 31.16 | 58.56 | 45.62 | 52.65 | 77.81 | 79.44 |
| hsa-miR-1224-5p | 83.43 | 35.18 | 55.12 | 45.61 | 34.9 | 56.54 | 51.57 | 61.67 | 37.65 | 63.55 |
| hsa-miR-1297 | 83.43 | 32.48 | 48.99 | 29.42 | 32.41 | 50.48 | 61.49 | 61.67 | 62.75 | 61.57 |
| hsa-miR-19b-3p | 63.8 | 18.94 | 57.16 | 25.01 | 38.64 | 60.58 | 63.47 | 70.7 | 57.73 | 65.54 |
| hsa-miR-720 | 49.07 | 27.06 | 53.07 | 35.31 | 69.81 | 54.52 | 39.67 | 52.65 | 85.33 | 53.62 |
| hsa-miR-935 | 107.96 | 47.36 | 51.03 | 27.95 | 27.43 | 48.47 | 59.5 | 48.14 | 50.2 | 51.64 |
| hsa-miR-190a | 112.87 | 28.42 | 53.07 | 26.48 | 37.4 | 46.45 | 55.54 | 49.64 | 30.12 | 79.44 |
| hsa-miR-2113 | 78.52 | 21.65 | 73.49 | 33.84 | 29.92 | 36.35 | 85.29 | 51.14 | 60.24 | 47.67 |
| hsa-miR-34b-3p | 83.43 | 21.65 | 63.28 | 35.31 | 39.89 | 52.5 | 47.6 | 48.14 | 60.24 | 65.54 |
| hsa-miR-340-5p | 98.15 | 63.6 | 51.03 | 30.89 | 39.89 | 52.5 | 43.64 | 40.61 | 37.65 | 59.58 |
| hsa-miR-607 | 44.17 | 16.24 | 63.28 | 44.13 | 33.66 | 60.58 | 69.42 | 61.67 | 45.18 | 77.46 |
| hsa-miR-582-3p | 117.78 | 27.06 | 59.2 | 16.18 | 34.9 | 56.54 | 37.69 | 40.61 | 77.81 | 45.68 |
| hsa-miR-548ag | 103.06 | 17.59 | 46.95 | 32.36 | 44.88 | 54.52 | 49.59 | 58.67 | 45.18 | 59.58 |
| hsa-miR-483-5p | 93.24 | 32.48 | 53.07 | 29.42 | 44.88 | 50.48 | 47.6 | 64.68 | 42.67 | 53.62 |
| hsa-miR-1471 | 73.61 | 32.48 | 38.79 | 48.55 | 34.9 | 52.5 | 65.45 | 49.64 | 50.2 | 65.54 |
| hsa-miR-499b-5p | 78.52 | 18.94 | 57.16 | 36.78 | 33.66 | 60.58 | 63.47 | 51.14 | 57.73 | 53.62 |
| hsa-miR-300 | 103.06 | 20.3 | 51.03 | 32.36 | 43.63 | 50.48 | 45.62 | 39.11 | 62.75 | 59.58 |
| hsa-miR-573 | 103.06 | 32.48 | 44.91 | 42.66 | 29.92 | 36.35 | 79.34 | 49.64 | 45.18 | 43.69 |
| hsa-miR-1262 | 98.15 | 29.77 | 63.28 | 35.31 | 34.9 | 48.47 | 57.52 | 48.14 | 47.69 | 43.69 |
| hsa-miR-608 | 73.61 | 21.65 | 63.28 | 30.89 | 36.15 | 48.47 | 65.45 | 52.65 | 52.71 | 61.57 |
| hsa-miR-548e | 58.89 | 24.36 | 46.95 | 36.78 | 38.64 | 36.35 | 61.49 | 73.71 | 65.26 | 63.55 |
| hsa-miR-1911-5p | 83.43 | 27.06 | 46.95 | 39.72 | 46.12 | 52.5 | 47.6 | 46.63 | 50.2 | 65.54 |
| hsa-miR-1273g-5p | 63.8 | 27.06 | 42.87 | 32.36 | 38.64 | 50.48 | 39.67 | 61.67 | 72.79 | 75.47 |
| hsa-miR-576-5p | 49.07 | 16.24 | 67.36 | 30.89 | 54.85 | 54.52 | 47.6 | 46.63 | 67.77 | 67.53 |
| hsa-miR-146b-3p | 39.26 | 31.12 | 48.99 | 45.61 | 47.37 | 44.43 | 53.55 | 66.19 | 57.73 | 67.53 |
| hsa-miR-760 | 98.15 | 25.71 | 65.32 | 41.19 | 32.41 | 48.47 | 57.52 | 45.13 | 30.12 | 57.6 |
| hsa-miR-328 | 73.61 | 29.77 | 51.03 | 27.95 | 47.37 | 50.48 | 61.49 | 43.62 | 47.69 | 67.53 |
| hsa-miR-542-5p | 88.33 | 16.24 | 48.99 | 26.48 | 28.67 | 46.45 | 55.54 | 63.18 | 45.18 | 81.43 |
| hsa-miR-1290 | 49.07 | 33.83 | 46.95 | 58.85 | 37.4 | 48.47 | 73.39 | 54.15 | 42.67 | 55.61 |
| hsa-miR-30a-5p | 107.96 | 29.77 | 51.03 | 29.42 | 24.93 | 50.48 | 71.4 | 58.67 | 30.12 | 45.68 |
| hsa-miR-3151 | 49.07 | 25.71 | 48.99 | 45.61 | 29.92 | 62.6 | 49.59 | 46.63 | 82.82 | 57.6 |
| hsa-miR-591 | 68.7 | 24.36 | 42.87 | 39.72 | 33.66 | 68.66 | 71.4 | 54.15 | 35.14 | 59.58 |
| hsa-miR-501-3p | 63.8 | 18.94 | 46.95 | 30.89 | 37.4 | 62.6 | 63.47 | 58.67 | 60.24 | 53.62 |
| hsa-miR-1285-3p | 68.7 | 13.53 | 63.28 | 47.08 | 42.38 | 44.43 | 49.59 | 51.14 | 42.67 | 73.48 |
| hsa-miR-597 | 39.26 | 33.83 | 63.28 | 47.08 | 33.66 | 60.58 | 59.5 | 52.65 | 42.67 | 63.55 |
| hsa-miR-128 | 83.43 | 31.12 | 46.95 | 27.95 | 32.41 | 42.41 | 75.37 | 63.18 | 42.67 | 49.65 |
| hsa-miR-664-3p | 63.8 | 29.77 | 55.12 | 33.84 | 52.36 | 40.39 | 57.52 | 58.67 | 67.77 | 35.75 |
| hsa-miR-1914-5p | 73.61 | 18.94 | 51.03 | 29.42 | 39.89 | 58.56 | 57.52 | 51.14 | 55.22 | 59.58 |
| hsa-miR-1179 | 58.89 | 25.71 | 61.24 | 33.84 | 31.16 | 48.47 | 45.62 | 49.64 | 77.81 | 61.57 |
| hsa-miR-708-5p | 73.61 | 23 | 59.2 | 35.31 | 29.92 | 60.58 | 53.55 | 40.61 | 55.22 | 61.57 |
| hsa-miR-181b-5p+hsa-miR-181d | 73.61 | 25.71 | 71.45 | 35.31 | 29.92 | 44.43 | 61.49 | 48.14 | 52.71 | 49.65 |
| hsa-miR-584-5p | 68.7 | 20.3 | 53.07 | 27.95 | 47.37 | 56.54 | 55.54 | 45.13 | 67.77 | 49.65 |
| hsa-miR-30d-5p | 49.07 | 24.36 | 69.41 | 29.42 | 43.63 | 66.64 | 49.59 | 55.66 | 32.63 | 71.5 |
| hsa-miR-127-5p | 44.17 | 17.59 | 69.41 | 27.95 | 33.66 | 64.62 | 51.57 | 39.11 | 75.3 | 67.53 |
| hsa-miR-181c-5p | 88.33 | 18.94 | 32.66 | 35.31 | 33.66 | 52.5 | 63.47 | 43.62 | 62.75 | 59.58 |
| hsa-miR-543 | 73.61 | 32.48 | 38.79 | 39.72 | 23.69 | 54.52 | 45.62 | 49.64 | 72.79 | 59.58 |
| hsa-miR-645 | 39.26 | 17.59 | 65.32 | 41.19 | 46.12 | 44.43 | 65.45 | 57.16 | 50.2 | 63.55 |
| hsa-miR-582-5p | 58.89 | 24.36 | 38.79 | 25.01 | 34.9 | 50.48 | 57.52 | 55.66 | 72.79 | 71.5 |
| hsa-miR-130b-3p | 44.17 | 21.65 | 40.83 | 38.25 | 39.89 | 50.48 | 63.47 | 58.67 | 72.79 | 59.58 |
| hsa-miR-637 | 78.52 | 23 | 48.99 | 27.95 | 47.37 | 34.33 | 53.55 | 48.14 | 50.2 | 75.47 |
| hsa-miR-299-3p | 63.8 | 21.65 | 53.07 | 42.66 | 31.16 | 66.64 | 69.42 | 37.61 | 45.18 | 55.61 |
| hsa-miR-551b-3p | 34.35 | 29.77 | 57.16 | 44.13 | 37.4 | 68.66 | 55.54 | 54.15 | 47.69 | 57.6 |
| hsa-miR-449b-5p | 73.61 | 31.12 | 57.16 | 29.42 | 28.67 | 80.78 | 45.62 | 40.61 | 47.69 | 51.64 |
| hsa-miR-3185 | 58.89 | 31.12 | 51.03 | 44.13 | 33.66 | 52.5 | 61.49 | 48.14 | 47.69 | 57.6 |
| hsa-miR-425-5p | 39.26 | 10.83 | 46.95 | 48.55 | 36.15 | 80.78 | 47.6 | 49.64 | 70.28 | 55.61 |
| hsa-miR-1267 | 93.24 | 40.6 | 53.07 | 36.78 | 37.4 | 44.43 | 47.6 | 37.61 | 45.18 | 49.65 |
| hsa-miR-1225-3p | 107.96 | 43.3 | 42.87 | 27.95 | 37.4 | 36.35 | 51.57 | 34.6 | 42.67 | 59.58 |
| hsa-miR-1280 | 53.98 | 14.88 | 71.45 | 19.12 | 33.66 | 66.64 | 53.55 | 63.18 | 47.69 | 59.58 |
| hsa-miR-1294 | 83.43 | 16.24 | 30.62 | 41.19 | 37.4 | 72.7 | 45.62 | 51.14 | 80.31 | 23.83 |
| hsa-miR-920 | 78.52 | 21.65 | 67.36 | 41.19 | 18.7 | 36.35 | 53.55 | 48.14 | 55.22 | 61.57 |
| hsa-miR-921 | 58.89 | 25.71 | 53.07 | 39.72 | 41.14 | 60.58 | 43.64 | 52.65 | 45.18 | 61.57 |
| hsa-miR-217 | 107.96 | 25.71 | 32.66 | 27.95 | 33.66 | 62.6 | 35.7 | 49.64 | 50.2 | 55.61 |
| hsa-miR-1227 | 34.35 | 13.53 | 55.12 | 36.78 | 28.67 | 62.6 | 43.64 | 57.16 | 70.28 | 79.44 |
| hsa-miR-302c-3p | 83.43 | 23 | 48.99 | 25.01 | 32.41 | 58.56 | 53.55 | 46.63 | 47.69 | 61.57 |
| hsa-miR-4521 | 58.89 | 25.71 | 32.66 | 38.25 | 41.14 | 44.43 | 67.44 | 46.63 | 50.2 | 75.47 |
| hsa-miR-526b-5p | 98.15 | 27.06 | 46.95 | 30.89 | 32.41 | 38.37 | 35.7 | 58.67 | 35.14 | 77.46 |
| hsa-miR-491-3p | 29.44 | 23 | 79.61 | 38.25 | 31.16 | 40.39 | 59.5 | 46.63 | 62.75 | 69.51 |
| hsa-miR-4484 | 39.26 | 17.59 | 63.28 | 35.31 | 31.16 | 66.64 | 65.45 | 48.14 | 60.24 | 51.64 |
| hsa-miR-154-5p | 49.07 | 23 | 65.32 | 45.61 | 36.15 | 46.45 | 63.47 | 49.64 | 57.73 | 41.71 |
| hsa-miR-339-5p | 78.52 | 14.88 | 53.07 | 22.07 | 28.67 | 50.48 | 55.54 | 52.65 | 47.69 | 73.48 |
| hsa-miR-1912 | 98.15 | 46.01 | 36.74 | 26.48 | 31.16 | 30.29 | 63.47 | 33.09 | 55.22 | 55.61 |
| hsa-miR-193a-5p | 78.52 | 33.83 | 38.79 | 25.01 | 29.92 | 52.5 | 61.49 | 48.14 | 47.69 | 59.58 |
| hsa-miR-499b-3p | 68.7 | 17.59 | 69.41 | 36.78 | 32.41 | 60.58 | 57.52 | 39.11 | 45.18 | 47.67 |
| hsa-miR-378h | 63.8 | 23 | 55.12 | 30.89 | 49.86 | 40.39 | 37.69 | 51.14 | 52.71 | 69.51 |
| hsa-miR-24-3p | 39.26 | 25.71 | 53.07 | 45.61 | 44.88 | 56.54 | 61.49 | 42.12 | 57.73 | 47.67 |
| hsa-miR-1249 | 68.7 | 25.71 | 61.24 | 41.19 | 34.9 | 48.47 | 39.67 | 49.64 | 40.16 | 63.55 |
| hsa-miR-362-3p | 63.8 | 23 | 75.53 | 33.84 | 28.67 | 52.5 | 51.57 | 43.62 | 52.71 | 47.67 |
| hsa-miR-1245b-3p | 68.7 | 35.18 | 55.12 | 33.84 | 41.14 | 44.43 | 63.47 | 37.61 | 45.18 | 47.67 |
| hsa-miR-323a-5p | 78.52 | 20.3 | 34.7 | 35.31 | 36.15 | 54.52 | 49.59 | 37.61 | 57.73 | 67.53 |
| hsa-miR-151b | 58.89 | 24.36 | 42.87 | 35.31 | 34.9 | 62.6 | 63.47 | 37.61 | 50.2 | 61.57 |
| hsa-miR-572 | 73.61 | 31.12 | 32.66 | 32.36 | 44.88 | 46.45 | 55.54 | 51.14 | 60.24 | 43.69 |
| hsa-miR-204-5p | 103.06 | 21.65 | 34.7 | 29.42 | 31.16 | 44.43 | 65.45 | 61.67 | 25.1 | 53.62 |
| hsa-miR-3144-5p | 103.06 | 23 | 48.99 | 20.6 | 39.89 | 50.48 | 31.74 | 48.14 | 42.67 | 61.57 |
| hsa-miR-299-5p | 44.17 | 18.94 | 61.24 | 38.25 | 32.41 | 40.39 | 63.47 | 49.64 | 50.2 | 69.51 |
| hsa-miR-99a-5p | 63.8 | 18.94 | 67.36 | 30.89 | 29.92 | 48.47 | 41.65 | 48.14 | 40.16 | 77.46 |
| hsa-miR-802 | 73.61 | 27.06 | 61.24 | 41.19 | 49.86 | 42.41 | 31.74 | 46.63 | 35.14 | 57.6 |
| hsa-miR-619 | 122.69 | 17.59 | 34.7 | 23.54 | 34.9 | 44.43 | 55.54 | 40.61 | 35.14 | 55.61 |
| hsa-miR-520g | 78.52 | 23 | 46.95 | 51.49 | 28.67 | 40.39 | 65.45 | 33.09 | 42.67 | 53.62 |
| hsa-miR-3180-3p | 73.61 | 17.59 | 53.07 | 14.71 | 41.14 | 36.35 | 81.32 | 55.66 | 37.65 | 49.65 |
| hsa-miR-618 | 98.15 | 23 | 59.2 | 29.42 | 23.69 | 46.45 | 43.64 | 42.12 | 50.2 | 43.69 |
| hsa-miR-4792 | 78.52 | 27.06 | 57.16 | 30.89 | 36.15 | 46.45 | 53.55 | 31.59 | 37.65 | 59.58 |
| hsa-miR-1265 | 132.5 | 32.48 | 30.62 | 19.12 | 22.44 | 48.47 | 45.62 | 33.09 | 52.71 | 37.74 |
| hsa-miR-509-3p | 68.7 | 33.83 | 55.12 | 36.78 | 28.67 | 54.52 | 39.67 | 37.61 | 42.67 | 53.62 |
| hsa-miR-378d | 39.26 | 17.59 | 51.03 | 39.72 | 47.37 | 52.5 | 53.55 | 39.11 | 55.22 | 55.61 |
| hsa-miR-671-3p | 73.61 | 13.53 | 42.87 | 33.84 | 26.18 | 60.58 | 43.64 | 42.12 | 52.71 | 57.6 |
| hsa-miR-642a-5p | 73.61 | 27.06 | 48.99 | 27.95 | 24.93 | 48.47 | 37.69 | 49.64 | 52.71 | 55.61 |
| hsa-miR-500b | 83.43 | 20.3 | 34.7 | 41.19 | 32.41 | 40.39 | 57.52 | 48.14 | 42.67 | 45.68 |
| hsa-miR-1269a | 83.43 | 23 | 34.7 | 32.36 | 49.86 | 46.45 | 29.75 | 58.67 | 37.65 | 49.65 |
| hsa-miR-377-3p | 49.07 | 14.88 | 46.95 | 33.84 | 34.9 | 46.45 | 53.55 | 45.13 | 70.28 | 49.65 |
| hsa-miR-122-5p | 73.61 | 27.06 | 42.87 | 22.07 | 31.16 | 54.52 | 47.6 | 37.61 | 57.73 | 47.67 |
| hsa-miR-595 | 44.17 | 28.42 | 46.95 | 32.36 | 34.9 | 46.45 | 57.52 | 40.61 | 52.71 | 57.6 |
| hsa-miR-490-3p | 58.89 | 13.53 | 55.12 | 22.07 | 36.15 | 44.43 | 57.52 | 37.61 | 57.73 | 57.6 |
| hsa-miR-876-5p | 49.07 | 23 | 46.95 | 44.13 | 37.4 | 44.43 | 59.5 | 57.16 | 25.1 | 53.62 |
| hsa-miR-29b-3p | 68.7 | 40.6 | 30.62 | 36.78 | 33.66 | 32.31 | 45.62 | 51.14 | 62.75 | 37.74 |
| hsa-miR-125a-5p | 68.7 | 12.18 | 53.07 | 29.42 | 34.9 | 30.29 | 43.64 | 57.16 | 52.71 | 57.6 |
| hsa-miR-361-5p | 68.7 | 25.71 | 48.99 | 25.01 | 27.43 | 64.62 | 39.67 | 48.14 | 42.67 | 47.67 |
| hsa-miR-98 | 78.52 | 24.36 | 42.87 | 32.36 | 29.92 | 56.54 | 41.65 | 39.11 | 32.63 | 57.6 |
| hsa-miR-611 | 19.63 | 17.59 | 65.32 | 45.61 | 32.41 | 52.5 | 65.45 | 33.09 | 40.16 | 63.55 |
| hsa-miR-199b-5p | 44.17 | 17.59 | 59.2 | 22.07 | 24.93 | 60.58 | 67.44 | 37.61 | 55.22 | 45.68 |
| hsa-miR-490-5p | 58.89 | 16.24 | 44.91 | 22.07 | 27.43 | 54.52 | 47.6 | 34.6 | 77.81 | 49.65 |
| hsa-miR-662 | 49.07 | 24.36 | 34.7 | 39.72 | 27.43 | 58.56 | 59.5 | 42.12 | 50.2 | 47.67 |
| hsa-miR-148a-3p | 68.7 | 29.77 | 30.62 | 17.65 | 24.93 | 22.21 | 37.69 | 30.08 | 135.53 | 35.75 |
| hsa-miR-1269b | 117.78 | 32.48 | 44.91 | 14.71 | 23.69 | 30.29 | 39.67 | 36.1 | 37.65 | 55.61 |
| hsa-miR-508-3p | 39.26 | 24.36 | 63.28 | 41.19 | 37.4 | 38.37 | 49.59 | 33.09 | 50.2 | 55.61 |
| hsa-miR-1207-3p | 49.07 | 20.3 | 46.95 | 35.31 | 31.16 | 38.37 | 57.52 | 46.63 | 52.71 | 53.62 |
| hsa-miR-628-5p | 49.07 | 25.71 | 28.58 | 27.95 | 37.4 | 56.54 | 63.47 | 43.62 | 55.22 | 43.69 |
| hsa-miR-2277-5p | 9.81 | 12.18 | 61.24 | 41.19 | 42.38 | 48.47 | 63.47 | 67.69 | 25.1 | 55.61 |
| hsa-miR-640 | 78.52 | 32.48 | 59.2 | 22.07 | 21.19 | 26.25 | 41.65 | 45.13 | 42.67 | 57.6 |
| hsa-miR-1278 | 63.8 | 27.06 | 48.99 | 36.78 | 31.16 | 54.52 | 45.62 | 39.11 | 37.65 | 41.71 |
| hsa-miR-30c-5p | 73.61 | 24.36 | 40.83 | 23.54 | 27.43 | 38.37 | 53.55 | 33.09 | 40.16 | 69.51 |
| hsa-miR-455-5p | 53.98 | 23 | 53.07 | 27.95 | 32.41 | 32.31 | 33.72 | 45.13 | 80.31 | 37.74 |
| hsa-miR-147a | 29.44 | 12.18 | 44.91 | 32.36 | 33.66 | 44.43 | 65.45 | 48.14 | 55.22 | 53.62 |
| hsa-miR-129-5p | 53.98 | 23 | 42.87 | 29.42 | 27.43 | 36.35 | 39.67 | 54.15 | 57.73 | 53.62 |
| hsa-miR-186-5p | 78.52 | 18.94 | 40.83 | 29.42 | 16.21 | 50.48 | 43.64 | 51.14 | 35.14 | 53.62 |
| hsa-miR-18b-5p | 107.96 | 18.94 | 38.79 | 20.6 | 22.44 | 38.37 | 37.69 | 43.62 | 50.2 | 37.74 |
| hsa-miR-92a-3p | 73.61 | 25.71 | 28.58 | 29.42 | 27.43 | 44.43 | 39.67 | 48.14 | 47.69 | 51.64 |
| hsa-miR-616-3p | 58.89 | 27.06 | 55.12 | 35.31 | 26.18 | 42.41 | 45.62 | 48.14 | 37.65 | 39.72 |
| hsa-miR-324-3p | 73.61 | 16.24 | 38.79 | 22.07 | 23.69 | 58.56 | 33.72 | 36.1 | 62.75 | 49.65 |
| hsa-miR-513c-5p | 24.54 | 10.83 | 79.61 | 42.66 | 43.63 | 52.5 | 37.69 | 43.62 | 20.08 | 59.58 |
| hsa-miR-206 | 83.43 | 23 | 51.03 | 33.84 | 26.18 | 34.33 | 71.4 | 34.6 | 25.1 | 31.78 |
| hsa-miR-509-5p | 63.8 | 28.42 | 51.03 | 22.07 | 32.41 | 42.41 | 43.64 | 40.61 | 50.2 | 39.72 |
| hsa-miR-652-3p | 98.15 | 18.94 | 51.03 | 20.6 | 27.43 | 44.43 | 35.7 | 37.61 | 22.59 | 57.6 |
| hsa-miR-620 | 83.43 | 10.83 | 30.62 | 25.01 | 24.93 | 54.52 | 41.65 | 30.08 | 60.24 | 51.64 |
| hsa-miR-92b-3p | 44.17 | 29.77 | 40.83 | 25.01 | 29.92 | 26.25 | 59.5 | 45.13 | 45.18 | 65.54 |
| hsa-miR-3192 | 24.54 | 8.12 | 65.32 | 22.07 | 27.43 | 36.35 | 49.59 | 45.13 | 62.75 | 69.51 |
| hsa-miR-513b | 68.7 | 27.06 | 61.24 | 29.42 | 21.19 | 18.17 | 33.72 | 30.08 | 65.26 | 55.61 |
| hsa-miR-374c-5p | 39.26 | 27.06 | 55.12 | 33.84 | 26.18 | 52.5 | 51.57 | 40.61 | 37.65 | 45.68 |
| hsa-miR-433 | 107.96 | 33.83 | 28.58 | 23.54 | 21.19 | 34.33 | 43.64 | 33.09 | 52.71 | 29.79 |
| hsa-miR-2054 | 53.98 | 12.18 | 40.83 | 38.25 | 38.64 | 38.37 | 49.59 | 43.62 | 55.22 | 37.74 |
| hsa-miR-423-5p | 58.89 | 20.3 | 34.7 | 23.54 | 22.44 | 50.48 | 47.6 | 52.65 | 55.22 | 41.71 |
| hsa-miR-378g | 68.7 | 25.71 | 32.66 | 25.01 | 27.43 | 28.27 | 51.57 | 45.13 | 37.65 | 63.55 |
| hsa-miR-3154 | 63.8 | 24.36 | 42.87 | 41.19 | 27.43 | 28.27 | 45.62 | 42.12 | 40.16 | 49.65 |
| hsa-miR-556-3p | 39.26 | 24.36 | 36.74 | 26.48 | 27.43 | 40.39 | 37.69 | 60.17 | 55.22 | 55.61 |
| hsa-miR-136-5p | 29.44 | 18.94 | 40.83 | 33.84 | 39.89 | 60.58 | 53.55 | 55.66 | 20.08 | 49.65 |
| hsa-miR-509-3-5p | 34.35 | 14.88 | 40.83 | 33.84 | 42.38 | 42.41 | 45.62 | 45.13 | 60.24 | 39.72 |
| hsa-miR-1295a | 49.07 | 16.24 | 42.87 | 25.01 | 36.15 | 40.39 | 47.6 | 46.63 | 47.69 | 47.67 |
| hsa-miR-138-5p | 53.98 | 9.47 | 32.66 | 29.42 | 24.93 | 40.39 | 59.5 | 40.61 | 42.67 | 63.55 |
| hsa-miR-505-3p | 39.26 | 21.65 | 48.99 | 33.84 | 23.69 | 30.29 | 47.6 | 42.12 | 52.71 | 53.62 |
| hsa-miR-4443 | 49.07 | 1.35 | 2.04 | 1.47 | 1.25 | 131.26 | 1.98 | 45.13 | 105.41 | 53.62 |
| hsa-miR-636 | 83.43 | 18.94 | 36.74 | 23.54 | 21.19 | 36.35 | 45.62 | 43.62 | 42.67 | 39.72 |
| hsa-miR-133a | 73.61 | 9.47 | 44.91 | 29.42 | 31.16 | 32.31 | 49.59 | 33.09 | 27.61 | 59.58 |
| hsa-miR-323b-5p | 44.17 | 16.24 | 44.91 | 36.78 | 33.66 | 50.48 | 27.77 | 48.14 | 35.14 | 51.64 |
| hsa-miR-548f | 83.43 | 9.47 | 34.7 | 27.95 | 23.69 | 40.39 | 35.7 | 61.67 | 30.12 | 41.71 |
| hsa-miR-365a-3p | 39.26 | 14.88 | 46.95 | 27.95 | 32.41 | 40.39 | 51.57 | 39.11 | 35.14 | 59.58 |
| hsa-miR-1184 | 34.35 | 20.3 | 44.91 | 36.78 | 23.69 | 34.33 | 45.62 | 36.1 | 45.18 | 65.54 |
| hsa-miR-146b-5p | 58.89 | 16.24 | 34.7 | 23.54 | 26.18 | 42.41 | 55.54 | 43.62 | 35.14 | 49.65 |
| hsa-miR-523-3p | 44.17 | 25.71 | 46.95 | 27.95 | 21.19 | 38.37 | 61.49 | 43.62 | 37.65 | 37.74 |
| hsa-miR-1229 | 34.35 | 13.53 | 55.12 | 30.89 | 32.41 | 46.45 | 47.6 | 37.61 | 50.2 | 35.75 |
| hsa-miR-130a-3p | 49.07 | 17.59 | 51.03 | 30.89 | 28.67 | 36.35 | 51.57 | 36.1 | 37.65 | 41.71 |
| hsa-miR-362-5p | 63.8 | 17.59 | 53.07 | 26.48 | 24.93 | 38.37 | 41.65 | 31.59 | 37.65 | 43.69 |
| hsa-miR-1293 | 68.7 | 17.59 | 38.79 | 33.84 | 24.93 | 42.41 | 53.55 | 21.06 | 42.67 | 33.76 |
| hsa-miR-19a-3p | 49.07 | 20.3 | 46.95 | 33.84 | 18.7 | 46.45 | 29.75 | 31.59 | 60.24 | 37.74 |
| hsa-miR-379-5p | 78.52 | 23 | 36.74 | 27.95 | 27.43 | 34.33 | 53.55 | 33.09 | 20.08 | 39.72 |
| hsa-miR-184 | 49.07 | 23 | 34.7 | 22.07 | 27.43 | 44.43 | 51.57 | 28.58 | 45.18 | 47.67 |
| hsa-miR-1261 | 39.26 | 20.3 | 55.12 | 27.95 | 18.7 | 32.31 | 41.65 | 48.14 | 47.69 | 41.71 |
| hsa-miR-1260a | 83.43 | 16.24 | 40.83 | 35.31 | 26.18 | 24.23 | 41.65 | 30.08 | 50.2 | 19.86 |
| hsa-miR-548u | 93.24 | 20.3 | 32.66 | 19.12 | 26.18 | 30.29 | 31.74 | 25.57 | 40.16 | 47.67 |
| hsa-miR-609 | 49.07 | 16.24 | 48.99 | 25.01 | 26.18 | 40.39 | 45.62 | 42.12 | 40.16 | 29.79 |
| hsa-miR-1469 | 39.26 | 5.41 | 44.91 | 22.07 | 18.7 | 30.29 | 55.54 | 45.13 | 52.71 | 47.67 |
| hsa-miR-1301 | 39.26 | 39.24 | 36.74 | 30.89 | 26.18 | 42.41 | 21.82 | 30.08 | 42.67 | 51.64 |
| hsa-miR-1243 | 39.26 | 10.83 | 48.99 | 22.07 | 27.43 | 28.27 | 49.59 | 34.6 | 40.16 | 57.6 |
| hsa-miR-617 | 78.52 | 24.36 | 28.58 | 13.24 | 31.16 | 34.33 | 25.78 | 34.6 | 47.69 | 39.72 |
| hsa-miR-1915-3p | 39.26 | 21.65 | 42.87 | 20.6 | 28.67 | 32.31 | 47.6 | 43.62 | 30.12 | 43.69 |
| hsa-miR-497-5p | 78.52 | 24.36 | 42.87 | 20.6 | 23.69 | 30.29 | 35.7 | 28.58 | 37.65 | 25.82 |
| hsa-miR-887 | 49.07 | 24.36 | 32.66 | 32.36 | 34.9 | 48.47 | 31.74 | 27.08 | 40.16 | 25.82 |
| hsa-miR-548ac | 58.89 | 18.94 | 32.66 | 26.48 | 36.15 | 30.29 | 27.77 | 34.6 | 42.67 | 37.74 |
| hsa-miR-548b-5p | 34.35 | 17.59 | 40.83 | 14.71 | 24.93 | 46.45 | 43.64 | 27.08 | 52.71 | 43.69 |
| hsa-miR-371a-5p | 29.44 | 8.12 | 34.7 | 25.01 | 41.14 | 40.39 | 39.67 | 55.66 | 30.12 | 41.71 |
| hsa-miR-874 | 24.54 | 20.3 | 34.7 | 27.95 | 21.19 | 46.45 | 49.59 | 30.08 | 32.63 | 53.62 |
| hsa-miR-940 | 34.35 | 23 | 46.95 | 22.07 | 19.95 | 50.48 | 31.74 | 49.64 | 25.1 | 37.74 |
| hsa-miR-2115-5p | 34.35 | 16.24 | 42.87 | 19.12 | 34.9 | 36.35 | 37.69 | 39.11 | 37.65 | 41.71 |
| hsa-miR-532-3p | 73.61 | 16.24 | 24.5 | 16.18 | 27.43 | 36.35 | 33.72 | 42.12 | 27.61 | 41.71 |
| hsa-miR-1260b | 73.61 | 14.88 | 38.79 | 25.01 | 17.45 | 38.37 | 31.74 | 25.57 | 40.16 | 33.76 |
| hsa-miR-1306-3p | 73.61 | 24.36 | 28.58 | 19.12 | 21.19 | 32.31 | 37.69 | 40.61 | 17.57 | 37.74 |
| hsa-miR-181a-5p | 53.98 | 12.18 | 40.83 | 14.71 | 26.18 | 34.33 | 49.59 | 24.07 | 35.14 | 41.71 |
| hsa-miR-1247-5p | 53.98 | 24.36 | 40.83 | 29.42 | 19.95 | 36.35 | 29.75 | 33.09 | 25.1 | 39.72 |
| hsa-miR-642b-3p | 78.52 | 13.53 | 20.41 | 19.12 | 29.92 | 42.41 | 33.72 | 27.08 | 40.16 | 25.82 |
| hsa-miR-326 | 78.52 | 18.94 | 26.54 | 20.6 | 19.95 | 28.27 | 35.7 | 27.08 | 40.16 | 33.76 |
| hsa-miR-539-5p | 24.54 | 28.42 | 42.87 | 27.95 | 16.21 | 40.39 | 41.65 | 42.12 | 22.59 | 41.71 |
| hsa-miR-520e | 49.07 | 17.59 | 40.83 | 16.18 | 14.96 | 36.35 | 49.59 | 31.59 | 40.16 | 29.79 |
| hsa-miR-615-3p | 29.44 | 13.53 | 44.91 | 23.54 | 21.19 | 42.41 | 55.54 | 37.61 | 25.1 | 31.78 |
| hsa-miR-3195 | 53.98 | 25.71 | 36.74 | 22.07 | 18.7 | 24.23 | 41.65 | 19.56 | 32.63 | 49.65 |
| hsa-miR-649 | 49.07 | 14.88 | 42.87 | 29.42 | 21.19 | 44.43 | 27.77 | 27.08 | 25.1 | 41.71 |
| hsa-miR-1268b | 68.7 | 21.65 | 32.66 | 20.6 | 22.44 | 30.29 | 19.83 | 30.08 | 32.63 | 33.76 |
| hsa-miR-296-5p | 34.35 | 17.59 | 32.66 | 32.36 | 36.15 | 28.27 | 21.82 | 33.09 | 42.67 | 31.78 |
| hsa-miR-1976 | 53.98 | 13.53 | 24.5 | 25.01 | 19.95 | 38.37 | 29.75 | 36.1 | 22.59 | 45.68 |
| hsa-miR-1245a | 83.43 | 10.83 | 34.7 | 25.01 | 16.21 | 22.21 | 37.69 | 24.07 | 30.12 | 19.86 |
| hsa-miR-1910 | 53.98 | 31.12 | 26.54 | 22.07 | 18.7 | 28.27 | 35.7 | 25.57 | 32.63 | 27.8 |
| hsa-miR-873-5p | 24.54 | 18.94 | 28.58 | 20.6 | 22.44 | 34.33 | 27.77 | 25.57 | 52.71 | 45.68 |
| hsa-miR-555 | 103.06 | 20.3 | 16.33 | 11.77 | 8.73 | 30.29 | 21.82 | 25.57 | 27.61 | 31.78 |
| hsa-miR-517b-3p | 83.43 | 24.36 | 26.54 | 20.6 | 14.96 | 20.19 | 23.8 | 28.58 | 20.08 | 33.76 |
| hsa-miR-1913 | 78.52 | 16.24 | 22.45 | 13.24 | 22.44 | 24.23 | 35.7 | 27.08 | 22.59 | 27.8 |
| hsa-miR-150-5p | 24.54 | 17.59 | 22.45 | 23.54 | 26.18 | 34.33 | 39.67 | 31.59 | 35.14 | 33.76 |
| hsa-miR-1273c | 49.07 | 37.89 | 20.41 | 19.12 | 16.21 | 16.16 | 33.72 | 31.59 | 30.12 | 31.78 |
| hsa-miR-190b | 39.26 | 14.88 | 36.74 | 19.12 | 18.7 | 34.33 | 21.82 | 24.07 | 25.1 | 43.69 |
| hsa-miR-103a-3p | 19.63 | 12.18 | 34.7 | 17.65 | 18.7 | 48.47 | 25.78 | 28.58 | 37.65 | 33.76 |
| hsa-miR-1273f | 49.07 | 24.36 | 22.45 | 23.54 | 19.95 | 30.29 | 17.85 | 30.08 | 22.59 | 35.75 |
| hsa-miR-1296 | 68.7 | 12.18 | 34.7 | 22.07 | 7.48 | 24.23 | 27.77 | 30.08 | 22.59 | 23.83 |
| hsa-miR-518f-3p | 44.17 | 16.24 | 32.66 | 11.77 | 18.7 | 36.35 | 15.87 | 34.6 | 22.59 | 39.72 |
| hsa-miR-1182 | 34.35 | 13.53 | 22.45 | 17.65 | 27.43 | 30.29 | 37.69 | 39.11 | 12.55 | 35.75 |
| hsa-miR-518d-3p | 34.35 | 20.3 | 32.66 | 19.12 | 21.19 | 22.21 | 29.75 | 22.56 | 27.61 | 37.74 |
| hsa-miR-623 | 19.63 | 17.59 | 18.37 | 14.71 | 26.18 | 42.41 | 23.8 | 36.1 | 37.65 | 27.8 |
| hsa-miR-1224-3p | 29.44 | 16.24 | 30.62 | 25.01 | 22.44 | 22.21 | 25.78 | 31.59 | 30.12 | 27.8 |
| hsa-miR-1253 | 49.07 | 59.54 | 2.04 | 1.47 | 1.25 | 62.6 | 1.98 | 1.5 | 75.3 | 1.99 |
| hsa-miR-602 | 49.07 | 13.53 | 14.29 | 19.12 | 18.7 | 14.14 | 19.83 | 27.08 | 37.65 | 39.72 |
| hsa-miR-373-3p | 34.35 | 16.24 | 42.87 | 19.12 | 7.48 | 20.19 | 21.82 | 21.06 | 20.08 | 29.79 |
| hsa-miR-296-3p | 24.54 | 12.18 | 16.33 | 22.07 | 26.18 | 26.25 | 17.85 | 21.06 | 20.08 | 37.74 |
| hsa-miR-143-3p | 63.8 | 1.35 | 2.04 | 1.47 | 1.25 | 107.03 | 1.98 | 1.5 | 27.61 | 1.99 |
| hsa-miR-4485 | 14.72 | 8.12 | 26.54 | 23.54 | 23.69 | 12.12 | 19.83 | 18.05 | 15.06 | 27.8 |
| hsa-miR-363-3p | 44.17 | 1.35 | 2.04 | 1.47 | 1.25 | 6.06 | 1.98 | 1.5 | 27.61 | 1.99 |
| hsa-miR-549 | 4.91 | 1.35 | 2.04 | 1.47 | 1.25 | 2.02 | 1.98 | 1.5 | 2.51 | 1.99 |
