# Supplementary material for: Airway Secretory microRNAome Changes during Rhinovirus Infection in Early Childhood
Source: PLoS One. 2016 Sep 19;11(9):e0162244. doi: 10.1371/journal.pone.0162244 (PMC5028059; doi:10.1371/journal.pone.0162244)
Supplement: S4 Table — (DOCX) [file pone.0162244.s004.docx]

**Table 4 S hsa-mir-155 Targetome in experimental in vivo human rhinovirus infection**

| Target Gene | Fold Change at 8 hours | Fold Change at 48 hours | Gene Title |
| --- | --- | --- | --- |
| SOCS1 | -1.07593 | 2.681943 | suppressor of cytokine signaling 1 |
| CCL2 | -1.06769 | 2.395998 | chemokine (C-C motif) ligand 2 |
| IL8 | -1.19705 | 2.076395 | interleukin 8 |
| ICAM1 | -1.0978 | 1.832815 | intercellular adhesion molecule 1 |
| MAFB | 1.063059 | 1.716192 | v-maf musculoaponeurotic fibrosarcoma oncogene homolog B (avian) |
| IL6 | -1.03789 | 1.607211 | interleukin 6 (interferon, beta 2) |
| PLAUR | -0.38175 | 1.576933 | plasminogen activator, urokinase receptor |
| MTHFD2 | -1.04609 | 1.544763 | methylenetetrahydrofolate dehydrogenase (NADP+ dependent) 2, methenyltetrahydrofolate cyclohydrolase |
| PNPT1 | 1.058601 | 1.501339 | polyribonucleotide nucleotidyltransferase 1 |
| JUN | -0.52456 | 1.491834 | jun proto-oncogene |
| JUNB | -1.04481 | 1.445657 | jun B proto-oncogene |
| NAMPT | -1.18299 | 1.434043 | nicotinamide phosphoribosyltransferase |
| ARL5B | -0.00027 | 1.417231 | ADP-ribosylation factor-like 5B |
| LAT2 | -1.00517 | 1.413913 | linker for activation of T cells family, member 2 |
| FLT1 | 0.346003 | 1.404947 | fms-related tyrosine kinase 1 |
| FNDC3B | -0.66726 | 1.368666 | fibronectin type III domain containing 3B |
| THBS1 | -1.04169 | 1.367068 | thrombospondin 1 |
| EDN1 | 1.017994 | 1.333348 | endothelin 2 |
| OLR1 | -1.0282 | 1.328898 | oxidized low density lipoprotein (lectin-like) receptor 1 |
| SNTB2 | 1.02538 | 1.307837 | syntrophin, beta 2 (dystrophin-associated protein A1, 59kDa, basic component 2) |
| BACH1 | -1.01681 | 1.304803 | BTB and CNC homology 1, basic leucine zipper transcription factor 1 |
| HK2 | -1.05816 | 1.295465 | hexokinase 2 |
| FAM135A | -1.01579 | 1.278476 | family with sequence similarity 135, member A |
| GLIPR1 | -1.01923 | 1.26615 | GLI pathogenesis-related 1 |
| GNA13 | 1.015016 | 1.262578 | guanine nucleotide binding protein (G protein), alpha 13 |
| CFL2 | 0.016174 | 1.258255 | cofilin 2 (muscle) |
| MYD88 | -1.02482 | 1.257094 | myeloid differentiation primary response 88 |
| VCAM1 | 1.007125 | 1.254822 | vascular cell adhesion molecule 1 |
| B4GALT1 | -1.10107 | 1.253663 | UDP-Gal:betaGlcNAc beta 1,4- galactosyltransferase, polypeptide 1 |
| TNFRSF10A | -1.0394 | 1.248892 | tumor necrosis factor receptor superfamily, member 10a |
| CLIC4 | -1.01481 | 1.248835 | chloride intracellular channel 4 |
| CEBPB | -1.03462 | 1.246484 | CCAAT/enhancer binding protein (C/EBP), beta |
| BCL6 | -1.07139 | 1.244916 | B-cell CLL/lymphoma 6 |
| ZNF207 | -1.07795 | 1.241615 | zinc finger protein 207 |
| FMNL2 | -1.06873 | 1.237683 | formin-like 2 |
| CTLA4 | 1.004572 | 1.22038 | cytotoxic T-lymphocyte-associated protein 5 |
| APAF1 | -1.0721 | 1.219115 | apoptotic peptidase activating factor 1 |
| STAT3 | -1.07188 | 1.217071 | signal transducer and activator of transcription 3 (acute-phase response factor) |
| TNPO1 | -1.02279 | 1.209007 | transportin 1 |
| FLNA | -1.02073 | 1.206729 | filamin A, alpha |
| MEST | 1.040223 | 1.205535 | mesoderm specific transcript |
| SLC38A5 | 1.013782 | 1.203534 | solute carrier family 38, member 5 |
| TNFAIP2 | -1.2116 | 1.202984 | tumor necrosis factor, alpha-induced protein 2 |
| ANKFY1 | -1.01383 | 1.202741 | ankyrin repeat and FYVE domain containing 1 |
| CUTA | 1.009852 | -1.20293 | cutA divalent cation tolerance homolog (E. coli) |
| C12orf10 | 1.028388 | -1.20475 | chromosome 12 open reading frame 10 |
| PSMG1 | 1.040828 | -1.20784 | proteasome (prosome, macropain) assembly chaperone 1 |
| GAPVD1 | 1.004613 | -1.20839 | GTPase activating protein and VPS9 domains 1 |
| FAM177A1 | 1.037715 | -1.20876 | family with sequence similarity 177, member A1 |
| PEBP1 | 0.334605 | -1.21121 | phosphatidylethanolamine binding protein 1 |
| PHF17 | 1.071874 | -1.21181 | PHD finger protein 17 |
| PRKAR2A | 1.043095 | -1.21198 | protein kinase, cAMP-dependent, regulatory, type II, alpha |
| MSI2 | 0.68287 | -1.21513 | musashi RNA-binding protein 2 |
| AKR1C3 | 1.041051 | -1.21619 | aldo-keto reductase family 1, member C3 |
| CAT | 1.030539 | -1.21974 | catalase |
| LDOC1 | 1.035072 | -1.2227 | leucine zipper, down-regulated in cancer 1 |
| TOMM20 | 1.037594 | -1.22354 | translocase of outer mitochondrial membrane 20 homolog (yeast) |
| ALDH5A1 | 1.036393 | -1.22538 | aldehyde dehydrogenase 5 family, member A1 |
| TRAK1 | -0.03034 | -1.22955 |  |
| CYP2U1 | -1.01431 | -1.23421 | cytochrome P450, family 2, subfamily U, polypeptide 1 |
| ATP6V1C1 | 1.044472 | -1.23631 | ATPase, H+ transporting, lysosomal 42kDa, V1 subunit C1 |
| MAVS | 1.050229 | -1.23695 | mitochondrial antiviral signaling protein |
| MECP2 | -1.0875 | -1.23893 | methyl CpG binding protein 2 (Rett syndrome) |
| PAPOLA | 1.029043 | -1.24365 | poly(A) polymerase alpha |
| GNAS | -1.01407 | -1.25007 | GNAS complex locus |
| CAB39L | 1.078758 | -1.26674 | calcium binding protein 39-like |
| STRBP | -0.38097 | -1.26702 | spermatid perinuclear RNA binding protein |
| EOGT | 1.013159 | -1.27221 | EGF domain-specific O-linked N-acetylglucosamine (GlcNAc) transferase |
| PCYOX1 | 1.073394 | -1.27234 | prenylcysteine oxidase 1 |
| ARFIP2 | 1.077469 | -1.28478 | ADP-ribosylation factor interacting protein 2 |
| WRB | 1.067358 | -1.28749 | tryptophan rich basic protein |
| EIF3F | 1.020883 | -1.29593 | eukaryotic translation initiation factor 3, subunit F |
| SIN3A | -1.00246 | -1.30545 | SIN3 transcription regulator homolog A (yeast) |
| PALLD | 0.331823 | -1.30929 | palladin, cytoskeletal associated protein |
| RAD23B | 1.045914 | -1.39172 | RAD23 homolog B (S. cerevisiae) |
| SLC27A2 | 1.043791 | -1.3943 | solute carrier family 27 (fatty acid transporter), member 2 |
| DPP7 | 1.088903 | -1.41367 | dipeptidyl-peptidase 7 |
| GCLC | 1.00839 | -1.43091 | glutamate-cysteine ligase, catalytic subunit |
| MATR3 | -1.03527 | -1.47014 | matrin 3 |
| METTL7A | 1.02419 | -1.53499 | methyltransferase like 7A |
| CD36 | 0.530133 | -1.65648 | CD36 molecule (thrombospondin receptor) |
